# Identification of genetic relationships and subspecies signatures in *Xylella fastidiosa*

**DOI:** 10.1186/s12864-019-5565-9

**Published:** 2019-03-25

**Authors:** Nicolas Denancé, Martial Briand, Romain Gaborieau, Sylvain Gaillard, Marie-Agnès Jacques

**Affiliations:** IRHS, INRA, AGROCAMPUS-Ouest, Université d’Angers, SFR 4207 QUASAV, 42 rue Georges Morel, 49071 Beaucouzé cedex, France

**Keywords:** 16S rRNA gene, Horizontal gene transfer, Phylogeny, K-mer, SkIf, Taxonomy

## Abstract

**Background:**

The phytopathogenic bacterium *Xylella fastidiosa* was thought to be restricted to the Americas where it infects and kills numerous hosts. Its detection worldwide has been blooming since 2013 in Europe and Asia. Genetically diverse, this species is divided into six subspecies but genetic traits governing this classification are poorly understood.

**Results:**

SkIf (Specific k-mers Identification) was designed and exploited for comparative genomics on a dataset of 46 *X. fastidiosa* genomes, including seven newly sequenced individuals. It was helpful to quickly check the synonymy between strains from different collections. SkIf identified specific SNPs within 16S rRNA sequences that can be employed for predicting the distribution of *Xylella* through data mining. Applied to inter- and intra-subspecies analyses, it identified specific k-mers in genes affiliated to differential gene ontologies. Chemotaxis-related genes more prevalently possess specific k-mers in genomes from subspecies *fastidiosa, morus* and *sandyi* taken as a whole group. In the subspecies *pauca* increased abundance of specific k-mers was found in genes associated with the bacterial cell wall/envelope/plasma membrane. Most often, the k-mer specificity occurred in core genes with non-synonymous SNPs in their sequences in genomes of the other subspecies, suggesting putative impact in the protein functions. The presence of two integrative and conjugative elements (ICEs) was identified, one chromosomic and an entire plasmid in a single strain of *X. fastidiosa* subsp. *pauca*. Finally, a revised taxonomy of *X. fastidiosa* into three major clades defined by the subspecies *pauca* (clade I), *multiplex* (clade II) and the combination of *fastidiosa, morus* and *sandyi* (clade III) was strongly supported by k-mers specifically associated with these subspecies.

**Conclusions:**

SkIf is a robust and rapid software, freely available, that can be dedicated to the comparison of sequence datasets and is applicable to any field of research. Applied to *X. fastidiosa*, an emerging pathogen in Europe*,* it provided an important resource to mine for identifying genetic markers of subspecies to optimize the strategies attempted to limit the pathogen dissemination in novel areas.

**Electronic supplementary material:**

The online version of this article (10.1186/s12864-019-5565-9) contains supplementary material, which is available to authorized users.

## Background

*Xylella fastidiosa* is a species of plant pathogenic bacteria endemic in the Americas, but listed as quarantine pests elsewhere (https://gd.eppo.int/taxon/XYLEFA/categorization). However, since 2013, various cases of emergences have been reported in Europe (Italy, France, Germany and Spain) on large ranges of host plants including olive trees, grapevine, and ornamentals [1–5]. In Italy, assuming that *X. fastidiosa* started spreading in 2010, a recent model approach suggested that it will progress through olive orchards to infect the northernmost recorded orchards within 43.5 years [6]. In France, bacterial introduction was estimated between 1985 and 2001, depending on the modeled scenarios [7, 8]. Several records of *X. fastidiosa* in imported materials (i.e. mostly coffee plants) were also reported over the same period in Europe [9–12].

*X. fastidiosa* is a genetically diverse species that is currently divided into six subspecies (subsp. *fastidiosa, pauca, multiplex, sandyi, morus, tashke*), the four-first being the most damaging and now being found in numerous countries worldwide. But the genetic diversity of the genus *Xylella* is undoubtedly underestimated. Yet another species, *X. taiwanensis*, was recently proposed for the strains causing leaf scorch on nashi pear tree, a disease that was reported more than 25 years ago in Taiwan and initially thought to be caused by a *X. fastidiosa* strain [13]. Recombination is known to drive *X. fastidiosa* evolution and adaptation to novel hosts [11, 14–16]. For example, the subspecies *morus* has been proposed for grouping strains issued from large events of intersubspecific recombination that were associated with a host shift [16]. The recent outbreaks and interception of imported, contaminated materials in Europe as well as investigations in South America also revealed the existence of previously unknown Sequence Types of several *X. fastidiosa* subspecies [2, 9, 11, 17, 18].

Because management and regulations of *X. fastidiosa* outbreaks in France depends on the subspecies of *X. fastidiosa*, it is of major importance to precisely define these subspecies, understand the robustness of these groupings and their meaning in terms of specific or shared genetic material. One way to resolve such a series of interrogations is the achievement of comparative genomics to identify similarities and specificities between groups of individuals. Yet, exploring big datasets is not trivial and requires dedicated bioinformatic tools to be cost- and time-effective. Various applications use k-mers mostly to analyze sequence reads to improve the quality of genome, transcriptome and metagenome assembly [19–26]. K-mer are all the possible substrings of length k that are contained in a nucleotide character string. K-mer-based methods can also be employed on whole genome sequences to taxonomically assign organisms [27, 28]. Moreover, several tools were developed to calculate pairwise relationships, like the average nucleotide identities using blast (ANIb) or MUMmer (ANIm) algorithms, and the tetranucleotide frequency correlation coefficients (TETRA), which can be accessed online through JSpecies [29] or with workstation installation of python3 pyani module [30].

Here, we developed SkIf (Specific k-mers Identification) and applied it to gain a better understanding of *X. fastidiosa* clustering in subspecies through the detection of genomic regions specifically associated with *X. fastidiosa* subspecies. We also used this tool to identify specific k-mers within 16S rRNA gene and assess the occurrences of *X. fastidiosa* in the SILVA database as a first attempt to mine large databases to evaluate the worldwide dispersion of *X. fastidiosa* subspecies.

## Results

### The genome sequence dataset

The dataset used in this study gathered 47 *Xylella* genomes sequences, including 46 *X. fastidiosa* and one *X. taiwanensis* specimen (Table 1). The *X. fastidiosa* subspecies *tashke* could not be included as no strain or genome sequence are available. In some analyses, the three strains belonging to *X. fastidiosa* subsp. *sandyi* were separated into two groups, containing either the original strain Ann-1 (*sandyi*) or the more recently discovered relatives CO33 and CFBP 8356 (*sandyi*-like) both belonging to the unusual *sandyi* ST72 [17]*.* The CFBP 8073 strain, described as an atypical *X. fastidiosa* subsp. *fastidiosa* strain [11] was either included or not for analyses of this subspecies. The *X. fastidiosa* genome sequences involve 39 publicly available ones and seven newly individuals. The strains sequenced in this work were selected based on their country of isolation, genetic diversity and host range, inferred from their belonging to the subspecies *fastidiosa* (CFBP 7969, CFBP 7970, CFBP 8071, CFBP 8082, and CFBP 8351), *sandyi* (CFBP 8356) and *multiplex* (CFBP 8078). Genome sequence characteristics are described in Table 2.

**Table 1:** List of the 47 *Xylella* genome sequences used in this study

Genotype	Strain	ST^a^	Host plant	Country (year)^b^	Accession number	Reference
*X. fastidiosa*	ATCC 35879	2	*Vitis vinifera*	FL, USA (1987)	NZ_JQAP00000000	Unpublished
subsp.	DSM 10026	2	*Vitis vinifera*	FL, USA (1987)	NZ_FQWN01000006	Unpublished
*fastidiosa*	CFBP 7969	2	*Vitis rotundifolia*	NC, USA (1985)	PHFQ00000000	This study
	CFBP 7970	2	*Vitis vinifera*	FL, USA (1987)	PHFR00000000	This study
	CFBP 8071	1	*Prunus dulcis*	CA, USA (1987)	PHFP00000000	This study
	CFBP 8073	75	*Coffea canephora*	Mexico (2012)	LKES00000000	[11]
	CFBP 8082	2	*Ambrosia artemifolia*	FL, USA (1983)	PHFT00000000	This study
	CFBP 8351	1	*Vitis* sp.	CA, USA (1993)	PHFU00000000	This study
	EB92–1	1	*Sambucus nigra*	FL, USA (1992)	AFDJ00000000	[67]
	GB514	1	*Vitis vinifera*	TX, USA (2007)	NC_017562	[68]
	M23	1	*Prunus dulcis*	CA, USA (2003)	NC_010577	[69]
	Stag’s Leap	1	*Vitis vinifera*	CA, USA (1994)	LSMJ010000	[70]
	Temecula1	1	*Vitis vinifera*	CA, USA 1998)	NC_004556	[58]
*X. f.* subsp.	ATCC 35871	41	*Prunus salicina*	CA, USA (1983)	NZ_AUAJ00000000	Unpublished
*multiplex*	BB01	42	*Vaccinium corymbosum*	GA, USA (2016)	NZ_MPAZ01000000	[71]
	CFBP 8078	51	*Vinca* sp.	FL, USA (1983)	PHFS00000000	This study
	CFBP 8416	7	*Polygala myrtifolia*	COR, FR (2015)	LUYC00000000	[2]
	CFBP 8417	6	*Spartium junceum*	COR, FR (2015)	LUYB00000000	[2]
	CFBP 8418	6	*Spartium junceum*	COR, FR (2015)	LUYA00000000	[2]
	Dixon	6	*Prunus dulcis*	CA, USA (1994)	AAAL00000000	[72]
	Griffin-1	7	*Quercus rubra*	GA, USA (2006)	AVGA00000000	[73]
	M12	7	*Prunus dulcis*	CA, USA (2003)	NC_010513	[69]
	Sy-VA	8	*Platanus occidentalis*	VA, USA (2002)	JMHP00000000	[74]
*X. f.* subsp.	Ann-1	5	*Nerium oleander*	CA, USA (1995)	CP006696	[75]
*sandyi*	CFBP 8356	72	*Coffea arabica*	Costa Rica (2015)	PHFV00000000	This study
	Co33	72	*Coffea arabica*	Costa Rica (2014)	LJZW00000000	[76]
*X. f.* subsp.	Mul0034	30	*Morus alba*	USA (2003)	CP006740	[77]
*morus*	Mul-MD	29	*Morus alba*	MD, USA (2011)	AXDP00000000	[77]
*X. f.* subsp.	32	16	*Coffea arabica*	Brazil (1997)	AWYH00000000	[78]
*pauca*	3124	16	*Coffea* sp.	Brazil (2009)	CP009829	Unpublished
	11,399	12	*Citrus cinensis*	Brazil (1996)	NZ_JNBT01000030	[79]
	6c	14	*Coffea arabica*	Brazil (1997)	AXBS00000000	[78]
	9a5c	13	*Citrus cinensis*	Brazil (1992)	NC_002488	[57]
	CFBP 8072	74	*Coffea Arabica*	Ecuador (2012)	LKDK00000000	[11]
	CoDiRO	53	*Catharanthus roseus* ^*c*^	Italy (2013)	JUJW00000000	[33]
	COF0324	14	*Coffea* sp.	Costa Rica (2006)	LRVG01000000	Unpublished
	COF0407	53	*Coffea* sp.	Costa Rica (2009)	LRVJ00000000	Unpublished
	CVC0251	12	*Citrus cinensis*	Brazil (1999)	LRVE01000000	Unpublished
	CVC0256	12	*Citrus cinensis*	Brazil (1999)	LRVF01000000	Unpublished
	Fb7	69	*Citrus sinensis*	Argentina (1998)	CP010051	Unpublished
	Hib4	70	*Hibiscus fragilis*	Brazil (2000)	CP009885	Unpublished
	J1a12	12	*Citrus* sp.	Brazil (2001)	CP009823	Unpublished
	OLS0478	53	*Nerium oleander*	Costa Rica (2010)	LRVI00000000	Unpublished
	OLS0479	53	*Nerium oleander*	Costa Rica (2010)	LRVH00000000	Unpublished
	Pr8x	14	*Prunus (Plum)*	Brazil (2009)	CP009826	Unpublished
	U24D	13	*Citrus sinensis*	Brazil (2000)	CP009790	Unpublished
*X. taiwanensis*	PLS 229	–	*Pyrus pyrifolia*	Taiwan (−)	JDSQ00000000	[80]

^a^Sequence Type determined following the MSLT scheme dedicated to *X. fastidiosa* [52]

^b^Exact year of isolation or oldest year of literature citing the stain

^c^DNA was recovered from infected periwinkle. This genome is the one of the CoDiRO strain, the agent responsible for the Olive Quick Decline Syndrome in Italy (46)

**Table 2:** List of *Xylella fastidiosa* strains sequenced for this study and genome properties

Strain	Accession	Nb of reads^a^	Cover.^b^	Assemby size (bp)	Nb contigs^c^	N50	Mean size (bp)	Largest (bp)	GC %
CFBP 7969	PHFQ00000000	7,952,452	957x	2,436,752	89	116,341	27,379	445,308	51.48
CFBP 7970	PHFR00000000	8,041,300	968x	2,493,794	93	104,928	26,815	258,911	51.45
CFBP 8071	PHFP00000000	7,606,748	916x	2,489,737	101	104,990	24,651	297,538	51.48
CFBP 8082	PHFT00000000	7,610,344	916x	2,532,132	118	104,927	21,459	301,313	51.51
CFBP 8351	PHFU00000000	8,741,758	1053x	2,479,202	93	104,608	26,658	266,361	51.45
CFBP 8078	PHFS00000000	8,807,962	1060x	2,602,010	191	87,559	13,623	204,167	51.67
CFBP 8356	PHFV00000000	8,088,406	974x	2,541,621	197	93,086	12,902	190,454	51.58

^a^pair-end (301 bp)

^b^Coverage calculated for a mean genome of 2.5 Mb

^c^Larger than 500 bp

### Evaluation of strain synonymy using k-mers

CFBP 7970, the *X. fastidiosa* and *X. fastidiosa* subsp. *fastidiosa* type strain [31], has various synonymous names (ATCC 35879, DSM 10026, LMG17159, http://www.straininfo.net/strains/901514 or http://www.bacterio.net/xylella.html) in other collections, as a result of strain exchanges between the American (ATCC), German (DSMZ), Belgian (LMG), and French (CIRM-CFBP) collections [32]. As no genome sequences were available at the beginning of this study for any of these strains, CFBP 7970 was included in our dataset and its genome was sequenced. Later on, the genome sequences of the strains ATCC 35879 and DSM 10026 were released. Although the genome sequences of the three strains were very similar (99.83–99.97% ANIb), they were not strictly identical (Additional file 1). The use of SkIf identified DNA fragments that were specific to two genome sequences, but absent in the third one, yielding in 95, 192, and 594 k-mers specifically present in the pairs ATCC 35879/DSM 10026, CFBP 7970/DSM 10026, ATCC 35879/CFBP 7970, respectively (Additional file 2). The absence of some mers in genome sequence could be due to sequencing artefacts (e.g. sequencing technology employed, average coverage and assembly methods) that resulted in specific SNPs (Table 3). However, 16 mers (ranging from 29 to 9,178 nt and totalizing 36,845 bp in size) were detected in a single contig (41,458 bp) in CFBP 7970 and into several DSM 10026 contigs but were absent from ATCC 35879 genome sequence (Additional file 2). These sequences shared high identity levels with a plasmid found in multiple *Xylella* subspecies, known as pXF-De Donno (subsp. *pauca* De Donno strain), pXF-RIV5 (subsp. *multiplex* RIV5 strain), pXF-FAS01 (subsp. *fastidiosa* M23 strain) or present but unnamed elsewhere (like in subsp. *pauca* CoDiRO strain and subsp. *multiplex* Dixon strain) [2, 33–35]. But the blast analysis suggested its possible presence, as partial matches (3.8 -6 kb in total) with high but not perfect identity levels (95–99%) were found when searched against the genome sequence of ATCC 35879 (Additional file 2; Additional file 3).

**Table 3:** Properties of genome sequences of strains CFBP 7970, DSM 10026 and ATCC 35879

	CFBP 7970^a^	DSM 10026^b^	ATCC 35879^c^
Sequencing technology	Ilumina MiSeq	Shot gun	Illumina MiSeq
Assembling method	Velvet SOAPdenovo SOAPGapCloser	Not available	CLC Genomic Workbench
Genome size	2,493,794 bp	2,426,538 bp	2,522,328 bp
Number of contigs	93	72	16
Minimal size of contigs	500 bp	1 kb	1.2 kb
Coverage	968x	416x	1380x

^a^ Data from the present study

^b^ More details at: https://www.ncbi.nlm.nih.gov/genome/173?genome_assembly_id=295121

^c^ More details at: https://www.ncbi.nlm.nih.gov/genome/173?genome_assembly_id=212014

### Data mining of the 16S rRNA SILVA database to assign occurrences of *Xylella* sp.

The availability of the 47 *Xylella* genome sequences renders possible the analysis of the allelic diversity of the 16S rRNA marker gene. This housekeeping marker is widely used in bacterial phylogeny and taxonomy studies for various reasons including its vertical inheritance and ubiquity in prokaryotes. It is also commonly used to survey microbial communities and as such is a marker to survey largely the environment. A total of 74 16S rRNA gene sequences was retrieved from our dataset, these either being present in one (*n* = 20) or two copies (*n* = 27) in *X. fastidiosa* and *X. taiwanensis* (Table 4). We detected 19 SNPs (over 1547 nucleotides, 1.22%) specific to *X. taiwanensis* PLS 229 16S rRNA (Additional file 4).

**Table 4:** Repertoire of 16S rRNA gene sequences in 47 genomes of *Xylella* sp

*Xylella* genomes (total; with 1 copy; with 2 copies)	Codes of strains having one copy of 16S rRNA	Codes of strains having two copies of 16S rRNA
X. *fastidiosa* subsp. *fastidiosa* (*n* = 13;3;10)	EB92–1, CFBP 8073, CFBP 8351	ATCC 35879, CFBP 7969, CFBP 7970, CFBP 8071, CFBP 8082, DSM10026, GB514, M23, Stag’s Leap, Temecula1
X. *fastidiosa* subsp. *multiplex* (*n* = 10;8;2)	ATCC 35871, BB01, CFBP 8078, CFBP 8417, CFBP 8418, Dixon, Griffin-1, Sy-VA	CFBP 8416, M12
X. *fastidiosa* subsp. *morus*(*n* = 2;1;1)	Mul-MD	Mul0034
X. *fastidiosa* subsp. *sandyi* (*n* = 3;1;2)	CFBP 8356	Ann-1, CO33
X. *fastidiosa* subsp. *pauca* (*n* = 18;6;12)	CFBP 8072, COF0324, COF0407, OLS0479, Xf6c, Xf32	11,399, 3124, 9a5c, CoDiRO, CVC0251, CVC0256, Fb7, Hib4, J1a12, OLS0478, Pr8x, U24D
X. *taiwanensis* (*n* = 1;1;0)	PLS 229	–

Specific mers were searched for within the *Xylella* genus (i.e., the in-group included the 74 *Xylella* 16S rRNA copies; the out-group included all the SSU sequences from the Silva database other than Xylella-tagged) and the *X. fastidiosa* species (i.e., the genome sequence of *X. taiwanensis* PLS 229 strain was included in the out-group). Five long-mers (referred to as LongXyl#1 to #5) specific to *Xylella* genus and four long-mers (referred to as LongXylefa#1 to #4) specific to the *X. fastidiosa* species were identified. LongXyl (23-43 nt) and LongXylefa (23-31 nt) mers located between positions 212 and 866, and positions 202 and 1013, respectively, which include the V3-V4 hypervariable regions widely used in community profiling approaches (Additional file 5). Specific signatures obtained from eight nucleotide positions in the 16S rRNA alignment discriminated alleles from *X. fastidiosa* subsp. *fastidiosa*, *multiplex*, *morus, sandyi* and *pauca* (Table 5).

**Table 5:** Specific signatures in 16S rRNA nucleotide sequences to discriminate *X. fastidiosa* subspecies

*X. fastidiosa* subsp. (nb of genome sequences)	SNPs at the designed positions^a^							
	75^a^	76	151	455	474	1127	1264	1340
*fastidiosa* (*n* = 13)	C	A	C	G	–	G	A	C
*morus* (*n* = 2)	C	A	C	G	–	T	A	C
*multiplex* (*n* = 10)	C	A	T	A	–	G	G	C
*sandyi* (*n* = 1)^b^	C	A	T	A	T	G	A	C
*sandyi*-like (*n* = 2)^c^	T	A	C	A	–	G	G	C
*pauca* (*n* = 18)	C	G	T	A	–	G	A	T

^a^refers to SNP positions within the alignment of the copies of 16S rRNA (Additional file 4)

^b^refers to Ann-1strain only

^c^refers to strains CFBP 8356 and CO33 strains

The occurrence of these specific mers in 16S rRNA nucleotide sequences was investigated within the SILVA rRNA database (Additional file 4). A large proportion of the sequences retrieved from the SILVA database (*n* = 118/195) covered less than half of the total gene length, and only a minority (*n* = 70/195) covered at least three fourth of the length. After nucleotide alignment, 53 of these sequences including the LongXyl and LongXylefa specific signatures were retained (Additional file 4). Based on their genetic signatures, 51 sequences were assigned to subsp. *multiplex* (*n* = 32), *fastidiosa* (n = 11), *pauca* (*n* = 5), *sandyi* (*n* = 2) and *morus* (n = 1), while two sequences (EU560720.1 and EU560722.1) could not be assigned because they did not cover most of the eight discriminant nucleotide positions (Additional file 5). The validity of this presumptive taxonomic affiliation was consolidated by the description of the sample, which were in adequacy with the current host range of these subspecies. Indeed, samples from subsp. *fastidiosa* mainly come from alfalfa or grapevine, while those from subsp. *multiplex* were collected on various oak species, those from subsp. *sandyi* were isolated from oleander, those from subsp. *pauca* from periwinkle, coffee or olive trees, and the one from subsp. *morus* came from mulberry.

### Identification of allelic variants specific to each *X. fastidiosa* subspecies

Beyond focusing on a single gene (16S rRNA), we applied SkIf to a whole genome-based analysis. Seven groups of strains were defined: *fastidiosa* (two groups), *pauca*, *multiplex*, *morus* and *sandyi* (two groups). The two *fastidiosa* groups differed by the presence/absence of the strain CFBP 8073, while one *sandyi* group included only the original member Ann-1, and the *sandyi*-like group included only CFBP 8356 and CO33 strains. Specific mers were searched for in each group against all the others.

**Fig. 1:**
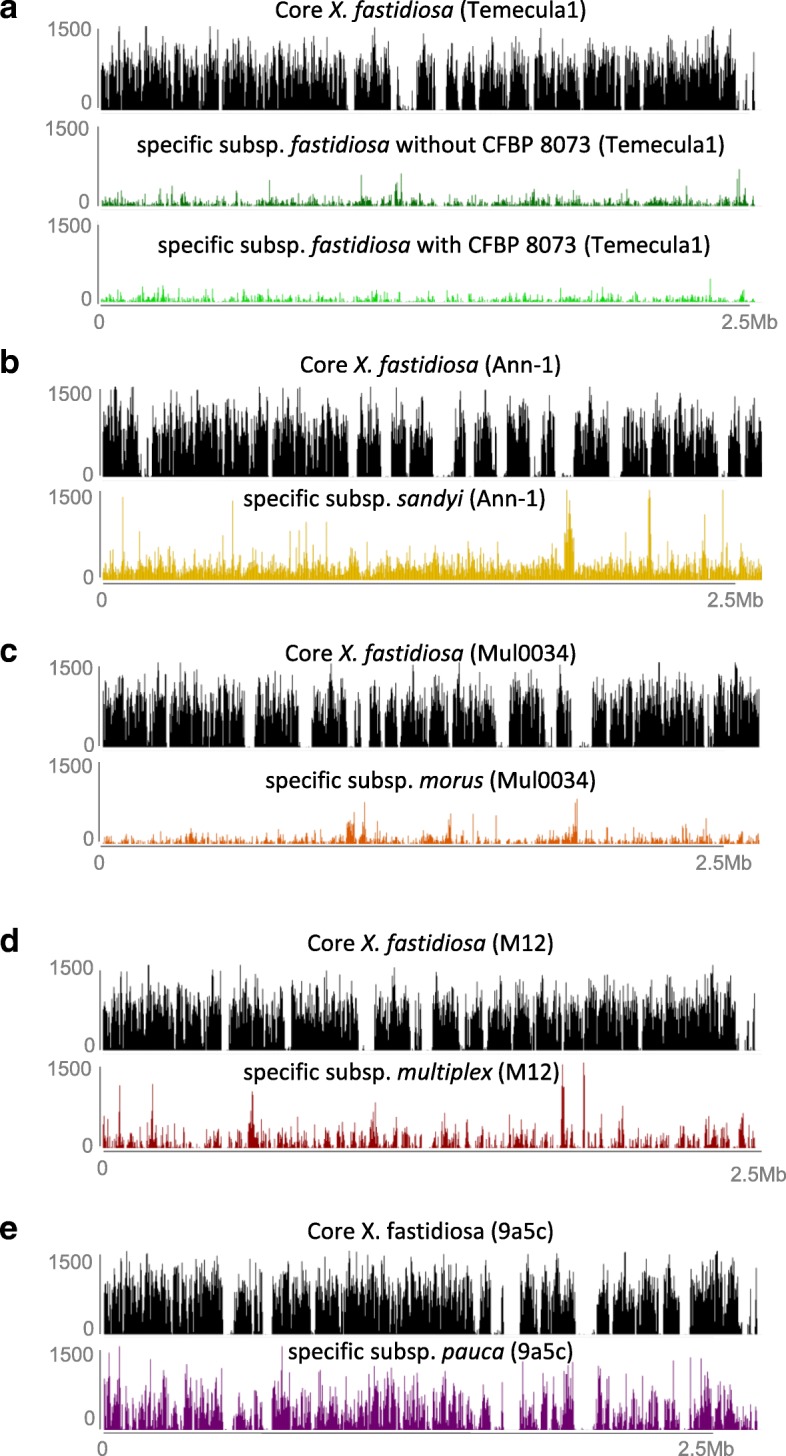
Distribution of k-mers along the *X. fastidiosa* genome sequences. Frequency of core (black) and specific (colored) k-mers mapped onto the genome of reference (mentioned into brackets) of each subspecies. **a** subsp. *fastidiosa* with or without CFBP 8073 strain. **b** subsp. *sandyi*. **c** subsp. *morus.* **d** subsp. *multiplex.* **e** subsp. *pauca*

**Fig. 2:**
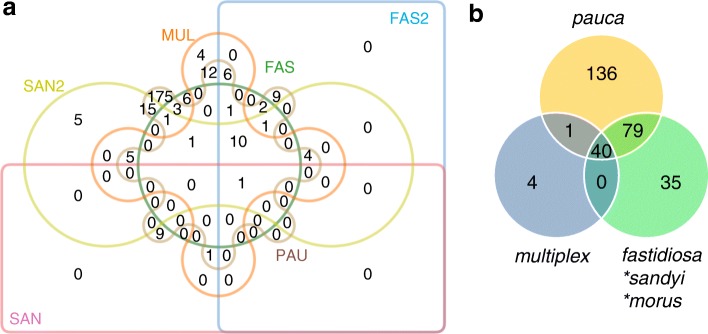
Overlap between gene ontologies differentially represented (over or under) in genes harboring specific k-mers. **a** Relationships between six groups: FAS and FAS2, subsp. *fastidiosa* without or with CFBP 8073, respectively; MUL, subsp. *multiplex*; SAN and SAN2, subsp. *sandyi* and *sandyi*-like, respectively; PAU, subsp. *pauca*. Note: the subsp. *morus* is not indicated on the Venn diagram as it was identified only one GO term, specific to it. **b** Relationships between three groups (*pauca*, *multiplex* and the third one resulting from the grouping of subsp. *fastidiosa*, *sandyi* and *morus*)

Overall, long-mers were identified all along the genomes (Fig. 1), mainly matching coding sequences (71–80% depending on the subspecies), with one to several long-mers in the identified CDS (Table 6A; Additional file 6). Gene set enrichment analysis was performed by comparing the predicted functions associated with these specific CDSs to the overall predicted proteomes. Fischer’s exact test revealed multiple GO terms over- (mostly) or under- (rarely) represented for the seven groups. Overall, only one GO term (catalytic activity) was always found enriched, except for the subspecies *morus*, while 10 other GO terms were found enriched in all groups except in subspecies *morus* and *sandyi* (Table 7; Additional file 7; Fig. 2). Several GO terms were only identified in a single subspecies (Table 8), suggesting that associated mechanisms might be key markers of *X. fastidiosa* subspecies evolution. As for subspecies *pauca* it concerned 175 GO terms, including 20 terms associated with the bacterial cell wall/envelope/plasma membrane and 16 related to nucleotide metabolic/biosynthetic process, especially for purine, as well as 4 terms under-represented dealing with viral or symbiont processes. As for subspecies *fastidiosa* (without CFBP 8073) it concerned six GO terms related to DNA modification and vitamin process. The subspecies *multiplex* specific GO terms deal with metabolic process, catalytic activity and conformation of DNA and organelle organization. The subspecies *morus* had only one ontology enriched, associated with DNA replication.

**Table 6:** Main features related to the specific mers identified in *X. fastidiosa* subspecies

A. *X. fastidiosa* subspecies	FAS^1^	FAS2^1^	SAN^1^	SAN2^1^	MOR^1^	MUL^1^	PAU^1^	
number of mers	2905	1978	9808	5765	3094	4906	11,365	
number of unique mers	2836	1957	9431	5636	2995	4813	11,162	
total mer size (bp)	133,179	85,038	518,683	292,740	142,614	258,228	627,685	
number of mers in CDS	2172	1411	7783	4161	2341	3603	9088	
number of unique CDS	1142	811	2406	1646	1115	1119	1711	
total mer size in CDS (bp)	100,015	60,092	414,736	215,901	108,336	189,973	504,054	
number of mers in intergenic regions	733	567	2025	1604	753	1303	2277	
total mer size in intergenic regions (bp)	33,164	24,946	103,947	76,839	34,278	68,255	123,631	
B. Combination *morus* + other subspecies	MOR-FAS^1^	MOR-FAS2^1^	MOR-SAN^1^	MOR-SAN2^1^	MOR-SAN-SAN2^1^	MOR-SAN-SAN2-FAS2^1^	MOR-MUL^1^	MOR-PAU^1^
number of mers	495	1236	352	371	237	3389	2131	71
number of unique mers	491	1222	347	358	235	3369	2072	71
total mer size (bp)	20,058	53,052	13,123	14,066	8019	136,470	98,450	2377
number of mers in CDS	331	825	258	243	116	2367	1638	58
number of unique CDS	216	430	192	178	103	806	572	41
total mer size in CDS (bp)	13,428	36,147	9816	9275	4249	96,946	76,167	2018
number of mers in intergenic regions	164	411	94	128	121	1022	493	13
total mer size in intergenic regions (bp)	6630	16,905	3307	4791	3770	39,524	22,283	359
C. Within subspecies *pauca*	*pauca* I.1^1^	*pauca* I.2^1^	*pauca* I.3^1^	CFBP 8072^1^	Hib4^1^			
number of mers	1266	2360	3885	4775	4694			
number of unique mers	1238	2323	3644	4663	4596			
total mer size (bp)	59,733	121,367	207,098	283,540	341,563			
number of mers in CDS	1003	1776	3194	3150	3338			
number of unique CDS	486	716	1147	1205	1324			
total mer size in CDS (bp)	47,655	92,528	173,936	194,240	269,888			
number of mers in intergenic regions	263	584	690	1625	1356			
total mer size in intergenic regions (bp)	12,078	28,839	33,162	89,300	71,675			

^1^Composition of the groups:

**MOR** (subsp. *morus*): Mul-MD and Mul0034 (Reference: 2,666,577 bp). **FAS** (subsp. *fastidiosa*): ATCC 35879, DSM 10026, CFBP 7969, CFBP 7970, CFBP 8071, CFBP 8082, CFBP 8351, EB92–1, GB514, M23, Stag’s Leap and Temecula1 (Reference: 2,521,148 bp). **FAS2** (subsp. *fastidiosa*): All the members of the group FAS (with Temecula1 as reference), plus CFBP 8073. **MUL** (subsp. *multiplex*): ATCC 35871, BB01, CFBP 8078, CFBP 8416, CFBP 8417, CFBP 8418, Dixon, Griffin-1, Sy-VA and M12 (Reference: 2,475,130 bp). **SAN** (subsp. *sandyi*): Ann-1 (Reference: 2,780,908 bp). **SAN2** (subsp. *sandyi*-like): CFBP 8356 and CO33 (Reference: 2,416,985 bp). **PAU** (subsp. *pauca*): 32, 3124, 11,399, 6c, CFBP 8072, CoDiRO, COF0324, COF0407, CVC0251, CVC0256, Fb7, Hib4, J1a12, OLS0478, OLS0479, Pr8x, U24D and 9a5c (Reference: 2,731,750 bp). ***pauca*** **I.1** (subsp. *pauca*): U24D, Fb7, CVC0251, CVC0256, J1a12, 11,399, 3124, 32 and 9a5c (Reference: 2,731,750 bp). ***pauca*** **I.2** (subsp. *pauca*): 6c, COF0324 and Pr8x (Reference: 2,666,242 bp). ***pauca*** **I.3** (subsp. *pauca*): COF0407, OLS0478, OLS0479 and CoDiRO (Reference: 2,542,932 bp). **CFBP 8072** (subsp. *pauca*; 2,496,662 bp). **Hib4** (subsp. *pauca;* 2,877,548 bp)

**Table 7:** Main Gene Ontologies (GO) identified as enriched in almost all the subspecies for the CDS harboring specific mers

GO term^1^	Description	FAS^2,3^	FAS2^2,3^	MUL^2,3^	PAU^2,3^	SAN^2,3^	SAN2^2,3^
GO:0003824	catalytic activity	473/368 669/912 1.64e-7/7.48e-11	343/498 468/1113 4.79e-5/4.20e-8	358/308 761/935 0.0082/1.18e-4	672/143 1039/796 4.35e-37/3.55e-40	779/86 1627/311 0.0108/1.34e-5	630/229 1016/670 1.05e-7/4.37e-11
GO:0000166	nucleotide binding	138/96 1004/1184 0.0246/1.46e-4	106/128 705/1483 0.0115/7.90e-5	143/90 976/1153 9.99e-4/8.70e-6	240/32 1471/907 2.40e-18/2.18e-20	–	228/54 1418/845 2.30e-7/3.81e-10
GO:0017076	purine nucleotide binding	109/73 1033/1207 0.0469/3.69e-4	87/95 724/1516 0.0077/3.91e-5	118/70 1001/1173 0.0012/1.25e-5	197/20 1514/919 4.04e-18/4.45e-20	–	181/44 1465/855 2.08e-5/7.77e-8
GO:0032553	ribonucleotide binding	114/74 1028/1206 0.0246/1.33e-4	89/99 722/1512 0.0086/5.12e-5	121/72 998/1171 0.0011/1.12e-5	203/23 1508/916 2.25e-17/2.67e-19	–	186/48 1460/851 5.83e-5/2.89e-7
GO:0032555	purine ribonucleotide binding	108/73 1034/1207 0.0469/4.80e-4	87/94 724/1517 0.0060/2.64e-5	117/70 1002/1173 0.0015/1.70e-5	197/19 1514/920 1.07e-18/8.34e-21	–	181/44 1465/855 2.08e-5/7.77e-8
GO:1901265	nucleoside phosphate binding	138/96 1004/1184 0.0246/1.46e-4	106/128 705/1483 0.0115/7.90e-5	143/90 976/1153 9.99e-4/8.70e-6	240/32 1471/907 2.40e-18/2.18e-20	–	228/54 1418/845 2.30e-7/3.81e-10
GO:0036094	small molecule binding	153/103 989/1177 0.0077/2.11e-5	114/142 697/1469 0.0142/1.10e-4	155/100 964/1143 8.42e-4/5.87e-6	266/33 1445/906 1.49e-21/6.68e-24	–	251/62 1395/837 2.30e-7/2.82e-10
GO:0043168	anion binding	140/87 1002/1193 0.0030/4.98e-6	102/125 709/1486 0.0206/2.07e-4	140/88 979/1155 0.0010/9.88e-6	242/27 1469/912 4.32e-21/2.11e-23	–	226/59 1420/840 7.16e-6/1.77e-8
GO:0097367	carbohydrate derivative binding	117/81 1025/1199 0.0469/4.68e-4	94/104 717/1507 0.0060/2.74e-5	126/73 993/1170 5.43e-4/2.60e-6	209/23 1502/916 2.40e-18/2.31e-20	–	190/50 1456/849 7.27e-5/3.90e-7
GO:0005488	binding	323/277 819/1003 0.0253/1.61e-4	247/353 564/1258 0.0020/5.52e-6	296/245 823/998 0.0078/1.06e-4	547/140 1164/799 1.14e-20/6.54e-23	–	502/187 1144/712 3.03e-5/1.25e-7
GO:0008152	metabolic process	465/411 677/869 0.0055/1.26e-5	355/521 456/1090 4.79e-5/4.37e-8	397/331 722/912 5.94e-4/3.44e-6	723/168 988/771 4.40e-36/5.38e-39	–	644/264 1002/635 1.36e-4/7.91e-7

^1^Complete datasets are provided in Additional file 7

^2^Top line: number of GO-associated CDSs in the list of CDSs harboring specific mers (query) / number of GO-associated CDSs in the CDSs of reference genome that do not harbor specific mers. Middle line: number of non-annotated (no GOs) CDSs in the list of CDSs harboring specific mers (query) / number of non-annotated (no GOs) CDSs in the CDSs of reference genome that do not harbor specific mers. The addition of the four values in each column correspond to the total number of CDS of the reference genome. The addition of the numerator values corresponds to the number of CDS in the query list. The addition of the denominator values corresponds to the number of CDSs of the reference genome that are not in the list of CDSs harboring specific mers. Bottom line: FDR/ *P*-value

^3^Composition of the groups: FAS (subsp. *fastidiosa*, 12): ATCC 35879, DSM 10026, CFBP 7969, CFBP 7970, CFBP 8071, CFBP 8082, CFBP 8351, EB92–1, GB514, M23, Stag’s Leap, Temecula1. FAS2 (subsp. *fastidiosa*, 13): All the members of the group FAS, plus CFBP 8073. MUL (subsp. *multiplex*, 10): ATCC 35871, BB01, CFBP 8078, CFBP 8416, CFBP 8417, CFBP 8418, Dixon, Griffin-1, M12, Sy-VA. SAN (subsp. *sandyi*, 1): Ann-1. SAN2 (subsp. *sandyi*-like, 2): CO33 and CFBP 8356. PAU (subsp. *pauca*, 18): 32, 3124, 11,399, 6c, 9a5c, CFBP 8072, CoDiRO, COF0324, COF0407, CVC0251, CVC0256, Fb7, Hib4, J1a12, OLS0478, OLS0479, Pr8x, U24D

**Table 8:** Selected differentially represented Gene Ontologies of CDS with specific mers in *X*. *fastidiosa* subspecies or subclades

GO term^1^	Description	FDR/*p*-value	Annot. test/ref^2^	Non annot. Test/ref^3^	Enrichment
Specific to subsp. *pauca*: associated with the bacterial cell wall/envelope/plasma membrane; nucleotide metabolic/biosynthetic process, especially for purine					
GO:0000270	peptidoglycan metabolic process	4.40e-36/5.38e-39	723/168	988/771	over
GO:0000902	cell morphogenesis	4.21e-4/2.27e-5	25/0	1686/939	over
GO:0005886	plasma membrane	0.0017/1.15e-4	96/23	1615/916	over
GO:0009252	peptidoglycan biosynthetic process	0.0029/2.08e-4	20/0	1691/939	over
GO:0009273	peptidoglycan-based cell wall biogenesis	0.0029/2.08e-4	20/0	1691/939	over
GO:0009279	cell outer membrane	0.0346/0.0035	19/1	1692/938	over
GO:0009653	anatomical structure morphogenesis	4.21e-4/2.272e-5	25/0	1686/939	over
GO:0016021	integral component of membrane	0.0060/4.49e-4	292/112	1419/827	over
GO:0019867	outer membrane	0.0458/0.0047	25/3	1686/936	over
GO:0030312	external encapsulating structure	0.0088/7.40e-4	22/1	1689/938	over
GO:0031224	intrinsic component of membrane	0.0049/3.68e-4	293/112	1418/827	over
GO:0042546	cell wall biogenesis	0.0029/2.08e-4	20/0	1691/939	over
GO:0044036	cell wall macromolecule metabolic process	0.0012/7.19e-5	23/0	1688/939	over
GO:0044038	cell wall macromolecule biosynthetic process	0.0029/2.08e-4	20/0	1691/939	over
GO:0044425	membrane part	0.0014/8.82eE-5	302/112	1409/827	over
GO:0044462	external encapsulating structure part	0.0346/0.0035	19/1	1692/938	over
GO:0045229	external encapsulating structure organization	0.0023/1.55e-4	26/1	1685/938	over
GO:0048856	anatomical structure development	4.21e-4/2.27e-5	25/0	1686/939	over
GO:0071554	cell wall organization or biogenesis	0.0094/7.93e-4	23/1	1688/938	over
GO:0071555	cell wall organization	0.0346/0.0035	19/1	1692/938	over
GO:0006164	purine nucleotide biosynthetic process	0.0079/6.34e-4	27/2	1684/937	over
GO:0009127	purine nucleoside monophosphate biosynthetic process	0.0088/7.40e-4	22/1	1689/938	over
GO:0009144	purine nucleoside triphosphate metabolic process	0.0035/2.62e-4	28/1	1686/938	over
GO:0009152	purine ribonucleotide biosynthetic process	0.0122/0.0010	26/2	1685/937	over
GO:0009168	purine ribonucleoside monophosphate biosynthetic process	0.0088/7.4042e-4	22/1	1689/938	over
GO:0009205	purine ribonucleoside triphosphate metabolic process	0.0035/2.6218e-4	25/1	1686/938	over
GO:0072522	purine-containing compound biosynthetic process	0.0346/0.0034	18/1	1693/938	over
GO:0072528	pyrimidine-containing compound biosynthetic process	0.0034/2.457e-4	29/2	1682/937	over
GO:0009117	nucleotide metabolic process	0.0012/7.00e-5	64/11	1647/928	over
GO:0009123	nucleoside monophosphate metabolic process	1.59e-5/5.71e-7	49/3	1662/936	over
GO:0009124	nucleoside monophosphate biosynthetic process	0.0014/8.89e-5	32/2	1679/937	over
GO:0009141	nucleoside triphosphate metabolic process	0.0034/2.45e-4	29/2	1682/937	over
GO:0009156	ribonucleoside monophosphate biosynthetic process	6.00e-4/3.25e-5	30/1	1681/938	over
GO:0009165	nucleotide biosynthetic process	0.0106/8.96e-4	43/7	1668/932	over
GO:0009199	ribonucleoside triphosphate metabolic process	0.0023/1.55e-4	26/1	1685/938	over
GO:0009260	ribonucleotide biosynthetic process	6.15e-4/3.36e-5	35/2	1676/937	over
GO:0016032	viral process	0.0213/0.0019	0/6	1711/933	under
GO:0019058	viral life cycle	0.0213/0.0019	0/6	1711/933	under
GO:0019068	virion assembly	0.0213/0.0019	0/6	1711/933	under
GO:0044403	Symbiont process	0.0213/0.0019	0/6	1711/933	under
Specific to subsp. *fastidiosa* (without CFBP 8073; group FAS): associated with DNA modification; vitamin process					
GO:0006304	DNA modification	0.0030/5.64e-6	16/0	1126/1280	over
GO:0006305	DNA alkylation	0.0469/5.31e-4	10/0	1132/1280	over
GO:0006306	DNA methylation	0.0469/5.31e-4	10/0	1132/1280	over
GO:0044728	DNA methylation or demethylation	0.0469/5.31e-4	10/0	1132/1280	over
GO:0009110	vitamin biosynthetic process	0.0469/5.56e-4	18/3	1124/1277	over
GO:0042364	water-soluble vitamin biosynthetic process	0.0469/5.56e-4	18/3	1124/1277	over
Specific to subsp. *multiplex*: associated with metabolic process, catalytic activity and conformation of DNA; organelle organization					
GO:0006259	DNA metabolic process	0.0021/2.52e-5	53/21	1066/1222	over
GO:0071103	DNA conformation change	0.0324/5.80eE-4	13/1	1106/1242	over
GO:0140097	catalytic activity, acting on DNA	0.0018/2.11eE-5	33/8	1086/1235	over
GO:0006996	organelle organization	0.0256/4.47e-4	162	1103/1241	over
Specific to subsp. *morus*: associated with DNA replication					
GO:0006260	DNA replication	0.0485/1.99e-5	31/10	1084/1491	over
Specific to the combination of subsp. *morus* and *multiplex*: associated with amino acid biosynthetic processes; ion binding					
GO:1901607	alpha-amino acid biosynthetic process	0.0390/2.30e-4	26/35	546/2009	over
Specific to the combination of subsp. *morus*, *fastidiosa* (including CFBP 8073), *sandyi*, *sandyi*-like (=clade III): associated with cellular component or protein complex disassembly; signaling; metabolic process; ATP generation; carbohydrates / polysaccharides; nucleoside/nucleotides; peptidyl-proline; response to chemical; tRNA binding; chemotaxis					
GO:0022411	cellular component disassembly	0.0168/8.44e-4	6/0	800/1810	over
GO:0032984	macromolecular complex disassembly	0.0168/8.44e-4	6/0	800/1810	over
GO:0043241	protein complex disassembly	0.0168/8.44e-4	6/0	800/1810	over
GO:0023052	signaling	0.0496/0.0031	18/14	788/1796	over
GO:0007165	signal transduction	0.0496/0.0031	18/14	788/1796	over
GO:0006090	pyruvate metabolic process	0.0086/3.44e-4	13/5	793/1805	over
GO:0006096	glycolytic process	0.0149/5.74e-4	17/10	789/1800	over
GO:0006733	oxidoreduction coenzyme metabolic process	0.0343/0.0018	8/2	798/1808	over
GO:0044264	cellular polysaccharide metabolic process	0.0149/7.01e-4	9/2	797/1808	over
GO:0006757	ATP generation from ADP	0.0078/3.08e-4	11/3	795/1807	over
GO:0016052	carbohydrate catabolic process	0.0359/0.0020	10/4	796/1806	over
GO:0005976	polysaccharide metabolic process	0.0359/0.0020	9/3	797/1807	over
GO:0006165	nucleoside diphosphate phosphorylation	0.0168/8.36e-4	11/4	795/1806	over
GO:0009132	nucleoside diphosphate metabolic process	0.0066/2.55e-4	10/2	796/1808	over
GO:0009135	purine nucleoside diphosphate metabolic process	0.0066/2.55e-4	10/2	796/1808	over
GO:0009179	purine ribonucleoside diphosphate metabolic process	0.0066/2.55e-4	10/2	796/1808	over
GO:0009185	ribonucleoside diphosphate metabolic process	0.0383/0.0022	13/7	793/1803	over
GO:0019362	pyridine nucleotide metabolic process	0.0383/0.0022	13/7	793/1803	over
GO:0046496	nicotinamide nucleotide metabolic process	0.0066/2.58e-4	7/0	799/1810	over
GO:0003755	peptidyl-prolyl cis-trans isomerase activity	0.0066/2.58e-4	7/0	799/1810	over
GO:0000413	protein peptidyl-prolyl isomerization	0.0066/2.58e-4	7/0	799/1810	over
GO:0016859	cis-trans isomerase activity	0.0383/0.0022	13/7	793/1803	over
GO:0018208	peptidyl-proline modification	0.0168/8.36e-4	11/4	795/1806	over
GO:0042221	response to chemical	0.0454/0.00275	5/0	801/1810	over
GO:0000049	tRNA binding	0.0454/0.00275	5/0	801/1810	over
GO:0006935	chemotaxis	0.0454/0.00275	5/0	801/1810	over
GO:0040011	locomotion	0.0168/8.44e-4	6/0	800/1810	over
GO:0042330	taxis	0.0168/8.44e-4	6/0	800/1810	over
Specific to the subclade I.3 from subsp. *pauca*: transport, recombination, organelle part					
GO:0006310	DNA recombination	0.0093/5.91e-4	0/12	1147/1281	under
GO:0006812	cation transport	0.0368/0.0029	2/15	1145/1278	under
GO:0015672	monovalent inorganic cation transport	0.0282/0.0022	1/13	1146/1280	under
GO:0034220	ion transmembrane transport	0.0089/5.56e-4	2/19	1145/1274	under
GO:0098655	cation transmembrane transport	0.0167/0.0012	1/14	1146/1279	under
GO:0098660	inorganic ion transmembrane transport	0.0103/6.72e-4	1/15	1146/1278	under
GO:0098662	inorganic cation transmembrane transport	0.0488/0.0040	1/12	1146/1281	under
GO:0008324	cation transmembrane transporter activity	0.0282/0.0022	1/13	1146/1280	under
GO:0044422	organelle part	0.0167/0.0012	1/14	1146/1279	under
GO:0044446	intracellular organelle part	0.0167/0.0012	1/14	1146/1279	under
Specific to the CFBP 8072 genome from subsp. *pauca*: nucleoside and carboxylic acid biosynthetic processes					
GO:0009142	nucleoside triphosphate biosynthetic process	0.0303/0.0020	0/8	1205/1024	under
GO:0072330	monocarboxylic acid biosynthetic process	0.0158/9.28e-4	0/9	1205/1023	under
Specific to the Hib4 genome from subsp. *pauca*: response to stress, transfer/transport activity, iron-sulfur binding, component assembly/organization					
GO:0006950	response to stress	1.39e-4/1.05e-5	1/17	1323/1047	under
GO:0006979	response to oxidative stress	0.0279/0.0034	0/7	1324/1057	under
GO:0033554	cellular response to stress	0.0153/0.0017	1/11	1323/1053	under
GO:0008565	protein transporter activity	0.0279/0.0034	1/10	1323/1054	under
GO:0009055	electron transfer activity	0.0279/0.0034	0/7	1324/1057	under
GO:0015197	peptide transporter activity	0.0153/0.0017	1/11	1323/1053	under
GO:0016667	oxidoreductase activity, acting on a sulfur group of donors	0.0135/0.0015	0/8	1324/1056	under
GO:0051540	metal cluster binding	0.0055/5.56e-4	2/14	1322/1050	under
GO:0051536	iron-sulfur cluster binding	0.0055/5.56e-4	2/14	1322/1050	under
GO:0051539	4 iron, 4 sulfur cluster binding	0.0279/0.0034	1/10	1323/1054	under
GO:0022607	cellular component assembly	0.0023/2.15e-4	1/13	1323/1051	under
GO:0043933	macromolecular complex subunit organization	0.0066/6.79e-4	0/9	1324/1055	under

^1^Complete datasets are provided in Additional file 7

^2^Annot test/ref.: number of GO-associated CDS in the list of CDS harboring specific mers (query) / number of GO-associated CDS in the reference genome

^3^Non-annot test/ref.: number of non-annotated (no GOs) CDS in the list of CDS harboring specific mers (query) / number of non-annotated (no GOs) CDS in the reference genome

### Reconstruction of the parental origin of the subspecies *morus*

**Fig. 3:**
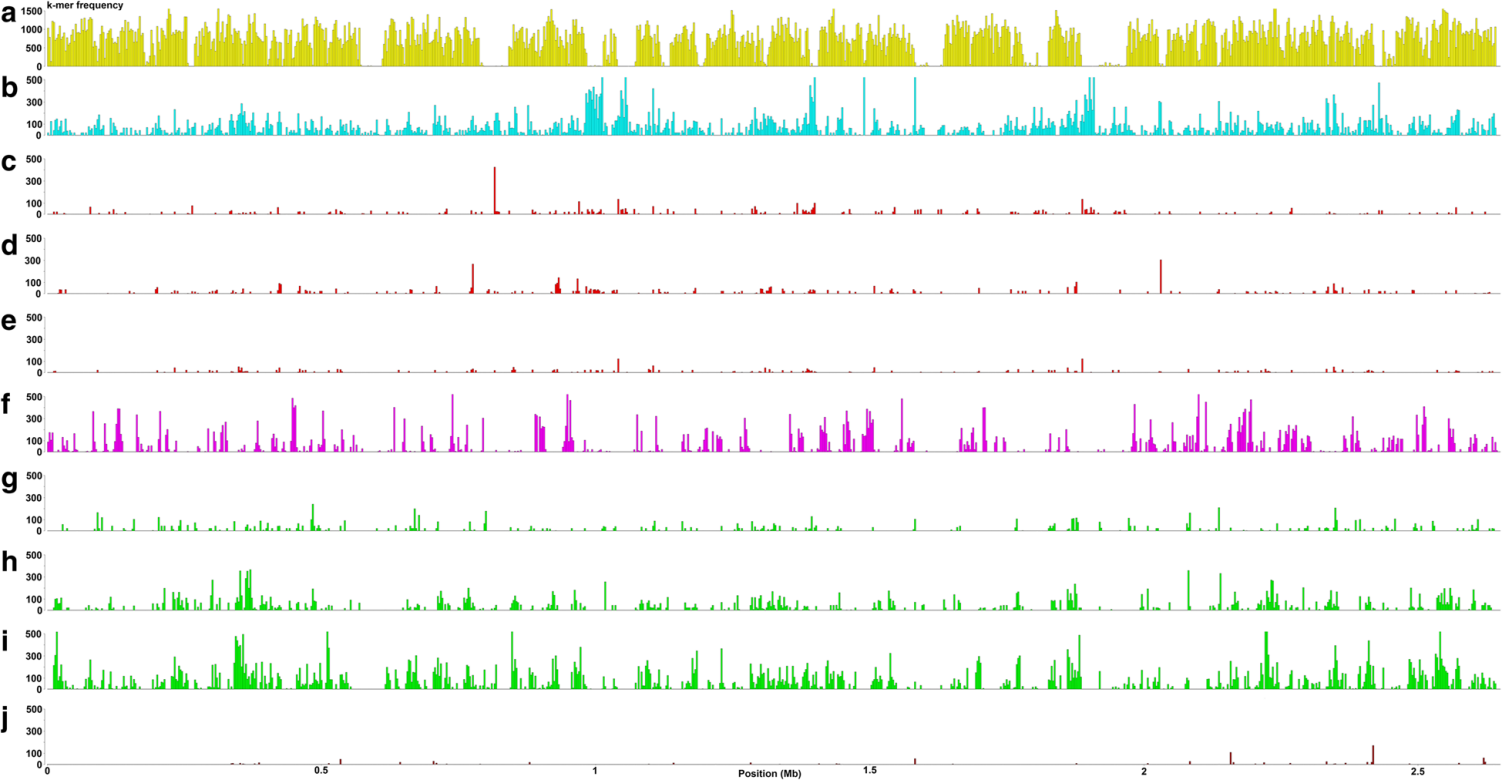
Distribution of k-mers specific to the *X. fastidiosa* subspecies *morus* and others. **a** core k-merome of *X. fastidiosa* species. **b** specific subsp. *morus*. **c, d, e** specific subsp. *morus* + *sandyi* and/or *sandyi*-like. **f** specific subsp. *morus* + *multiplex.* **g, h** specific subsp. *morus* + *fastidiosa* (with/without CFBP 8073 strain). **i** specific subsp. *morus* + *fastidiosa* (with CFBP 8073) + subsp. *sandyi* + subsp. *sandyi*-like*.* **j** specific subsp. *morus* + *pauca*. Frequency of k-mers are mapped onto the genome of reference for subsp. *morus* (Mul0034)

The subspecies *morus* was proposed to group strains pathogenic on *Morus* that derived from largescale intersubspecific homologous recombination events between ancestors from at least subspecies *fastidiosa* and *multiplex*. This assumption is based on the analysis of seven housekeeping genes [16]. We challenged it with whole-genome sequence datasets to further understand the contribution of the subspecies *fastidiosa*, *multiplex* and others in the parenthood of the subspecies *morus*. We used SkIf to identify mers specific of the *morus* group (i.e. two strains, Mul-MD and Mul0034) plus one of each of the other groups (Table 6B; Additional file 6). The highest level of specific mers was found for the combination *morus* x *fastidiosa* x *sandyi*: the highest mer cumulated size represented 5% of the Mul0034 genome in size (which does not mean that all the 5% are unique to Mul0034). The *morus* x *multiplex* (3.6%) and *morus* x *pauca* (< 0.1%) relationships were lower. To illustrate these findings, the specific mers were mapped onto the Mul0034 genome and were found to be distributed all along the sequence (Fig. 3). These results on closest relationships between genomes of subspecies *fastidiosa, sandyi,* and *morus* are coherent with the ANIb values (Additional file 1). Enrichment tests identified shared GO terms for the various combinations indicated (Table 6B). For the combination *morus* x *multiplex,* one GO term linked to the amino acid biosynthetic process was specifically recorded. At the level of *fastidiosa* x *sandyi* x *sandyi*-like x *morus*, 28 GO terms were specifically identified, associated with various processes like cellular component or protein complex disassembly, peptidyl-proline activity and chemotaxis.

A focus on these 28 categories was performed. Due to the redundancy within the GO hierarchical nomenclature, the 28 GOs were reduced to six. All the CDS harboring specific long-mers in Mul0034 genome were retrieved, as well as their closest homologs in the in- (subsp. *fastidiosa, sandyi, sandyi*-like*, morus*) and the out- (subsp. *multiplex* or *pauca*) groups. This corresponded to 26 CDS found each in a single copy in all the 46 *Xf* genomes, indicating that they belong to the *Xf* core genome. For each gene, the sequences were aligned together with the specific long-mers (80 long-mers in total), against the sequence in Mul0034 used as a reference. First, perfect identity in long-mer sequence was conserved among all the genomes of subsp. *fastidiosa, sandyi, sandyi*-like*,* and *morus*. In contrast, SNPs were always found in the alignment of *multiplex* and *pauca* sequences. In comparison with *multiplex*, 46/80 long-mers had only synonymous SNPs and 34 had non-synonymous SNPs in the gene sequences. Considering the 26 CDS, 18 harbored non-synonymous SNPs. In comparison with *pauca*, half long-mers had only synonymous SNPs and half had non-synonymous SNPs in the gene sequences. Considering the 26 CDS, 21 harbored non-synonymous SNPs (Additional file 8).

### Genetic diversity within the subspecies *pauca*

ANIb values clearly showed genetic heterogeneity among strains of the subspecies *pauca*. Three lineages were differentiated: subclade I.1 included seven citrus strains (9a5c, U24D, Fb7, CVC0251, CVC0256, J1a12 and 11,399) and two coffee strains (3124 and 32); subclade I.2 included two coffee (6c and COF0324) and one Prunus (Pr8x) strains, and subclade I.3 included the ST53 strains CoDiRO, COF0407, OLS0478 and OLS0479. Two strains, CFBP 8072 and Hib4 were isolated, as they appeared outside (Additional file 1).

**Fig. 4:**
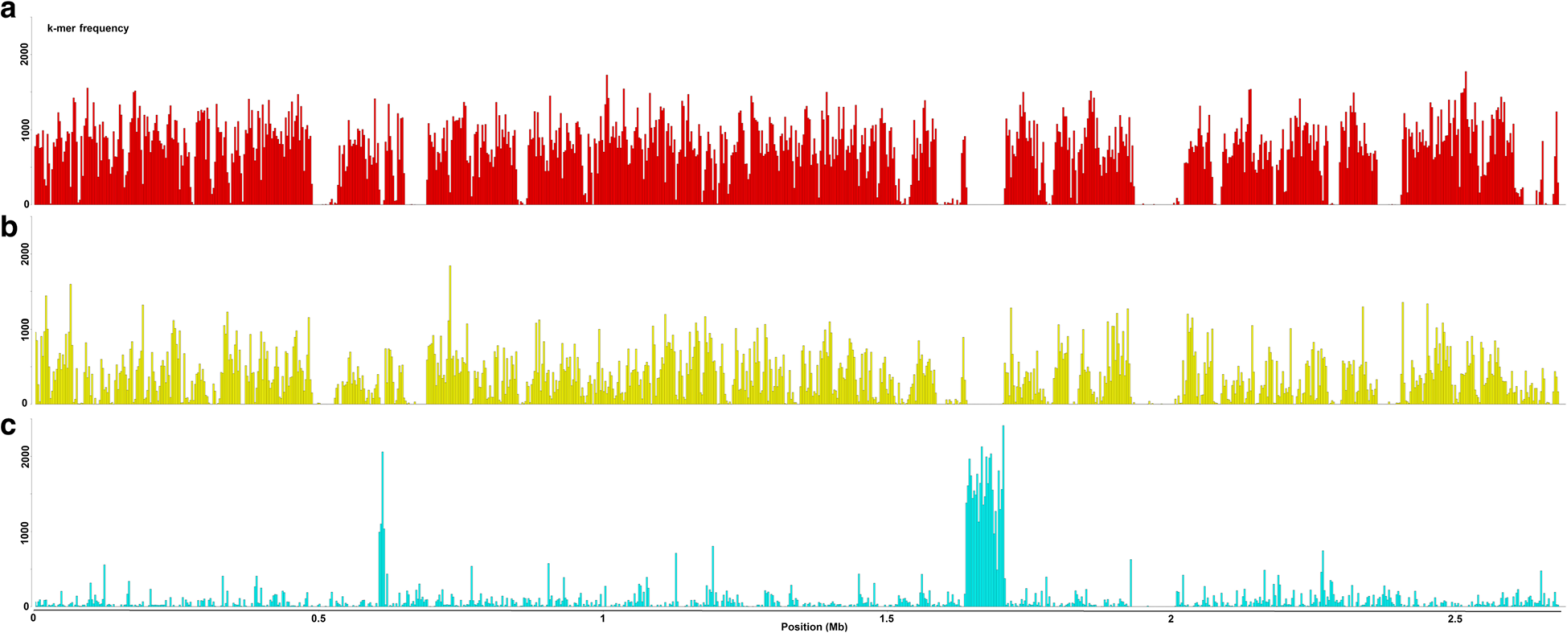
Distribution of k-mers specific to the *X. fastidiosa* subspecies *pauca* and its subclades. **a** core k-merome of *X. fastidiosa* species. **b** specific subsp. *pauca*. **c** specific of subclade I.2, I.3 and strain Hib4 from subsp. *pauca*. Frequency of k-mers are mapped onto the genome of reference for subsp. *morus* (Mul0034)

A search for specific mers within these three subclades (Table 6C, Additional file 6) and the identification of associated GO terms (Table 8; Additional file 7) were performed. At the level of subspecies *pauca* it concerned 175 GO terms, including 16 terms related to nucleotide metabolic/biosynthetic process, especially for purine and 20 terms associated with the bacterial cell wall/envelope/plasma membrane. A focus on these 20 categories was performed. Due to redundancy within the GO hierarchical nomenclature, the 20 GOs were reduced to eight. All the CDS harboring specific long-mers in 9a5c genome were retrieved, as well as their closest homologs in the in- (subsp.*pauca*) and the out- (subsp. *multiplex* or *fastidiosa, sandyi, sandyi*-like*,* and *morus*) groups. This correspond to 105 CDS found each in a single copy in almost all the 46 *Xf* genomes. For each gene, the sequences were aligned together with the specific long-mers (746 k-mers in total), against the sequence in 9a5c used as a reference. First, perfect identity in long-mer sequence was conserved among all the genomes of subsp. *pauca*, except for 6 long-mers with small variants in a few *pauca* strains. In contrast, SNPs were always found in the alignment with *multiplex* and *fastidiosa, sandyi, sandyi*-like*, morus* sequences. In comparison with *multiplex*, 390/746 long-mers had only synonymous SNPs, 354 had non-synonymous SNPs and 2 long-mers were not found. Considering the 105 CDS, 93 harbored non-synonymous SNPs. In comparison with *fastidiosa, sandyi, sandyi*-like*,* and *morus*, 368 long-mers had only synonymous SNPs and 378 had non-synonymous SNPs in the gene sequences. Considering the 105 CDS, 92 harbored non-synonymous SNPs (Additional file 8). The search for enriched GO was also performed at the subclade level. For the subclade I.1, only one term was found (catalytic activity, GO:0003824). In all other studied cases (subclades or individual strains within subsp. *pauca*), all the GO terms identified were under-represented. This might be explained by a recent evolution in these strains/subclades, rendering the corresponding SNPs in these functional gene ontologies less frequent. Two regions harboring specific mers in 13 *pauca* strains (subclades I.2 and I.3, plus Hib4) and absent in the others (subclade I.3 plus CFBP 8072) were identified (Fig. 4). These include genes encoding various enzymes (endonuclease, hydrogenase, hydrolase, integrase/recombinase, methyltransferase, peptidase, polyketide synthase, reductase, terminase, topoisomerase) and genes associated with bacteriophages (Additional file 6). For subclade I.3, 10 GO terms were specific, dealing with transport, recombination, and organelle part. CFBP 8072 has two specific GO terms nucleoside triphosphate biosynthetic process and monocarboxylic acid biosynthetic process. As for the Hib4 genome, 12 terms were found as unique, associated with iron-sulfur complex, or transport activity.

### Identification of chromosome and plasmid specific islands unique in *X. fastidiosa* Hib4

The k-mer approach identified three large genomic regions that were specific to the strain Hib4. A fragment of 34,148 bp (long-mer2422 in Additional file 6) appeared to be chromosomic. It contained 34 genes coding for 12 hypothetical/conserved proteins, 8 conjugal transfer proteins (including TraG), 3 membrane proteins, 2 methyltransferases, and one acriflavine resistance protein B, DEAD/DEAH box helicase, DSBA oxidoreductase, hemolysin secretion protein D, integrating conjugative element protein pill (pfgi-1), lytic transglycosylase, multidrug transporter, RAQPRD family plasmid, and superoxide dismutase (Additional file 6). The screen (blastn) of the Nucleotide collection (nr/nt) database revealed that it shared high identities (> 90%) with sequences of *Cupriavidus* sp., *Comamonas testosterone*, *Pseudomonas aeruginosa*, *Klebsiella pneumoniae* and *Bordetella petrii*, but the largest fragments cover no more than 60% of the *X. fastidiosa* long-mer (Additional file 9).

Two large regions of 32,804 bp (long-mer4538 in Additional file 6) and 16,015 bp (long-mer4596) localized onto the plasmid pXF64-HB. Together with smaller specific long-mers (long-mer4536 to 4596), they accounted for 60,224 bp over the 64,251 bp total size of this plasmid https://www.ncbi.nlm.nih.gov/nuccore/NZ_CP009886.1). It contained 39 genes, including genes coding hypothetical proteins (23), conjugal transfer proteins (7; TraH, I, J, K, N, Q, U, W), and one DNA topoisomerase, endonuclease, helicase, lytic transglycosylase, membrane protein, mobilization protein, protein mobD, relaxase, and TrbA (Additional file 6). The plasmid could have been acquired from a strain of *Paraburkholderia hospita* (93% identity over 86% length of the plasmid), *P. aromaticivorans* (86% identity over 83% length) or even *Burkholderia vietnamiensis* (81% identity over 76% length) or *Xanthomonas euvesicatoria* (80% identity over 72% length) (Additional file 9).

### Robust whole genome-based *X. fastidiosa* clustering with shared k-mers

**Fig. 5:**
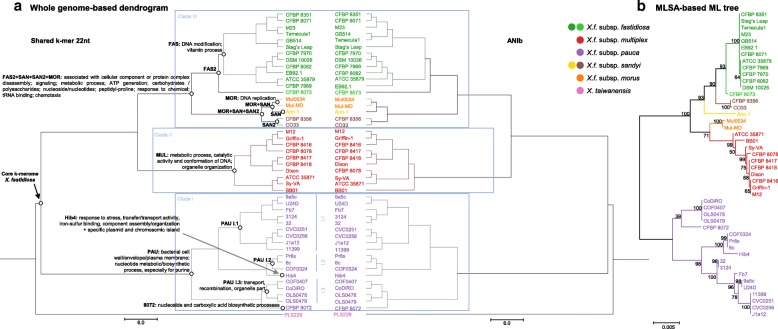
Phylogenetic representation of *X. fastidiosa* using k-mers, ANIb and MLSA schemes. All the representations were constructed using the 46 *X. fastidiosa*, with addition of the *X. taiwanensis* genome sequence. **a** Whole genome-based dendrogram built with distance matrixes obtained after running simka (shared k-mers of 22 nucleotides) or ANIb (1020 nt) algorithms. Some specificities and similarities in enriched gene ontologies or identification of plasmid and chromosomic sequences specific to *X. fastidiosa* are highlighted at nodes or subclades. **b** Maximum-Likelihood (ML) tree constructed with 1000 replicates for bootstrap values using the concatenated sequences (4161 bp) of seven housekeeping genes from a MultiLocus Sequence Analysis (MLSA) scheme. Key features related to *X. fastidiosa* subspecies obtained through the combination of specific k-mer identified and gene ontologies enrichment tests are indicated at nodes

**Fig. 6:**
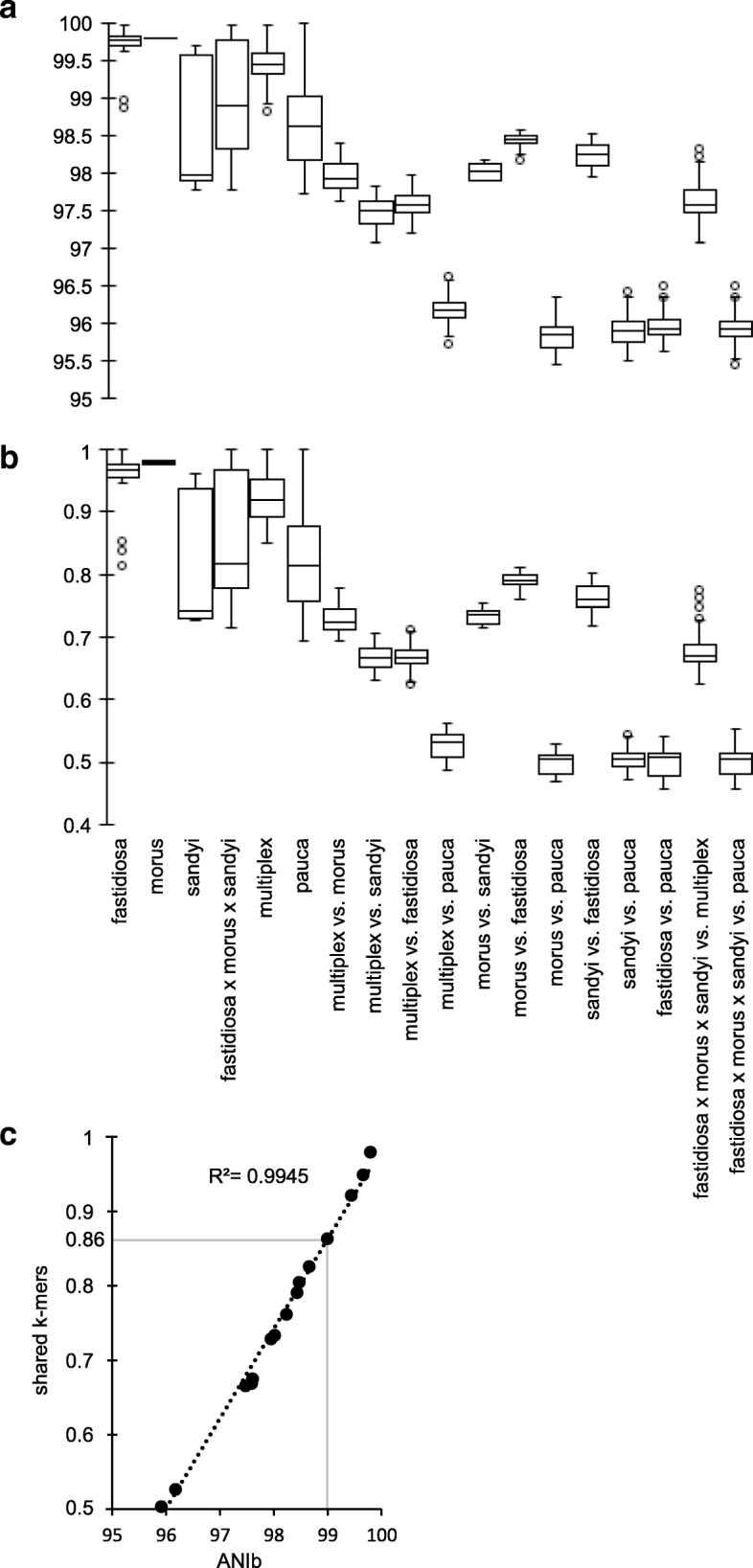
Inter- and intrasubspecies comparisons of ANIb and shared k-mers values. **a** Boxplot of the ANIb values calculated from our genome dataset. **b** Boxplot of the shared k-mer values. **c** Dot plot of the ANIb and shared k-mer mean values. Linear regression and its corresponding r^2^ is indicated. For intrasubpecies comparisons, the number of plotted values corresponds to [(number of genome) ^2^ - number of genome]. For intersubspecies comparisons, it corresponds to [(2 * number of genome subspecies A * number of genome subspecies B)]. Number of genomes: *fastidiosa* (13)*, morus* (2)*, sandyi* (3)*, multiplex* (10)*, pauca* (18)

After looking at specific k-mers in whole genome sequences using SkIf, we employed a complementary approach to draw a robust image of the genetic relationships among individuals, based on shared k-mers. Simka [23] provided a distance matrix that was transformed in a similarity matrix corresponding to the percent of shared k-mers to assess strain relationships (Additional file 10). The k-mer-based dendrogram showed a general distribution into three major clades, represented by the subspecies *pauca* (clade I), *multiplex* (clade II), and the union of subspecies *fastidiosa¸ sandyi* and *morus* (clade III; Fig. 5). It is congruent with the one obtained with ANIb illustrated by with a strong linear regression (r^2^ = 0.9945; Fig. 6). The current clustering of *X. fastidiosa* in five subspecies should be restricted to three subspecies, a proposal that is supported by ANIb and shared k-mers values (99.00% and 0.86, respectively) for clade III (Fig. 6, Additional files 1 and 10). This mostly differ from the view obtained with a MLSA scheme (7 genes) by the repositioning of the subspecies *morus* (Fig. 5). We finally mapped the key points resulting from SkIf (specific k-mers) analysis on the dendrogram (shared k-mers) to illustrate how *X. fastidiosa* genetic diversity can be associated with particular traits (Fig. 5).

## Discussion

While tools based on k-mers are mainly used to improve genome assembly [36, 37], SkIf (https://sourcesup.renater.fr/wiki/skif/) was developed to quickly extract information from genomic datasets from already assembled genomes. It allows to decipher genomic fragments associated with traits shared by a group of sequences of interest. This strategy is applicable to any scientific questions requesting the comparison of user-defined groups of sequences.

Because management of *X. fastidiosa* outbreaks in France depends on the subspecies of *X fastidiosa*, it is of major importance to precisely define these subspecies, understand the robustness of these groupings and their meaning in terms of shared and specific genetic material. In order to detect genomic regions specifically associated with a group of organisms (i.e. a subspecies) we applied SkIf to gain a better understanding of *X. fastidiosa* clusterings in subspecies. This tool was also used to mine large databases as a first step to evaluate worldwide dispersion of *X. fastidiosa* in natural settings.

The phylogeny provided by shared k-mers was highly similar to the one based on ANIb, a reference method for analyzing phylogeny of bacteria [38, 39]. However, phylogenies were much more quickly constructed using shared k-mers than were ANI calculations in JSpecies. Here, k-mers of 22 nt were used while ANIb and TETRA are calculated from k-mers of 1020 and 4 nt, respectively, and ANIm values result from the maximal unique match decomposition of two genomes [40–42].

The current grouping of *X. fastidiosa* in five subspecies is inappropriate and is not supported by genomic data. This is obvious regarding the phylogenies reconstructed using shared-k-mers and ANIb (Fig. 5) and it is coherent with a previous proposal [43]. Three-well demarcated genomic clusters were retrieved in phylogenetic trees reconstructed from 46 genome sequences. *X. fastidiosa* subsp. *fastidiosa* embraced, in addition to the classical subsp*. fastidiosa* strains, the more recently proposed *sandyi* and *morus* subspecies. Mean ANIb values of 99% are found within the former subspecies, while ANIb value with the two later are below 98% (Fig. 6). The two-other subspecies, *multiplex* and *pauca,* were well supported, even if subsp. *pauca* showed a clear divergence between the lineage of strains isolated from citrus and coffee in Brazil vs. the lineage of other strains isolated from coffee from Central America and olive. Indeed, mean ANIb value of 99.44% was calculated within the multiplex subspecies, while ANIb values below than 97% were found with the other two subspecies. Concerning *pauc*a, mean ANIb value of 98.48% was calculated for the 18 genome sequences included in this subspecies while ANIb values of less than 97% were obtained with the two-other species. This mean ANIb value of only 98.66% within *pauca* clearly illustrate the largest diversity found in this subspecies in comparison to the *fastidiosa* and *multiplex* ones. This grouping in a subsp. *fastidiosa* sensu *largo* also matches with an enrichment in 28 GO terms associated with various processes like cellular component or protein complex disassembly, peptidyl-proline activity and chemotaxis. Interestingly, GO-enriched associated CDSs, which harbor k-mers specific to this clade, have homologs in all other *X. fastidiosa* genomes, but most often these homologs present non-synonymous SNPs, avoiding perfect matches with the k-mers. More importantly, this suggests diversity in protein sequences with putative impact on their functions. Referring to the definition of the species and a threshold value of ANI at 95% [39] values calculated here on 46 genomes sequences indicate that *X. fastidiosa* with its diversity forms a unique species.

Grouping the subspecies *morus* within a subspecies *fastidiosa* sensu *largo* is coherent with the results of the analysis made to uncover the origin of the subsp. *morus*. This subspecies was proposed to group strains pathogenic on mulberry trees that derived from recombination events between ancestors of the subspecies *fastidiosa* and *multiplex* [16]. The use of SkIf showed that the cumulated size of the mers uniquely shared within genomes of the clade III (subsp. *morus, fastidiosa, sandyi* and relatives) represents 5% of the Mul0034 genome, while those uniquely shared between subsp. *morus* and *multiplex* count for 3.7%. That showed a closest proximity of *morus* with *fastidiosa* and *sandyi* strains than with *multiplex*. But because some mers are uniquely associated with the two *morus* genomes (Additional file 6), some genetic material of an unknown origin has been introduced in *morus* subspecies genome during evolutionary history.

The evolutionary history of *X. fastidiosa* is also driven by the acquisition of genetic material from heterologous origin. Recently the genome sequence of Hib4 strain was released. This strain presents three large genomic fragments that are unique within *X. fastidiosa*. One of these regions is chromosomic, the two others locate on a large plasmid (~64kbp) that is not found in any other *X. fastidiosa* genome sequence but share strong homology with plasmids from *Burkholderia hospita* DSM17164 [44], *P. aromaticivorans* BN5 [45], *Burkholderia vietnamensis* G4 [46], or *Xanthomonas euvesicatoria* LMG930 [47] strains (Additional file 9). It should be mentioned that so far it is the only case of a plasmid that is not distributed in various strains within *X. fastidiosa* and that originates from a non-*Xylella* strain. Thus, it is tempting to hypothesize on how the acquisition could have occurred, while not easy as these species were isolated from various natural environments including water, soil and plants. Interestingly, *X. euvesicatoria* LMG932 was isolated from *Capsicum frutescensi* in Brazil [48], the country of origin of *X. fastidiosa* Hib4 strain isolated from *Hibiscus fragilis*. Several strains of *Burkholderia vietnamensis* were isolated from Coffee plants in Mexico [49], while other were isolated from a Brazilian cystic fibrosis patient [50]. These findings illustrate the presence of putative plasmid donor either in the country (Brazil) were Hib4 was isolated and on a host (coffee) in a country (Mexico) were *X. fastidiosa* is known to occur. Another particularity of Hib4 is that its harbors two specific regions, only shared with strains of the subsp. pauca subclades I.1 and I.2 (Fig. 4). These genomic regions presumably result from a bacteriophage origin. They could have been acquired specifically by a common ancestor of subclades I.1, I.2 and Hib4 or they could have been lost during evolution, accentuating the degree of divergence with other *pauca* strains.

Microbial collections exchange strains, but mistakes during collection curation cannot be totally excluded, engendering distribution of mislabeled strains, as already shown for *X. fastidiosa* [51]. SkIf proved useful to survey microbial collections for synonyms. Here, following the release of the genome sequence of the *X. fastidiosa* type strain from three origins (CFBP 7970 in the present study, ATCC 35879, DSM 10026), SkIf was used to check the relevance of their synonymy. While not strictly identical due to sequencing and assembly biases, the most striking feature was the putative absence of a large fragment in ATCC 35879, corresponding to a plasmid carrying a complete type IV secretion system [34]. Yet, based on our analysis, it is possible that the plasmid could be present in ATCC 35879, but could have been partially lost during the read filtering and assembly process, or even during strain cultivation. The synonymy between the three strains would therefore be valid. An alternative scenario might be that the plasmid-like sequences have been integrated into the chromosome of ATCC 35879 whilst the plasmid was lost, and in this case, only CFBP 7970 and ATCC 35879 are indeed synonymous. The definite answer will come from an analysis of the raw reads of ATCC 35879 to check for the presence/absence of the plasmid (raw reads are currently not available in SRA) or from a plasmid extraction from the specimen stored at ATCC.

Occurrences of *X. fastidiosa* found in Silva rRNA database were assigned to subspecies that are coherent with sample designation. Specific mers of the genus *Xylella* and of the various subspecies of *X. fastidiosa* were retrieved in the V3-V4 region of the 16S rRNA encoding genes. One use of these tools is to taxonomically assign at the subspecies level the occurrences of *X. fastidiosa* from large database. The sample description and especially the name of the plant species of isolation allow to validate the assignation provided by specific mers. It should however be noticed, that some plant species like almond, olive tree, oleander, coffee tree, or citrus may be host of several *X. fastidiosa* subspecies (www.pubmlst.org/xfastidiosa) [52] and in consequence this a posteriori validation will not always be possible. Long read sequencing will generate more full length 16 s rRNA gene sequences which will facilitate subspecies discrimination. Another tempting use of these tools could be to survey large metagenome database for occurrences of these markers. This is however currently not feasible due to an astonishingly too long time required to download data or incapacity to browse those databases using our markers. Another use will be to design primers and if required probes for PCR detection-identification of *X. fastidiosa* in plant material.

## Conclusions

Skif is a freely available, bioinformatic tool dedicated to the identification of specific mers. Although the results presented here were applied in the context of the emerging plant pathogen *Xylella fastidiosa* in Europe, this software is useful to answer many other questions beyond this scope. It is adapted to mine various group of sequences (gene, protein, genome, metagenome databases) defined by the user to identify specific or shared features. In the context of *X. fastidiosa* it allowed to i) refine the current grouping in subspecies that are not supported by genomic data; ii) trace the origin of the subspecies *morus*, a plasmid from Hib4 strain and the extent of synonymy among specimen representing the same initial strain in microbial collections; and iii) design markers that are specific to each subspecies of *X. fastidiosa*.

## Methods

### Bacterial strains and growth conditions

The seven strains of *X. fastidiosa* (Table 2) used in this study were provided by the French Collection of Plant-Associated Bacteria (CIRM-CFBP; http://www6.inra.fr/cirm_eng/CFBP-Plant-Associated-Bacteria). Strains were grown on B-CYE [53] medium up to 8 weeks at 28 °C. Experiments with *X. fastidiosa* living cells were carried out under quarantine at IRHS, Centre INRA, Beaucouzé, France under the agreement no. 2013119–0002 from the Prefecture de la Région Pays de la Loire, France.

### Genomic DNA extraction

For genome sequencing, bacterial material was harvested on agar plates and suspended in 4.5 ml of sterile, ultrapure water. Genomic DNA was extracted with the NucleoSpin Tissue kit (Macherey-Nagel), following the manufacturer’s recommendations. DNA was recovered in 100 μl of elution buffer (5 mM Tris/HCl, pH 8.5) with final concentration ranging from 3 to 12 μg. Quality and quantity of extracted genomic DNA were checked by depositing an aliquot on agarose gel combined to the use of a nanodrop (Thermo Scientific).

### Library preparation

Genomic DNA solutions were homogenized at 20 ng/μl in 55 μl of resuspension buffer to prepare libraries of sonicated, purified, blunted, and adenylated DNA fragments of 350 bp, following the instructions of the Illumina TruSeq DNA PCR-Free Sample Preparation Guide – Low Sample (LS) Protocol (Catalog #FC-121-9006DOC, Part #150361887 Rev. B, November 2013). Adapters were ligated using the Illumina TruSeq DNA Free PCR LT kit. Libraries were individually quantified and then mixed in a single, equimolar pool (40 nM) also quantified by qPCR following the recommendations of the Library Quantification kit (Kapa Biosystems).

### Genome sequencing, assembling, and annotation

For sequencing, diluted libraries (4 nM) were denatured as described (Illumina Preparing DNA libraries for Sequencing on the MiSeq protocol), resulting in 20pM denatured DNA. The final DNA concentration used for sequencing was 12pM in a 600 μl volume containing 1% of PhiX control. The sample was deposited in a V3 cartridge. The seven *X. fastidiosa* genomes were sequenced with the Illumina MiSeq v3 600 cycles technology at the ANAN plateform, SFR QuaSav, Angers, Fr. Genome assembly was performed using a combination of Velvet [54], SOAPdenovo and SOAPGapCloser [55] assemblers. Structural and functional annotations were conducted with Eugene-PP algorithm [56], using a concatenation of the Swissprot database and the publicly available *X. fastidiosa* 9a5c [57] and Temecula1 [58] genomes.

### Definition of the acronyms

In this study, we used the following definitions for these five key terms: i) mer: a sequence within a nucleotide character string; ii) k-mers: all the possible substrings of length k that are contained in a nucleotide character string; iii) long-mer: a result of the concatenation of overlapping and/or consecutive mers; iv) specific mers: sequences that are exclusively found for members of the group of interest (in-group) while small variants (i.e. with a few indels or SNPs) can be found in some members of the out-groups without being strictly identical; and (v) shared mers: sequences that are found in all the members of different groups of interests or that are common to two individuals in the case of pairwise comparisons.

### Identification of shared or specific k-mers

The percent of shared k-mers between two genome sequences were calculated from the distance matrix built using Simka [23]. Parameters were selected as follow: “-kmer-size 22”, “-abundance-min 1”. SkIf (v1.2) was developed in C ++ and sequence reading was done using Bio++ bpp-seq library [59]. To identify genomic regions that are specific to a group of sequences of interest, SkIf construct an abundance matrix of all mers of sequences. This matrix is used to identify the mers present in all the sequences of the group of interest (in-group) and absent in all the other sequences. Parameters were selected as follow: “-k 22”, “-a dna”, “-g = in-group list”. Then, it maps the specific mers of the in-group to the reference genome sequence of the group and provides their precise locations. By comparing mer length and the positions of the various occurrences, SkIf concatenates the overlapping mers into long-mer using the script “getLongestKmersNC.pl” (option: -k 22; available with Skif). A list of located mers or long-mers specific to the group of interest was hence obtained. Finally, we developed a wrapper for accessing this process in a user-friendly Galaxy tool (https://iris.angers.inra.fr/galaxypub-cfbp). Hence, SkIf allows to extract all specific mers of a dataset. The optimal size of the mer was fixed to 22 nt to optimize the ratio of in-group to out-group specific sequences, after a comparison of a range from 18 to 26 nt was done (data not shown).

### Analyses of genome and nucleotide sequences and phylogeny

For the analyses of the seven housekeeping genes used in MLSA-MLST scheme designed for *X. fastidiosa* (https://pubmlst.org/xfastidiosa/info/primers.shtml) and the 16S rRNA gene and the synonymy of SNPs, nucleotide sequences were aligned using the Geneious suite, with the default parameters of the ‘MUSCLE Alignment’ and the ‘Map to Reference’ options [60]. Maximum-Likelihood (ML) tree was constructed with 1000 replicates for bootstrap values using the concatenated sequences (4161 bp) of seven housekeeping genes from the MLSA scheme. ANIb values were calculated using Pyani [30]. Similarity matrix (based on ANIb or shared k-mers) were transformed into distance matrix (1-ANIbs*100 or 1-shared k-mers*100) in the dist format of R using as.dist and clustered using Ward’s method [61] for hierarchical clustering. Conversion of the distance matrix into dendrograms relied on as.phylo function from R ape package [62]. Blastn (v2.8.0+) analyses were run against the nucleotide collection (nt; 46,977,437 sequences) [63].

### Enrichment tests and Venn diagram representations

Enrichment analyses with a Fisher’s Exact Test were performed with Blast2GO v4.1 [64], using the Gene Ontology functional annotations to compare gene lists carrying specific k-mers against all the gene of the reference genomes to identify statistically significant enrichment in biological processes or molecular functions. Venn diagrams were built using jvenn (http://jvenn.toulouse.inra.fr/app/index.html [65].

### Development of a galaxy-based website and user guidelines

The SkIf pipeline is free for use online (https://iris.angers.inra.fr/galaxypub-cfbp). Required input files are a zip file with all the fasta files for the in-group genome sequences; a zip file with all the fasta files for the outgroup genome sequences; the length of the k, and the identifier of the reference genome sequence from the in-group. Output files are text files with the list of the k-mers and long-mers specific to the in-group if existing. A wiki page describing SkIf is accessible at https://sourcesup.renater.fr/wiki/skif.

### Mining of 16S rRNA database

For the genus analysis, the in-group included the 74 *Xylella* 16S rRNA copies and the out-group included all the small subunit rRNA gene (SSU) sequences from the Silva database (https://www.arb-silva.de/; release 128) [66] other than *Xylella*-tagged. For the species analysis, *X. taiwanensis* PLS 229 strain was included in the out-group. All sequences affiliated to *X. fastidiosa* were included in the in-group, while all the other, non-*X. fastidiosa*, were included in the out-group. Ambiguous sequences (e.g. double assignation to *Xylella* and *Xanthomonas*) were excluded. SkIf software was used as described above to identify specific k-mers (−k 22) in the in-group and to concatenate consecutive ones in long-mers.

## Additional files

Additional file 1: Pairwise comparison of 47 *Xylella* sp. genomes using average nucleotide identity based on blast (ANIb). (DOCX 63 kb)

Additional file 2: K-mers resulted from the comparison of synonymous strains CFBP 7970, DSM 10026 and ATCC 35879. (ZIP 45 kb)

Additional file 3: Blast analysis of the known *X. fastidiosa* plasmid sequences against the genome sequence of ATCC 35879. (DOCX 30 kb)

Additional file 4: Raw data of analyses of the 16S rRNA gene repertoire in *Xylella. (ZIP 12 kb)*

Additional file 5: *X. fastidiosa* 16S rRNA sequences from Silva database carrying the five long-mers and taxonomically assigned to a subspecies with the SNP-based code. (DOCX 21 kb)

Additional file 6: Raw data of the k-mers identified to be specific of the different *X. fastidiosa* subspecies or combinations of several subspecies. (ZIP 3012 kb)

Additional file 7: Raw data of the gene ontologies enrichments tests with Blast2GO. (ZIP 22422 kb)

Additional file 8: Analysis of (non-)synonymous SNPs in homologs to CDS harboring specific k-mers for selected enriched GOs. (ZIP 549 kb)

Additional file 9: Blast analysis of the three large fragments specific to *X. fastidiosa* subsp. *pauca* Hib4 strain. (DOCX 34 kb)

Additional file 10: Pairwise comparison of 47 *Xylella* sp. genomes using the occurrence of shared k-mers of length 22 bp. (DOCX 68 kb)

## Abbreviations

ANIb: Average nucleotide identities using blast
ANIm: Average nucleotide identities using MUMmer
CDS: Coding sequence
GO: Gene ontology
ICE: Integrative and conjugative elements
rRNA: Ribosomal RNA
SkIf: Specific k-mers Identification
SNP: Single nucleotide polymorphism
TETRA: Tetranucleotide frequency correlation coefficients
