# Supplementary material for: Identification of genetic relationships and subspecies signatures in Xylella fastidiosa
Source: BMC Genomics. 2019 Mar 25;20:239. doi: 10.1186/s12864-019-5565-9 (PMC6434890; doi:10.1186/s12864-019-5565-9)
Supplement: Supplementary file 1 — Pairwise comparison of 47 Xylella sp. genomes using average nucleotide identity based on blast (ANIb). (DOCX 63 kb) [file 12864_2019_5565_MOESM1_ESM.docx]

**Additional File 1.** Pairwise comparison of 47 *Xylella* sp. genomes using average nucleotide identity based on blast (ANIb).

Values in colors are as follow: red for *X. fastidiosa* subsp. *multiplex*; orange for *X. fastidiosa* subsp. *morus*; pink/brown for *X. fastidiosa* subsp. *sandyi*; light/dark green for *X. fastidiosa* subsp. *fastidiosa*; purple for *X. fastidiosa* subsp. *pauca*.
