# Supplementary material for: Identification of genetic relationships and subspecies signatures in Xylella fastidiosa
Source: BMC Genomics. 2019 Mar 25;20:239. doi: 10.1186/s12864-019-5565-9 (PMC6434890; doi:10.1186/s12864-019-5565-9)
Supplement: Supplementary file 3 — Blast analysis of the known X. fastidiosa plasmid sequences against the genome sequence of ATCC 35879. (DOCX 30 kb) [file 12864_2019_5565_MOESM3_ESM.docx]

**Additional File 3.** Blast analysis of the known *X. fastidiosa* plasmid sequences against the genome sequence of ATCC 35879.

| **Plasmid**  **(size; strain)** | **Query**  **id** | **Query**  **start** | **Query**  **end** | **Subject**  **id** | **Subject**  **start** | **Subject**  **end** | **% identity** | **alignment length** | **Total length** | **Mismatches**  **/gap** | **e-value** | **bit score** |
| --- | --- | --- | --- | --- | --- | --- | --- | --- | --- | --- | --- | --- |
| pXF-RIV5 | JX548317.1 | 32033 | 33504 | JQAP01000014.1 | 71462 | 69982 | 96.29 | 1,481 | 6,032 | 46/4 | 0.0 | 2490 |
| (38,297bp; RIV5) | JX548317.1 | 8347 | 9416 | JQAP01000014.1 | 259459 | 260528 | 96.45 | 1,071 |  | 36/2 | 0.0 | 1820 |
|  | JX548317.1 | 14504 | 15700 | JQAP01000004.1 | 359551 | 360756 | 97.51 | 1,206 |  | 21/2 | 0.0 | 2143 |
|  | JX548317.1 | 11061 | 11610 | JQAP01000004.1 | 358889 | 359446 | 94.44 | 558 |  | 23/4 | 0.0 | 852 |
|  | JX548317.1 | 20397 | 21407 | JQAP01000012.1 | 26193 | 25183 | 98.81 | 1,011 |  | 12/0 | 0.0 | 1909 |
|  | JX548317.1 | 19019 | 19478 | JQAP01000008.1 | 21733 | 22192 | 96.96 | 460 |  | 14/0 | 0.0 | 775 |
|  | JX548317.1 | 19853 | 20097 | JQAP01000008.1 | 22193 | 22437 | 98.78 | 245 |  | 3/0 | 6,00E-129 | 462 |
| pXFAS01 | CP001012.1 | 32033 | 33504 | JQAP01000014.1 | 71462 | 69982 | 96.29 | 1,481 | 6,032 | 46/4 | 0.0 | 2490 |
| (38,297bp; M23) | CP001012.1 | 8347 | 9416 | JQAP01000014.1 | 259459 | 260528 | 96.45 | 1,071 |  | 36/2 | 0.0 | 1820 |
|  | CP001012.1 | 14504 | 15700 | JQAP01000004.1 | 359551 | 360756 | 97.51 | 1,206 |  | 21/2 | 0.0 | 2143 |
|  | CP001012.1 | 11061 | 11610 | JQAP01000004.1 | 358889 | 359446 | 94.44 | 558 |  | 23/4 | 0.0 | 852 |
|  | CP001012.1 | 20397 | 21407 | JQAP01000012.1 | 26193 | 25183 | 98.81 | 1,011 |  | 12/0 | 0.0 | 1909 |
|  | CP001012.1 | 19019 | 19478 | JQAP01000008.1 | 21733 | 22192 | 96.96 | 460 |  | 14/0 | 0.0 | 775 |
|  | CP001012.1 | 19853 | 20097 | JQAP01000008.1 | 22193 | 22437 | 98.78 | 245 |  | 3/0 | 6,00E-129 | 462 |
| pXF-De_Donno | CP020871.1 | 16810 | 18003 | JQAP01000004.1 | 360756 | 359554 | 97.92 | 1,203 | 4,229 | 16/2 | 0.0 | 2177 |
| (35,273bp; De Donno) | CP020871.1 | 11475 | 12485 | JQAP01000012.1 | 25183 | 26193 | 98.91 | 1,011 |  | 11/0 | 0.0 | 1917 |
|  | CP020871.1 | 21876 | 22925 | JQAP01000014.1 | 260515 | 259466 | 97.52 | 1,050 |  | 26/0 | 0.0 | 1875 |
|  | CP020871.1 | 22995 | 23253 | JQAP01000014.1 | 45494 | 45236 | 99.23 | 259 |  | 2/0 | 1,00E-139 | 498 |
|  | CP020871.1 | 13403 | 13863 | JQAP01000008.1 | 22193 | 21733 | 96.10 | 461 |  | 18/0 | 0.0 | 771 |
|  | CP020871.1 | 12785 | 13029 | JQAP01000008.1 | 22437 | 22193 | 99.59 | 245 |  | 1/0 | 1,00E-133 | 478 |
| unnamed | CM003178.1 | 16835 | 18028 | JQAP01000004.1 | 360756 | 359554 | 97.92 | 1,203 | 4,229 | 16/2 | 0.0 | 2177 |
| (35,318bp; CoDiRO) | CM003178.1 | 11500 | 12510 | JQAP01000012.1 | 25183 | 26193 | 98.91 | 1,011 |  | 11/0 | 0.0 | 1917 |
|  | CM003178.1 | 21901 | 22950 | JQAP01000014.1 | 260515 | 259466 | 97.52 | 1,050 |  | 26/0 | 0.0 | 1875 |
|  | CM003178.1 | 23020 | 23278 | JQAP01000014.1 | 45494 | 45236 | 99.23 | 259 |  | 2/0 | 1,00E-139 | 498 |
|  | CM003178.1 | 13428 | 13888 | JQAP01000008.1 | 22193 | 21733 | 96.10 | 461 |  | 18/0 | 0.0 | 771 |
|  | CM003178.1 | 12810 | 13054 | JQAP01000008.1 | 22437 | 22193 | 99.59 | 245 |  | 1/0 | 1,00E-133 | 478 |
| pXF-P4.CVC0251 | CM003756.1 | 13992 | 15185 | JQAP01000004.1 | 359551 | 360753 | 97.51 | 1,203 | 4,229 | 21/2 | 0.0 | 2137 |
| (30,973bp; CVC0251) | CM003756.1 | 19129 | 20138 | JQAP01000012.1 | 26193 | 25183 | 98.52 | 1,011 |  | 14/1 | 0.0 | 1883 |
|  | CM003756.1 | 29963 | 30973 | JQAP01000014.1 | 259459 | 260463 | 95.75 | 1,011 |  | 37/2 | 0.0 | 1618 |
|  | CM003756.1 | 13074 | 13371 | JQAP01000014.1 | 47154 | 47451 | 97.32 | 298 |  | 8/0 | 1,00E-148 | 527 |
|  | CM003756.1 | 17751 | 18211 | JQAP01000008.1 | 21733 | 22193 | 96.96 | 461 |  | 14/0 | 0.0 | 803 |
|  | CM003756.1 | 18585 | 18829 | JQAP01000008.1 | 22193 | 22437 | 98.78 | 245 |  | 3/0 | 5,00E-129 | 462 |
| pXF-P4.OLS0479 | CM003763.1 | 4448 | 5641 | JQAP01000004.1 | 359554 | 360756 | 97.84 | 1,203 | 4,229 | 17/2 | 0.0 | 2169 |
| (35,678bp; OLS0479) | CM003763.1 | 9966 | 10976 | JQAP01000012.1 | 26193 | 25183 | 98.91 | 1,011 |  | 11/0 | 0.0 | 1917 |
|  | CM003763.1 | 34577 | 35626 | JQAP01000014.1 | 259466 | 260515 | 97.52 | 1,050 |  | 26/0 | 0.0 | 1875 |
|  | CM003763.1 | 34249 | 34507 | JQAP01000014.1 | 45236 | 45494 | 99.23 | 259 |  | 2/0 | 1,00E-139 | 498 |
|  | CM003763.1 | 8588 | 9048 | JQAP01000008.1 | 21733 | 22193 | 96.10 | 461 |  | 18/0 | 0.0 | 771 |
|  | CM003763.1 | 9422 | 9666 | JQAP01000008.1 | 22193 | 22437 | 99.59 | 245 |  | 1/0 | 1,00E-133 | 478 |
| pXF-P4.CVC0256 | CM003750.1 | 15035 | 16228 | JQAP01000004.1 | 359551 | 360753 | 97.51 | 1,203 | 4,140 | 21/2 | 0.0 | 2137 |
| (30,865bp; CVC0256) | CM003750.1 | 20172 | 21181 | JQAP01000012.1 | 26193 | 25183 | 98.52 | 1,011 |  | 14/1 | 0.0 | 1883 |
|  | CM003750.1 | 312 | 1233 | JQAP01000014.1 | 259605 | 260526 | 96.10 | 922 |  | 36/0 | 0.0 | 1542 |
|  | CM003750.1 | 14117 | 14414 | JQAP01000014.1 | 47154 | 47451 | 97.32 | 298 |  | 8/0 | 1,00E-148 | 527 |
|  | CM003750.1 | 18794 | 19254 | JQAP01000008.1 | 21733 | 22193 | 96.96 | 461 |  | 14/0 | 0.0 | 803 |
|  | CM003750.1 | 19628 | 19872 | JQAP01000008.1 | 22193 | 22437 | 98.78 | 245 |  | 3/0 | 5,00E-129 | 462 |
| pXF-P4.OLS0478 | CM003753.1 | 20712 | 21905 | JQAP01000004.1 | 360756 | 359554 | 98.17 | 1,203 | 3,998 | 13/3 | 0.0 | 2200 |
| (40,515bp; OLS0478) | CM003753.1 | 31084 | 32162 | JQAP01000014.1 | 260544 | 259466 | 98.52 | 1,079 |  | 16/0 | 0.0 | 2012 |
|  | CM003753.1 | 15377 | 16387 | JQAP01000012.1 | 25183 | 26193 | 99.60 | 1,011 |  | 4/0 | 0.0 | 1972 |
|  | CM003753.1 | 17306 | 17765 | JQAP01000008.1 | 22192 | 21733 | 99.78 | 460 |  | 1/0 | 0.0 | 904 |
|  | CM003753.1 | 16687 | 16931 | JQAP01000008.1 | 22437 | 22193 | 99.59 | 245 |  | 1/0 | 1,00E-133 | 478 |
| unnamed | CP014330.1 | 17637 | 18829 | JQAP01000004.1 | 359554 | 360756 | 97.42 | 1,203 | 3,915 | 21/3 | 0.0 | 2129 |
| (39,400bp; Fb7) | CP014330.1 | 23154 | 24164 | JQAP01000012.1 | 26193 | 25183 | 97.82 | 1,011 |  | 22/0 | 0.0 | 1830 |
|  | CP014330.1 | 8389 | 9383 | JQAP01000014.1 | 259512 | 260500 | 95.58 | 995 |  | 38/2 | 0.0 | 1618 |
|  | CP014330.1 | 21776 | 22236 | JQAP01000008.1 | 21733 | 22193 | 97.18 | 461 |  | 13/0 | 0.0 | 811 |
|  | CP014330.1 | 22610 | 22854 | JQAP01000008.1 | 22193 | 22437 | 98.78 | 245 |  | 3/0 | 7,00E-129 | 462 |
| pXF-P4.COF0407 | CM003744.1 | 1 | 1086 | JQAP01000004.1 | 359671 | 360756 | 98.71 | 1,086 | 3,881 | 14/0 | 0.0 | 2042 |
| (33,048bp; COF0407) | CM003744.1 | 30057 | 31135 | JQAP01000014.1 | 259466 | 260544 | 98.52 | 1,079 |  | 16/0 | 0.0 | 2012 |
|  | CM003744.1 | 5411 | 6421 | JQAP01000012.1 | 26193 | 25183 | 99.60 | 1,011 |  | 4/0 | 0.0 | 1972 |
|  | CM003744.1 | 4033 | 4492 | JQAP01000008.1 | 21733 | 22192 | 99.78 | 460 |  | 1/0 | 0.0 | 904 |
|  | CM003744.1 | 4867 | 5111 | JQAP01000008.1 | 22193 | 22437 | 99.59 | 245 |  | 1/0 | 9,00E-134 | 478 |
| unnamed1 | CP006697.1 | 15420 | 16019 | JQAP01000014.1 | 46522 | 47122 | 93.52 | 602 | 1,436 | 36/2 | 0.0 | 880 |
| (30,305bp; Ann-1) | CP006697.1 | 14429 | 14805 | JQAP01000014.1 | 45585 | 45964 | 91.10 | 382 |  | 27/5 | 2,00E-134 | 480 |
|  | CP006697.1 | 3849 | 4074 | JQAP01000008.1 | 21415 | 21640 | 99.56 | 226 |  | 1/0 | 2,00E-122 | 440 |
|  | CP006697.1 | 8999 | 9224 | JQAP01000008.1 | 21640 | 21415 | 99.56 | 226 |  | 1/0 | 2,00E-122 | 440 |
| pXF-BHR.CVC0256 (51,156bp; CVC0256) | CM003748.1 | 43823 | 44870 | JQAP01000014.1 | 260500 | 259459 | 95.42 | 1,048 | 1,048 | 42/2 | 0.0 | 1651 |
| pXF51ud  (51156bp; U24D) | CP009791.1 | 27240 | 28287 | JQAP01000014.1 | 260500 | 259459 | 95.42 | 1,048 | 1,048 | 42/2 | 0.0 | 1651 |
| pXF51  (51,158bp; 9a5c) | AE003851.1 | 27242 | 28289 | JQAP01000014.1 | 260500 | 259459 | 95.42 | 1,048 | 1,048 | 42/2 | 0.0 | 1651 |
| pXF51-J1  (51,180bp; J1a12) | CP009825.1 | 27248 | 28163 | JQAP01000014.1 | 260500 | 259585 | 96.07 | 916 | 916 | 36/0 | 0.0 | 1530 |
| pXF-BHR.CVC0251 (51,175bp; CVC0251) | CM003754.1 | 43073 | 43968 | JQAP01000014.1 | 260500 | 259605 | 95.65 | 896 | 896 | 39/0 | 0.0 | 1467 |
| pXF-RIV11  (25,105bp; RIV11) | GU938457.1 | 17691 | 18285 | JQAP01000014.1 | 260394 | 260992 | 95.16 | 599 | 599 | 25/2 | 0.0 | 920 |
| pXF-RIV19  (24,372bp; RIV19) | GU938459.1 | 16958 | 17552 | JQAP01000014.1 | 260394 | 260992 | 95.16 | 599 | 599 | 25/2 | 0.0 | 920 |
| unnamed2  (24,391bp; Mul0034) | CP006739.1 | 7755 | 8349 | JQAP01000014.1 | 260992 | 260394 | 94.99 | 599 | 599 | 26/2 | 0.0 | 912 |
| unnamed  (26,180 bp; GB514) | CP002166.1 | 13408 | 14001 | JQAP01000014.1 | 260394 | 260992 | 95.16 | 599 | 599 | 24/3 | 0.0 | 951 |
| pXF-P1.COF0407 (26,571bp; COF0407) | CM003743.1 | 24404 | 24629 | JQAP01000008.1 | 21640 | 21415 | 98.67 | 226 | 226 | 3/0 | 1,00E-117 | 424 |
| pXF-P1.OLS0478 (31,618bp; OLS0478) | CM003752.1 | 1443 | 1668 | JQAP01000008.1 | 21415 | 21640 | 98.67 | 226 | 226 | 3/0 | 1,00E-117 | 424 |
| pXF-P1.OLS0479 (31,618bp; OLS0479) | CM003762.1 | 1443 | 1668 | JQAP01000008.1 | 21415 | 21640 | 98.67 | 226 | 226 | 3/0 | 1,00E-117 | 424 |

No matches were found in ATCC 35879 genome sequence for the following *X. fastidiosa* plasmids (size; strain; accession ID):

pXF-PS.COF0407 (4,520bp; COF0407; CM003745.1), pXF-RC.COF0407 (1,295bp; COF0407; CM003746.1), pXF-P1.CVC0256 (27,109bp; CVC0256; CM003749.1), pXF-PS.CVC0256 (5,825bp; CVC0256; CM003751.1), pXF-P1.CVC0251 (27,016bp; CVC0251; CM003755.1), pXF-PS.CVC0251 (5,825bp; CVC0251; CM003757.1), pXF-BHR-COF0324 (36,209bp; COF0324; CM003758.1), pXF-P1.COF0324 (26,508bp; COF0324; CM003759.1), pXF-PC_COF0324 (5,557bp; COF0324; CM003760.1), pXF-RC.COF0324 (1,285bp; COF0324; CM003761.1), pXF-PS.OLS0479 (5,926bp; OLS0479; CM003764.1), pXF-RC.OLS0479 (1,305bp; OLS0479; CM003765.1), pXF868 (1,296bp; ATCC 35868; U71220.1), pUCLAb (1,293bp; UCLA; DQ063225.1), pUCLAa (1,298bp; UCLA; DQ063224.1), pUCLAc (1,284bp; UCLA; DQ063226.1), pXF64-HB (64,251bp; Hib4; CP009886.1), pXF27-J1 (27,268bp; J1a12; CP009824.1), pXF39 (39,580bp; Pr8x; CP009827.1), pXF6c (39,572bp; 6c; CM007617.1), pXF1.3 (1,286bp; 9a5c; AE003850.3), pXFPD1.3 (1,346bp; Temecula1; AE009443.1), pXF51 (45,356bp; 11399; CM004499.1).
