# Supplementary material for: Identification of genetic relationships and subspecies signatures in Xylella fastidiosa
Source: BMC Genomics. 2019 Mar 25;20:239. doi: 10.1186/s12864-019-5565-9 (PMC6434890; doi:10.1186/s12864-019-5565-9)
Supplement: Supplementary file 5 — X. fastidiosa 16S rRNA sequences from Silva database carrying the five long-mers and taxonomically assigned to a subspecies with the SNP-based code. (DOCX 21 kb) [file 12864_2019_5565_MOESM5_ESM.docx]

**Additional File 5.** *X. fastidiosa* 16S rRNA sequences from Silva database carrying the five long-mers and taxonomically assigned to a subspecies with the SNP-based code.

| *X. fast.* subsp. | Isolate^a^ | Accession | SNP code corresp.^b^ | SNP code var.^c^ | Country  (Year)^d^ | Host of isolation^d^ |
| --- | --- | --- | --- | --- | --- | --- |
| *fastidiosa* | KSI27 | KC113177.1 | 100% | - | Iran (2012) | Dust |
| (n=11) | VvIIc2  Alf34  Alf-SNI  Geo1  Kings  M23  MM1  Mus1  SP3  TO1 | EF433931.1  EU526729.1  EU526730.1  EU526738.1  EU526739.1  EU526741.1  KF870458.1  EU526742.1  KF870459.1  KF870460.1 | 87.5%  75%  75%  75%  75%  75%  75%  75%  75%  75% | C1340T  Incomplete  Incomplete  Incomplete  Incomplete  Incomplete  Incomplete  Incomplete  Incomplete  Incomplete | Costa Rica (<2007)  USA (<2008)  USA (<2008)  USA (<2008)  USA (<2008)  USA (<2008)  Taiwan (<2013)  USA (<2008)  Taiwan (<2013)  Taiwan (<2013) | Grapevine  Alfalfa  Alfalfa  Grapevine  Grapevine  Grapevine  *Mikania* *micrantha*  Grapevine  *Solanum* *pseudocapsicum* Linn.  *Trema* *orientalis* (L.) Blume. |
| *morus* (n=1) | MB1-uncult | DQ021532.1 | 100% | - | USA (<2005) | Mulberry |
| *multiplex*  (n=32) | LH18-uncult  PO1-uncult  RO1-uncult | DQ022859.1  DQ021535.1  DQ021540.1 | 100%  100%  100% | -  -  - | USA (2002) ^e^  USA (2002) ^e^  USA (2002) ^e^ | *Graphocephala* *versuta*  Pin oak  Red oak |
|  | 87H-uncult  109H-uncult  108H-uncult  11H-uncult  40H-uncult  86H-uncult  BB1  BB2  BB3  BB4  ELM1  EO  EOS  GA18a  GA18b  GA19a  GA19b  M12R  Oak35874  POTl-uncult  RO1  RO2  RO3  RO5  RO6  RO7  RO8  TO-1P  TO-3S2P | DQ022853.1  DQ022855.1  DQ021520.1  DQ022848.1  DQ022849.1  DQ021518.1  EU526725.1  EU526726.1  EU526727.1  EU526728.1  EU526733.1  EU526732.1  EU526731.1  EU526734.1  EU526735.1  EU526736.1  EU526737.1  EU526740.1  EU526743.1  DQ021536.1  EU526744.1  EU526745.1  EU526746.1  EU526747.1  EU526748.1  EU526749.1  EU526750.1  EU526752.1  EU526751.1 | 87.5%  87.5%  75%  75%  75%  75%  75%  75%  75%  75%  75%  75%  75%  75%  75%  75%  75%  75%  75%  75%  75%  75%  75%  75%  75%  75%  75%  75%  75% | A76C  A76C  Incomplete  Incomplete  Incomplete  Incomplete  Incomplete  Incomplete  Incomplete  Incomplete  Incomplete  Incomplete  Incomplete  Incomplete  Incomplete  Incomplete  Incomplete  Incomplete  Incomplete  Incomplete  Incomplete  Incomplete  Incomplete  Incomplete  Incomplete  Incomplete  Incomplete  Incomplete  Incomplete | USA (2004) ^e^  USA (2004) ^e^  USA (2004) ^e^  USA (2004) ^e^  USA (2004) ^e^  USA (2004) ^e^  USA (<2008)  USA (<2008)  USA (<2008)  USA (<2008)  USA (<2008)  USA (<2008)  USA (<2008)  USA (<2008)  USA (<2008)  USA (<2008)  USA (<2008)  USA (<2008)  USA (<2008)  USA (2003) ^e^  USA (2002) ^e^  USA (2003) ^e^  USA (2003) ^e^  USA (<2008)  USA (<2008)  USA (<2008)  USA (<2008)  USA (<2008)  USA (<2008) | Pin oak  Pin oak  Sugar maple  Pin oak  Pin oak  Pin oak  Blueberry  Blueberry  Blueberry  Blueberry  Elm  Ellis oak  Ellis oak  Plum  Plum  Plum  Plum  Almond  Oak  Pin oak  Red oak  Red oak  Red oak  Red oak  Red oak  Red oak  Red oak  Turkey oak  Turkey oak |
| *sandyi* [Co33] (n=0) | - | - | - | - | - | - |
| *sandyi* [Ann-1] (n=2) | GH-9  Ol | DQ991185.1  DQ991186.1 | 100%  100% | -  - | USA (1999) ^f^  USA (1999) ^f^ | Oleander ^f^  Oleander ^f^ |
| *pauca* (n=5) | P3 | AF536769.1 | 100% | - | Brazil (<2003) ^g^ | *Coffea arabica* ^g^ |
|  | OL-G2  PS-1  PS-2  PW-1 | KJ406215.1  KJ631115.1  KJ631116.1  KJ406258.1 | 87.5%  75%  75%  75% | G76A  Incomplete  Incomplete  Incomplete | Italy (2013)  Italy (2013)  Italy (2013)  Italy (<2014) | *Olea* *europaea* cv. Ogliarola  spittlebug  spittlebug  Perwinca |

^a^ Name of *X. fastidiosa* strains or isolates, except for uncultured materials (uncult).

^b^ SNP code correspondence: percentage of the eight discriminant positions (Table 5) included in the 16S rRNA gene sequence.

^c^ SNP code variability: reason why the correspondence is not 100%; Incomplete: the two first positions (75 and 76) are not covered by the shorter sequences; C1340T, A76C and G76A: substitutions events occurring in the sequences compared to expected nucleotides at these positions, according to Table 9.

^d^ Country, Year and Host of isolation as described in the metadata associated with the release of the 16S rRNA accessions, except if mentioned.

^e^ Mundell JN. Phylogenetic analysis of Kenticky strains of *Xylella fastidiosa*. University of Kentucky Master's Theses. 2005;406. https://uknowledge.uky.edu/gradschool_theses/406

^f^ Su CC, Chang CJ, Yang WJ, Hsu ST, Tzeng KC, Jan FJ et al. Specific characters of 16S rRNA gene and 16S–23S rRNA internal transcribed spacer sequences of *Xylella* *fastidiosa* pear leaf scorch strains. Eur. J. Plant Pathol. 2012;132(2):203-16.

^g^ Rodrigues JL, Silva-Stenico ME, Gomes JE, Lopes JR, Tsai SM. Detection and diversity assessment of *Xylella* *fastidiosa* in field-collected plant and insect samples by using 16S rRNA and gyrB sequences. Appl. Environ. Microbiol. 2003;69(7):4249-55.
