# Supplementary figures and images for: Identification of genetic relationships and subspecies signatures in Xylella fastidiosa
Source: BMC Genomics. 2019 Mar 25;20:239. doi: 10.1186/s12864-019-5565-9 (PMC6434890; doi:10.1186/s12864-019-5565-9)

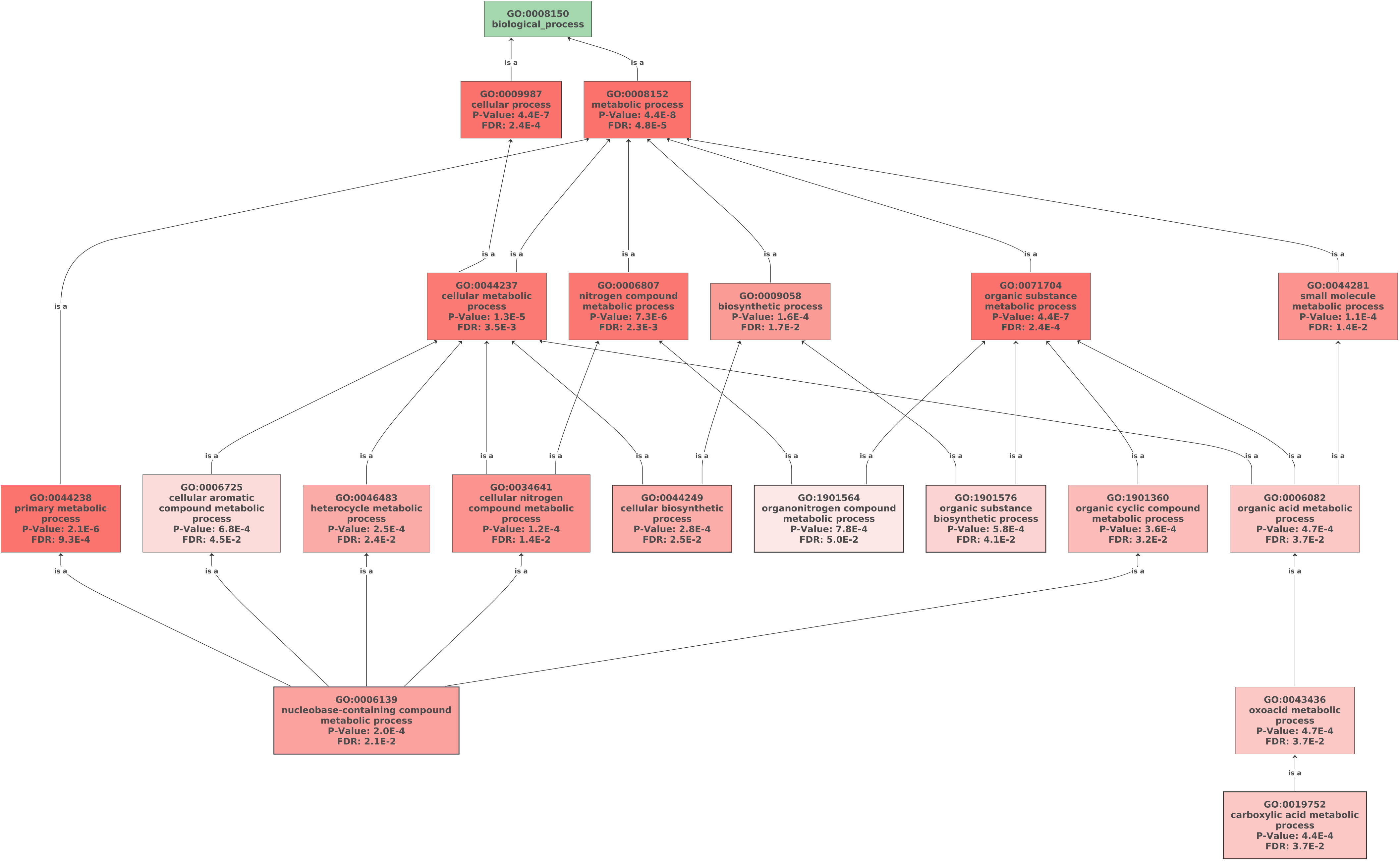

Supplement: Supplementary file 7 — Raw data of the gene ontologies enrichments tests with Blast2GO. (ZIP 22422 kb) [file 12864_2019_5565_MOESM7_ESM.zip › Additional-File-7/Group_FAS2/blast2go_FAS2_enriched_bp.png]

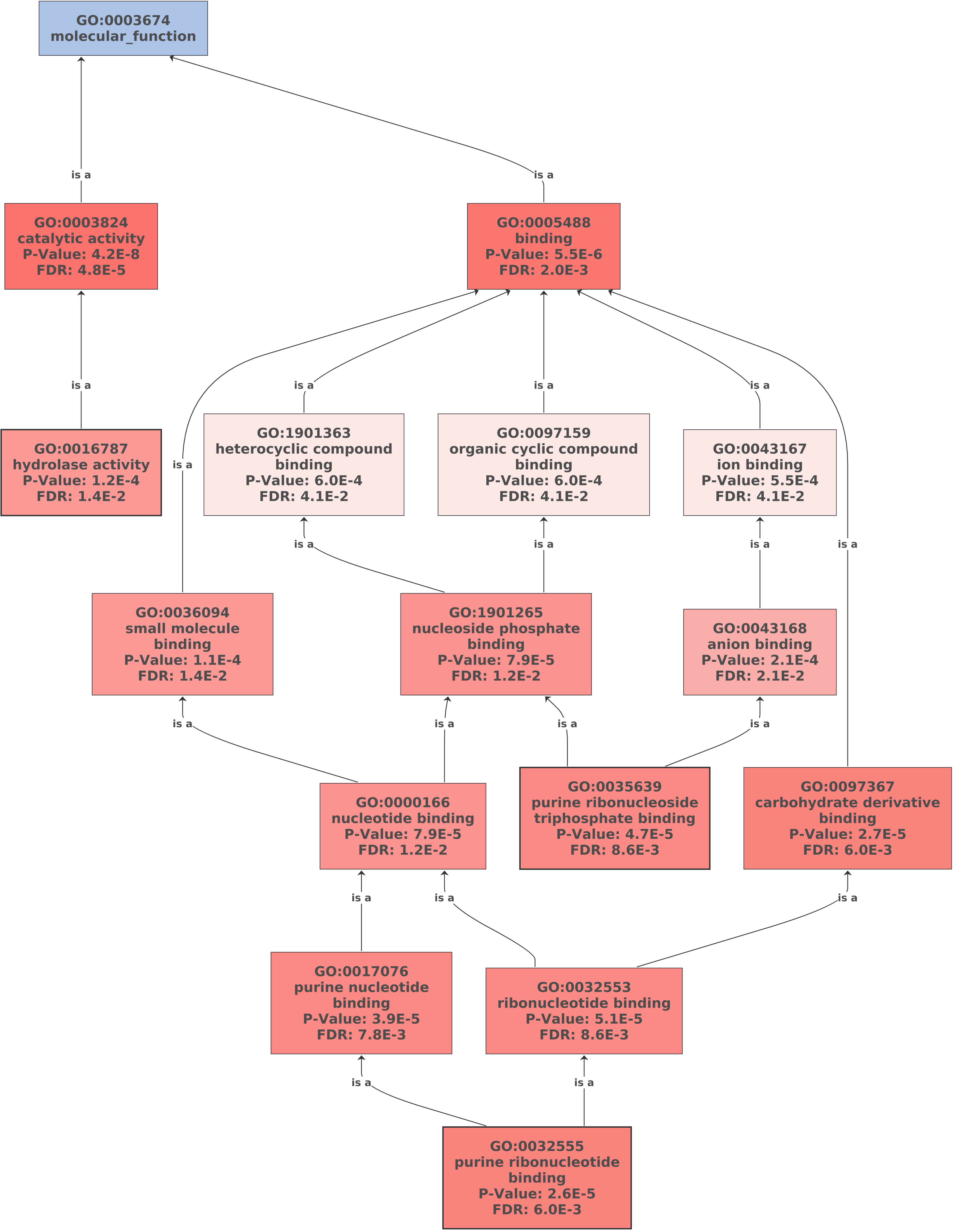

Supplement: Supplementary file 7 — Raw data of the gene ontologies enrichments tests with Blast2GO. (ZIP 22422 kb) [file 12864_2019_5565_MOESM7_ESM.zip › Additional-File-7/Group_FAS2/blast2go_FAS2_enriched_mf.png]

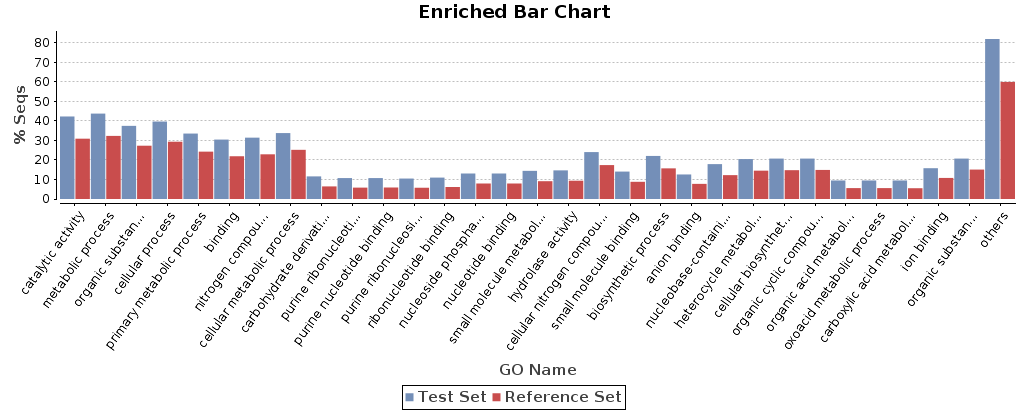

Supplement: Supplementary file 7 — Raw data of the gene ontologies enrichments tests with Blast2GO. (ZIP 22422 kb) [file 12864_2019_5565_MOESM7_ESM.zip › Additional-File-7/Group_FAS2/blast2go_statistics_FAS2.png]

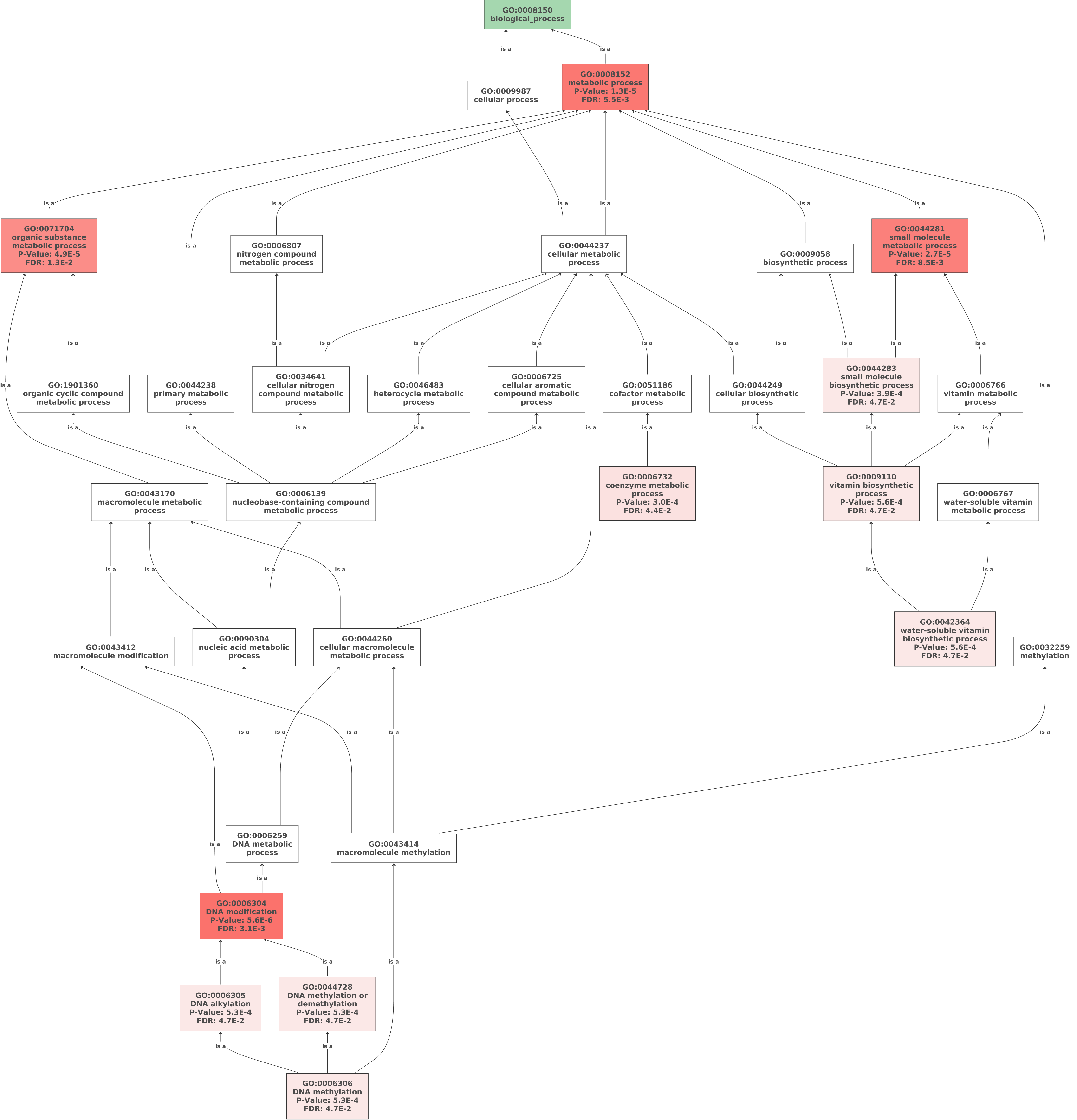

Supplement: Supplementary file 7 — Raw data of the gene ontologies enrichments tests with Blast2GO. (ZIP 22422 kb) [file 12864_2019_5565_MOESM7_ESM.zip › Additional-File-7/Group_FAS/blast2go_FAS_enriched_bp.png]

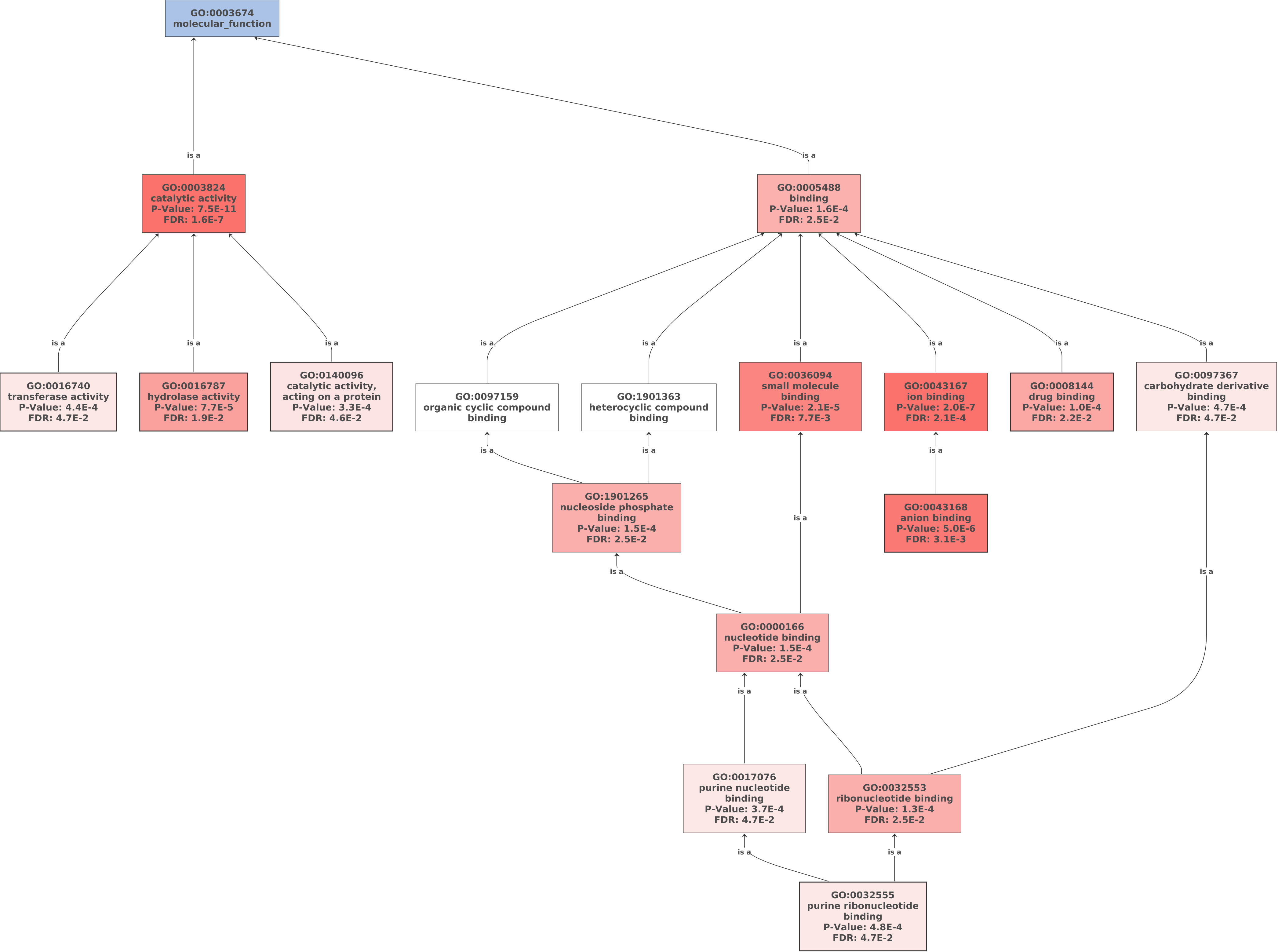

Supplement: Supplementary file 7 — Raw data of the gene ontologies enrichments tests with Blast2GO. (ZIP 22422 kb) [file 12864_2019_5565_MOESM7_ESM.zip › Additional-File-7/Group_FAS/blast2go_FAS_enriched_mf.png]

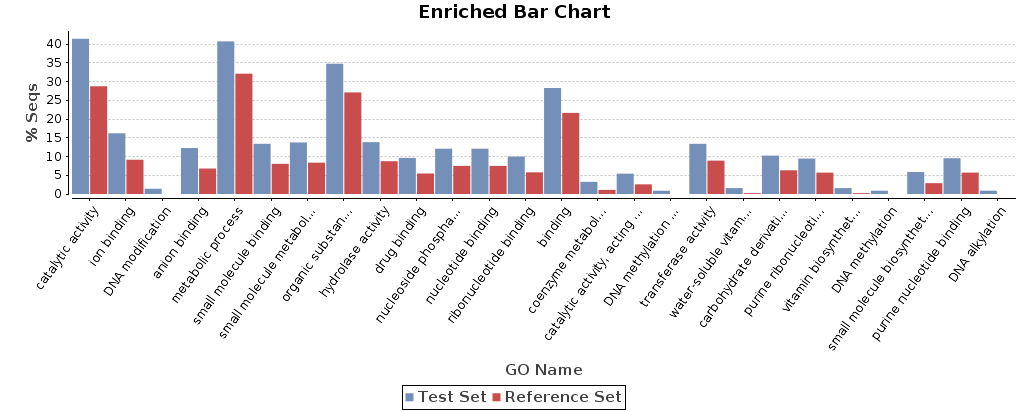

Supplement: Supplementary file 7 — Raw data of the gene ontologies enrichments tests with Blast2GO. (ZIP 22422 kb) [file 12864_2019_5565_MOESM7_ESM.zip › Additional-File-7/Group_FAS/blast2go_statistics_FAS.png]

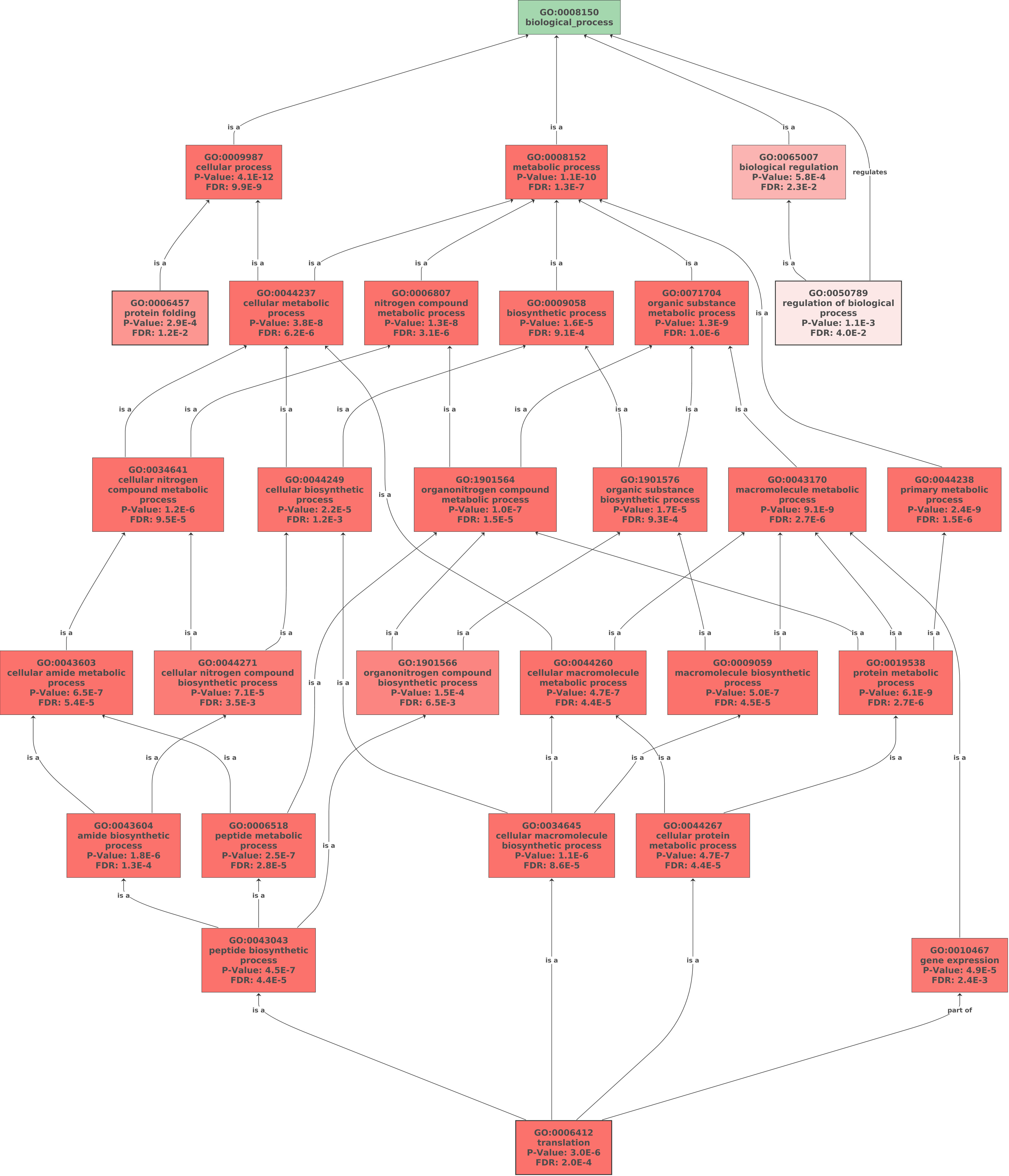

Supplement: Supplementary file 7 — Raw data of the gene ontologies enrichments tests with Blast2GO. (ZIP 22422 kb) [file 12864_2019_5565_MOESM7_ESM.zip › Additional-File-7/Group_MOR-FAS2/blast2go_MOR-FAS2_enriched_bp.png]

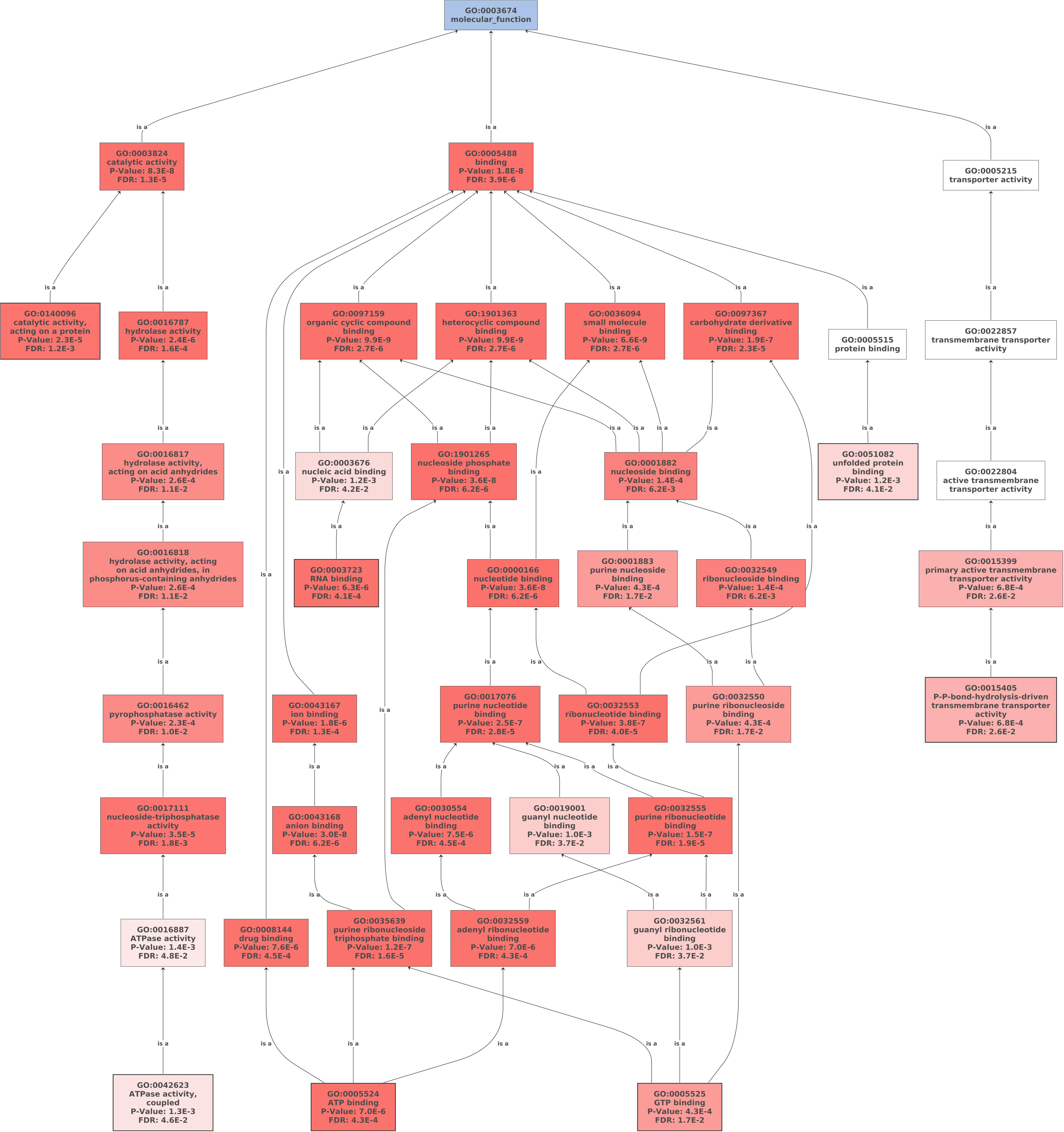

Supplement: Supplementary file 7 — Raw data of the gene ontologies enrichments tests with Blast2GO. (ZIP 22422 kb) [file 12864_2019_5565_MOESM7_ESM.zip › Additional-File-7/Group_MOR-FAS2/blast2go_MOR-FAS2_enriched_mf.png]

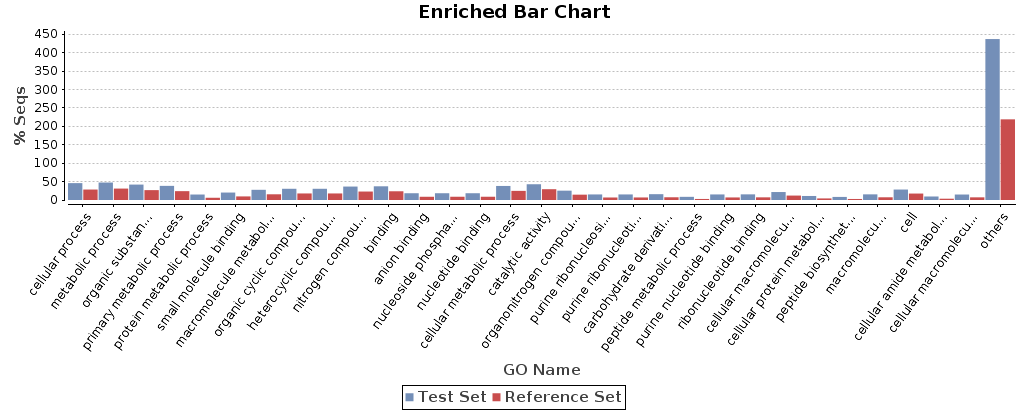

Supplement: Supplementary file 7 — Raw data of the gene ontologies enrichments tests with Blast2GO. (ZIP 22422 kb) [file 12864_2019_5565_MOESM7_ESM.zip › Additional-File-7/Group_MOR-FAS2/blast2go_statistics_MOR-FAS2.png]

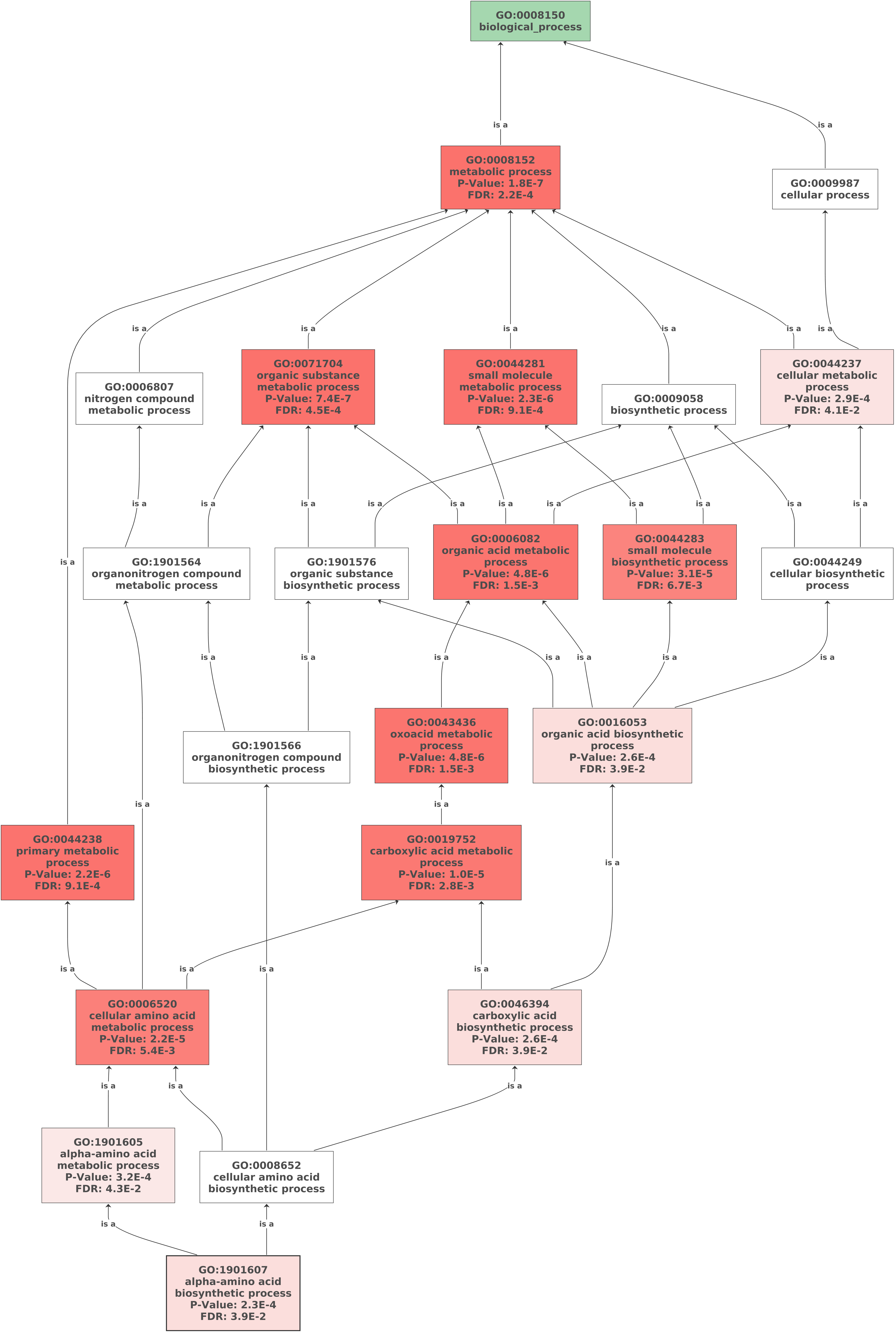

Supplement: Supplementary file 7 — Raw data of the gene ontologies enrichments tests with Blast2GO. (ZIP 22422 kb) [file 12864_2019_5565_MOESM7_ESM.zip › Additional-File-7/Group_MOR-MUL/blast2go_MOR-MUL_enriched_bp.png]

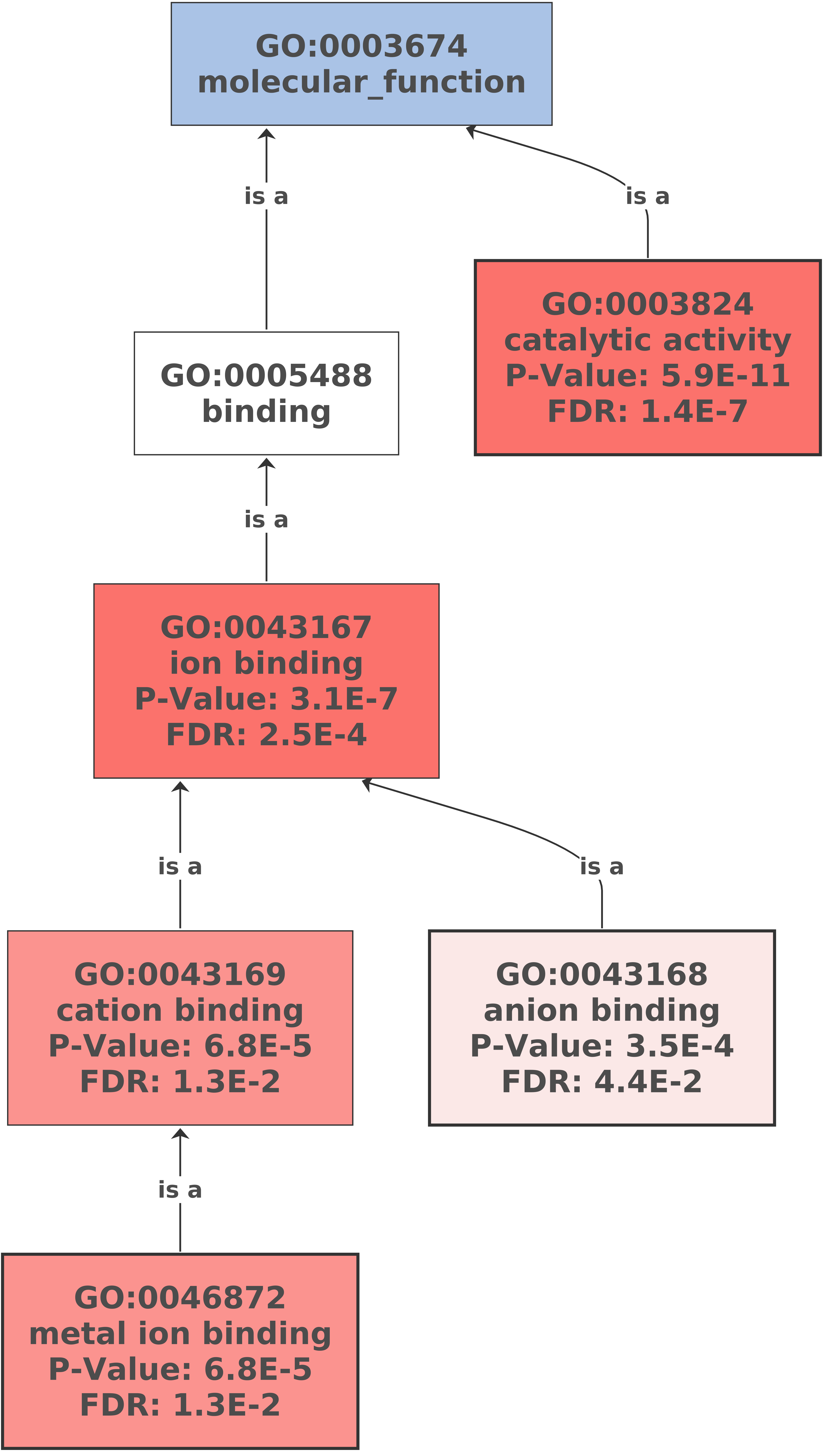

Supplement: Supplementary file 7 — Raw data of the gene ontologies enrichments tests with Blast2GO. (ZIP 22422 kb) [file 12864_2019_5565_MOESM7_ESM.zip › Additional-File-7/Group_MOR-MUL/blast2go_MOR-MUL_enriched_mf.png]

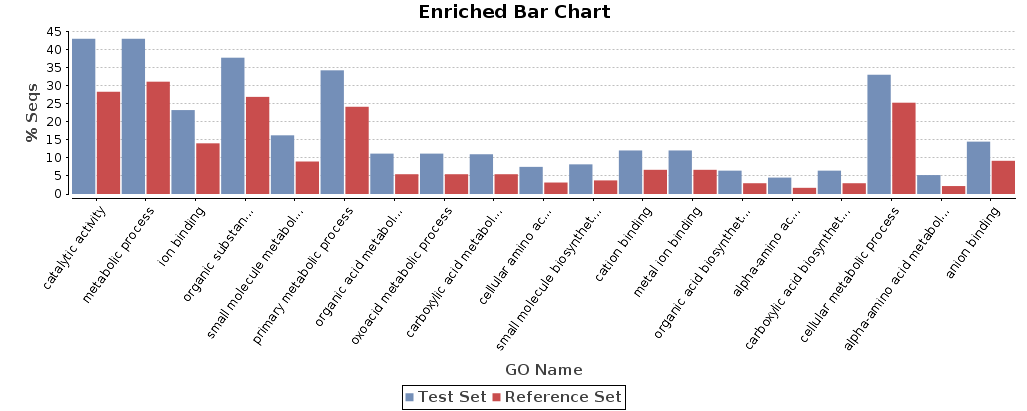

Supplement: Supplementary file 7 — Raw data of the gene ontologies enrichments tests with Blast2GO. (ZIP 22422 kb) [file 12864_2019_5565_MOESM7_ESM.zip › Additional-File-7/Group_MOR-MUL/blast2go_statistics_MOR-MUL.png]

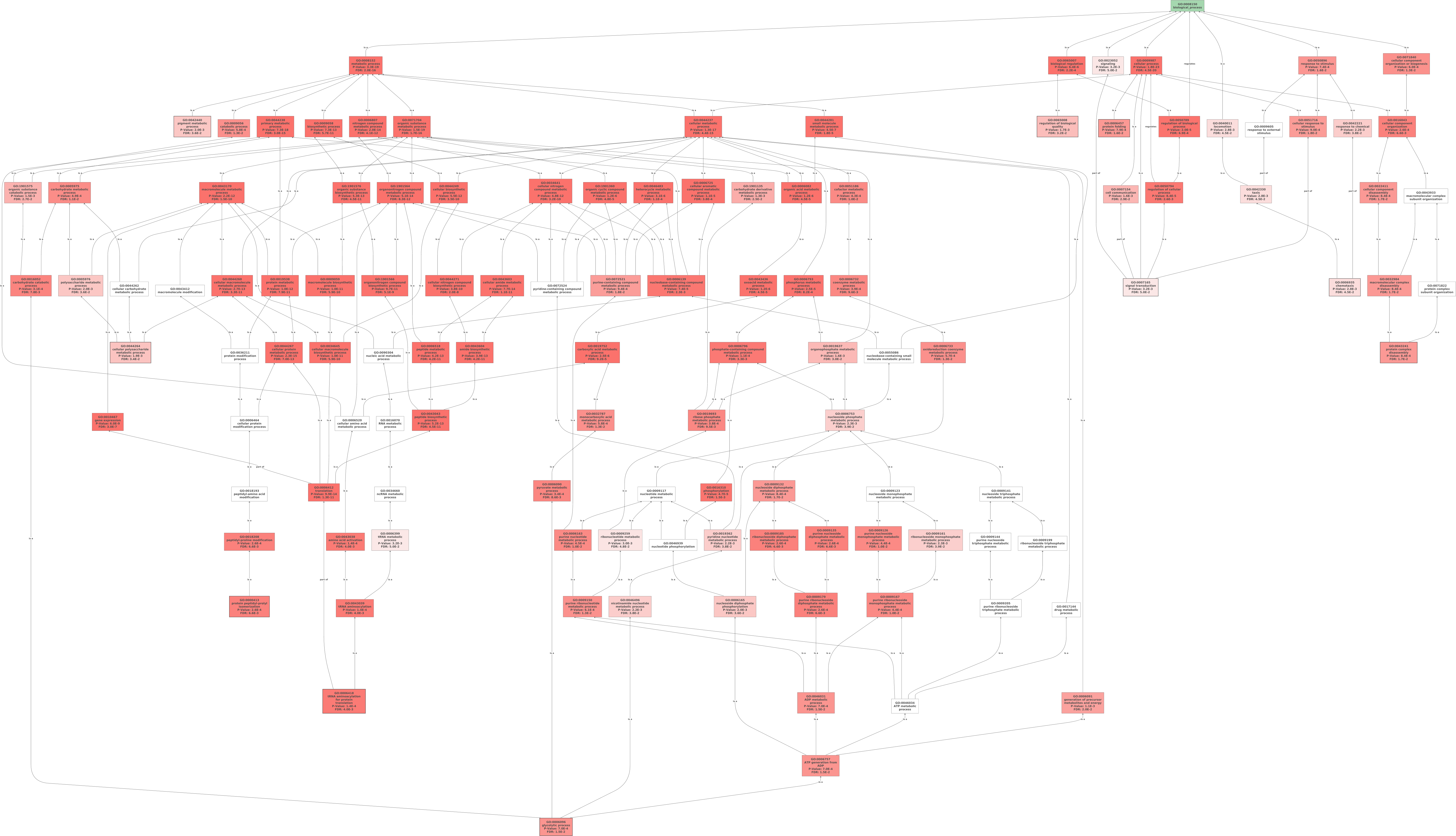

Supplement: Supplementary file 7 — Raw data of the gene ontologies enrichments tests with Blast2GO. (ZIP 22422 kb) [file 12864_2019_5565_MOESM7_ESM.zip › Additional-File-7/Group_MOR-SAN-SAN2-FAS2/blast2go_MOR-FAS2-SAN-SAN2_enriched_bp.png]

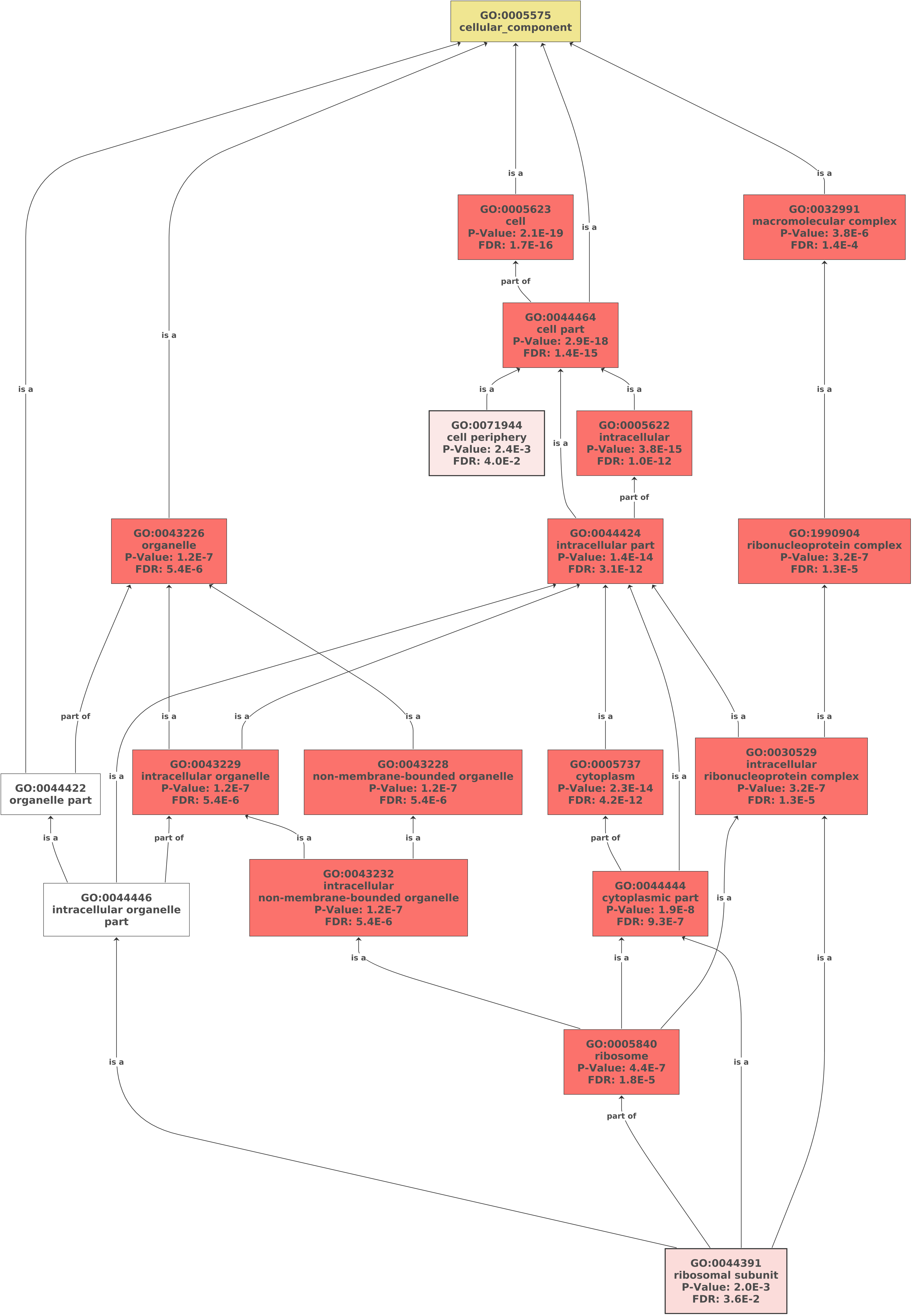

Supplement: Supplementary file 7 — Raw data of the gene ontologies enrichments tests with Blast2GO. (ZIP 22422 kb) [file 12864_2019_5565_MOESM7_ESM.zip › Additional-File-7/Group_MOR-SAN-SAN2-FAS2/blast2go_MOR-FAS2-SAN-SAN2_enriched_cc.png]

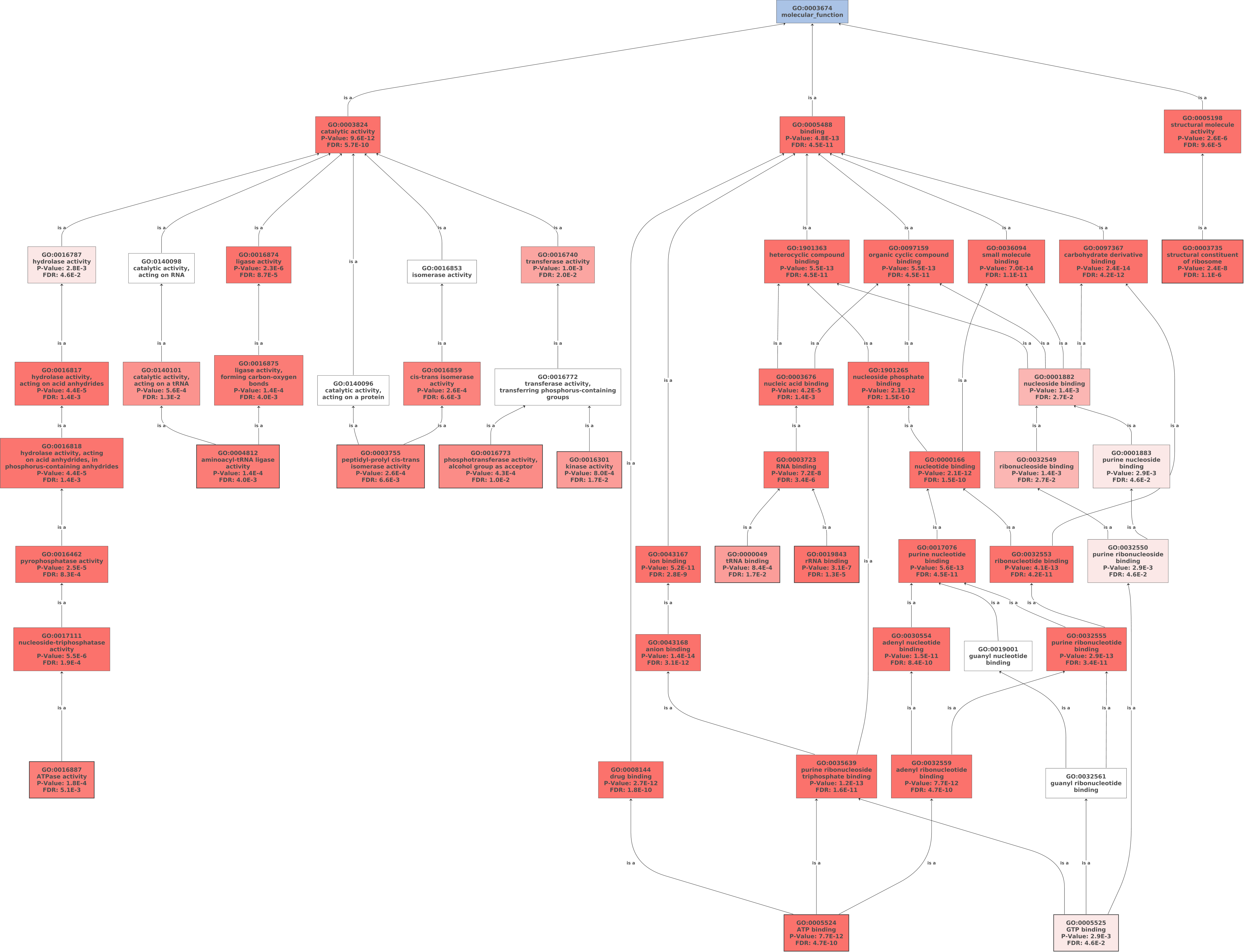

Supplement: Supplementary file 7 — Raw data of the gene ontologies enrichments tests with Blast2GO. (ZIP 22422 kb) [file 12864_2019_5565_MOESM7_ESM.zip › Additional-File-7/Group_MOR-SAN-SAN2-FAS2/blast2go_MOR-FAS2-SAN-SAN2_enriched_mf.png]

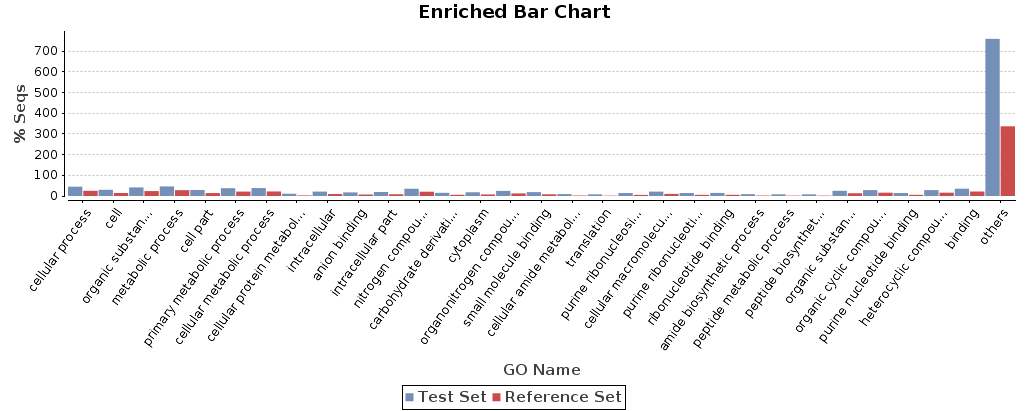

Supplement: Supplementary file 7 — Raw data of the gene ontologies enrichments tests with Blast2GO. (ZIP 22422 kb) [file 12864_2019_5565_MOESM7_ESM.zip › Additional-File-7/Group_MOR-SAN-SAN2-FAS2/blast2go_statistics_MOR-FAS2-SAN-SAN2.png]

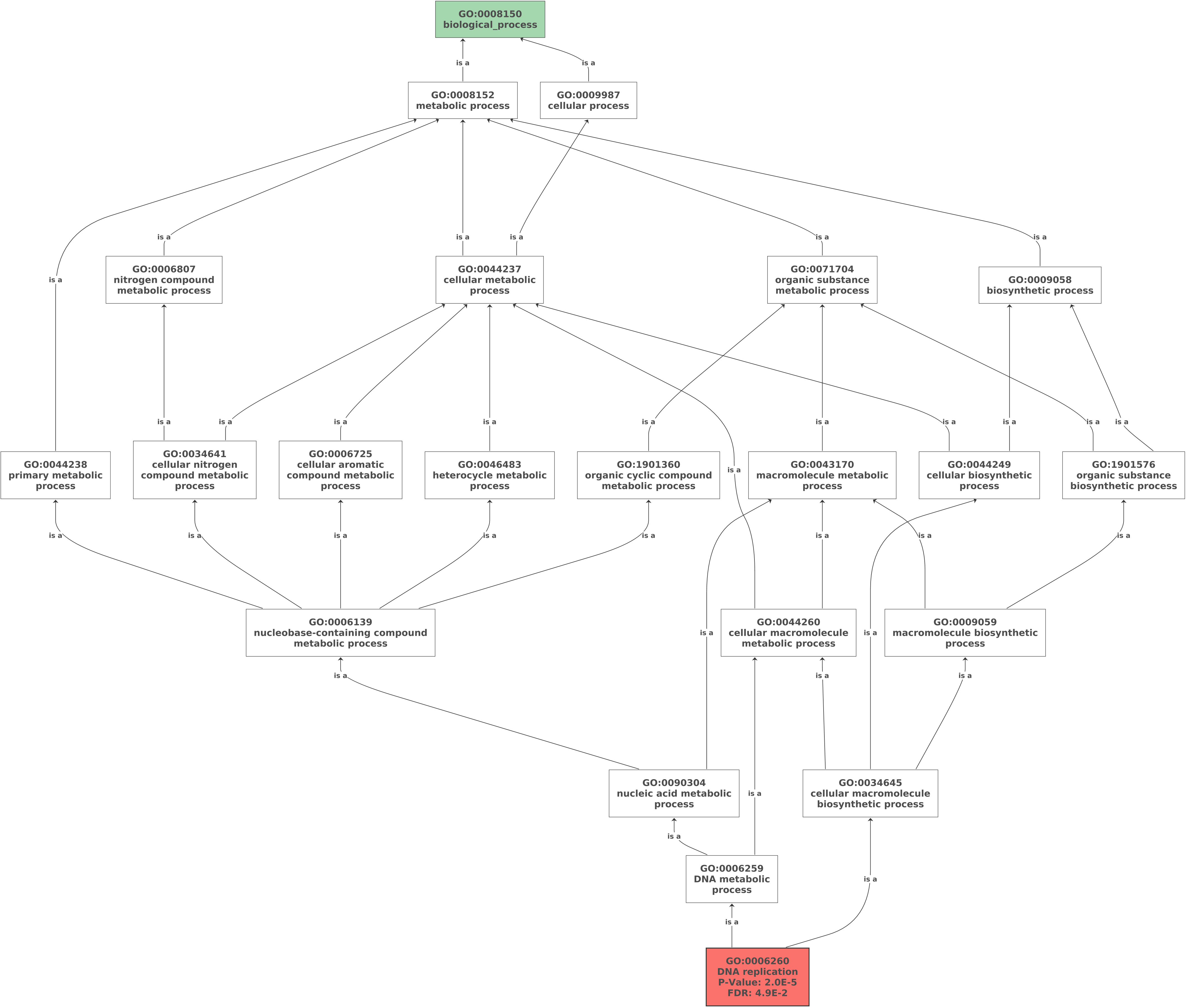

Supplement: Supplementary file 7 — Raw data of the gene ontologies enrichments tests with Blast2GO. (ZIP 22422 kb) [file 12864_2019_5565_MOESM7_ESM.zip › Additional-File-7/Group_MOR/blast2go_MOR_enriched_bp.png]

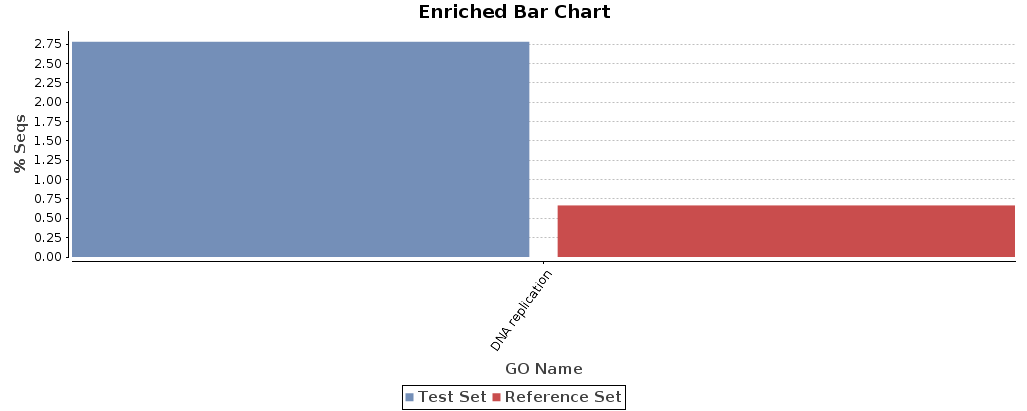

Supplement: Supplementary file 7 — Raw data of the gene ontologies enrichments tests with Blast2GO. (ZIP 22422 kb) [file 12864_2019_5565_MOESM7_ESM.zip › Additional-File-7/Group_MOR/blast2go_statistics_MOR.png]

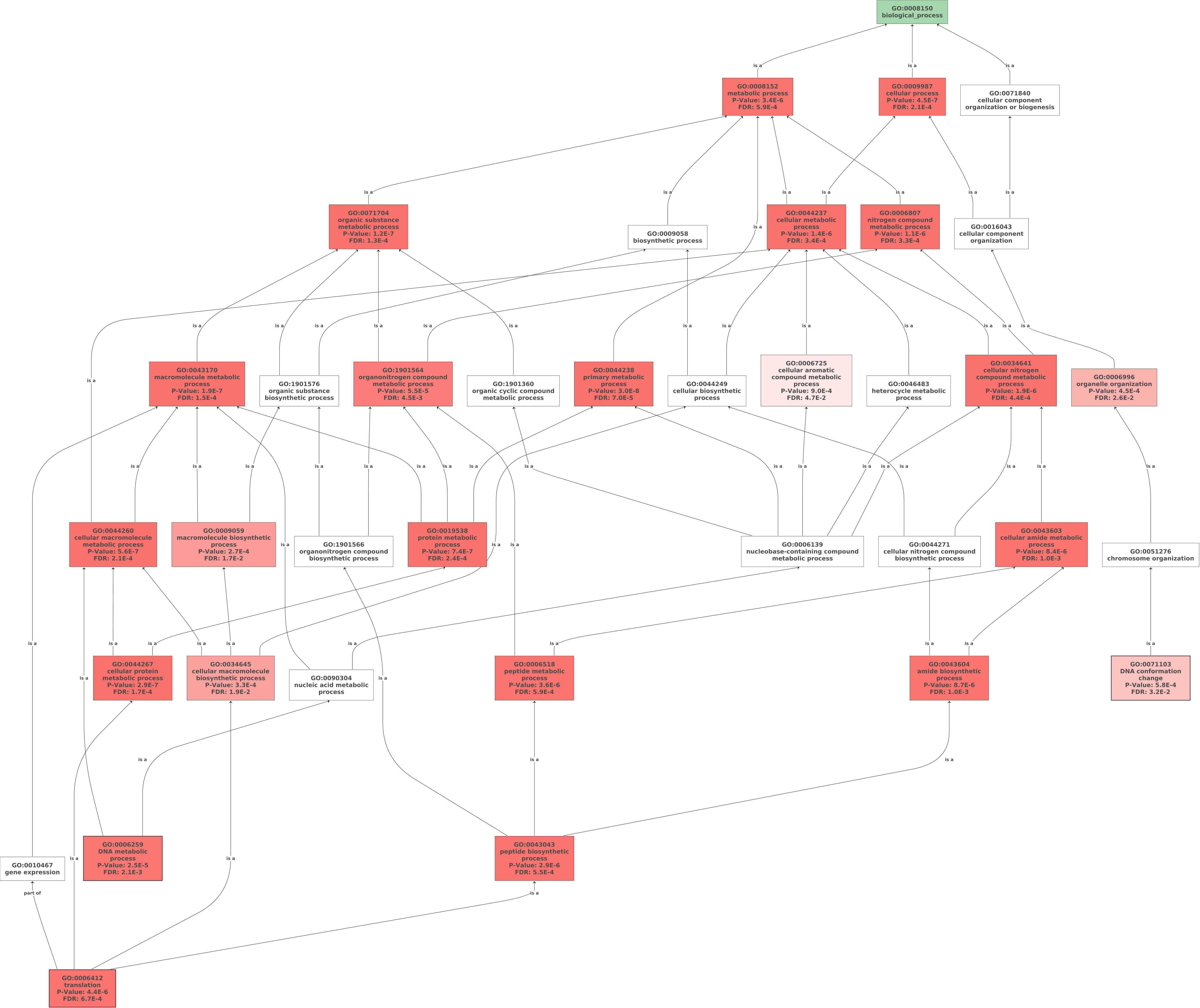

Supplement: Supplementary file 7 — Raw data of the gene ontologies enrichments tests with Blast2GO. (ZIP 22422 kb) [file 12864_2019_5565_MOESM7_ESM.zip › Additional-File-7/Group_MUL/blast2go_MUL_enriched_bp.png]

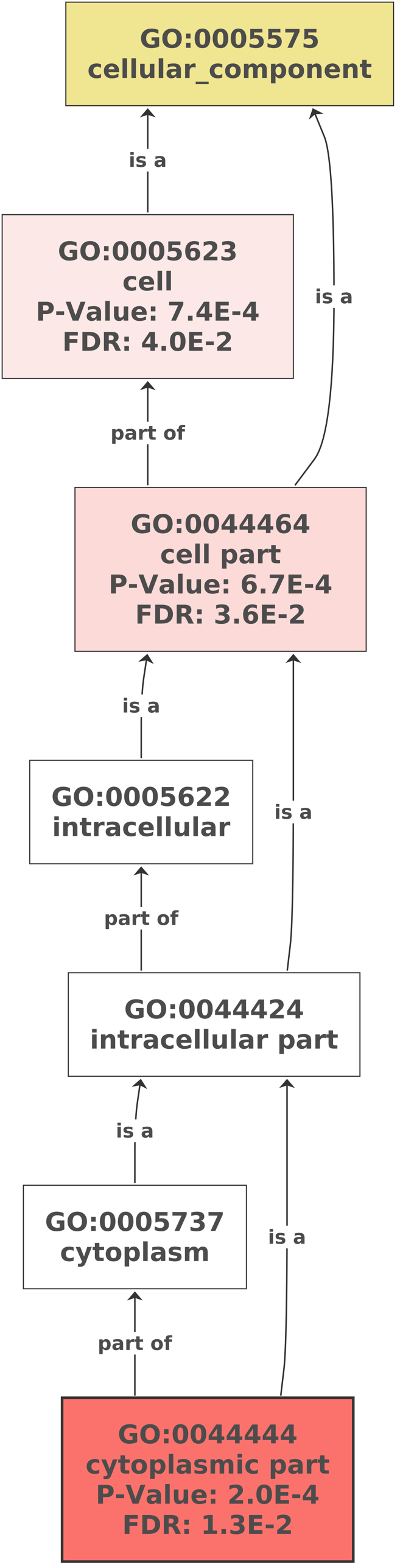

Supplement: Supplementary file 7 — Raw data of the gene ontologies enrichments tests with Blast2GO. (ZIP 22422 kb) [file 12864_2019_5565_MOESM7_ESM.zip › Additional-File-7/Group_MUL/blast2go_MUL_enriched_cc.png]

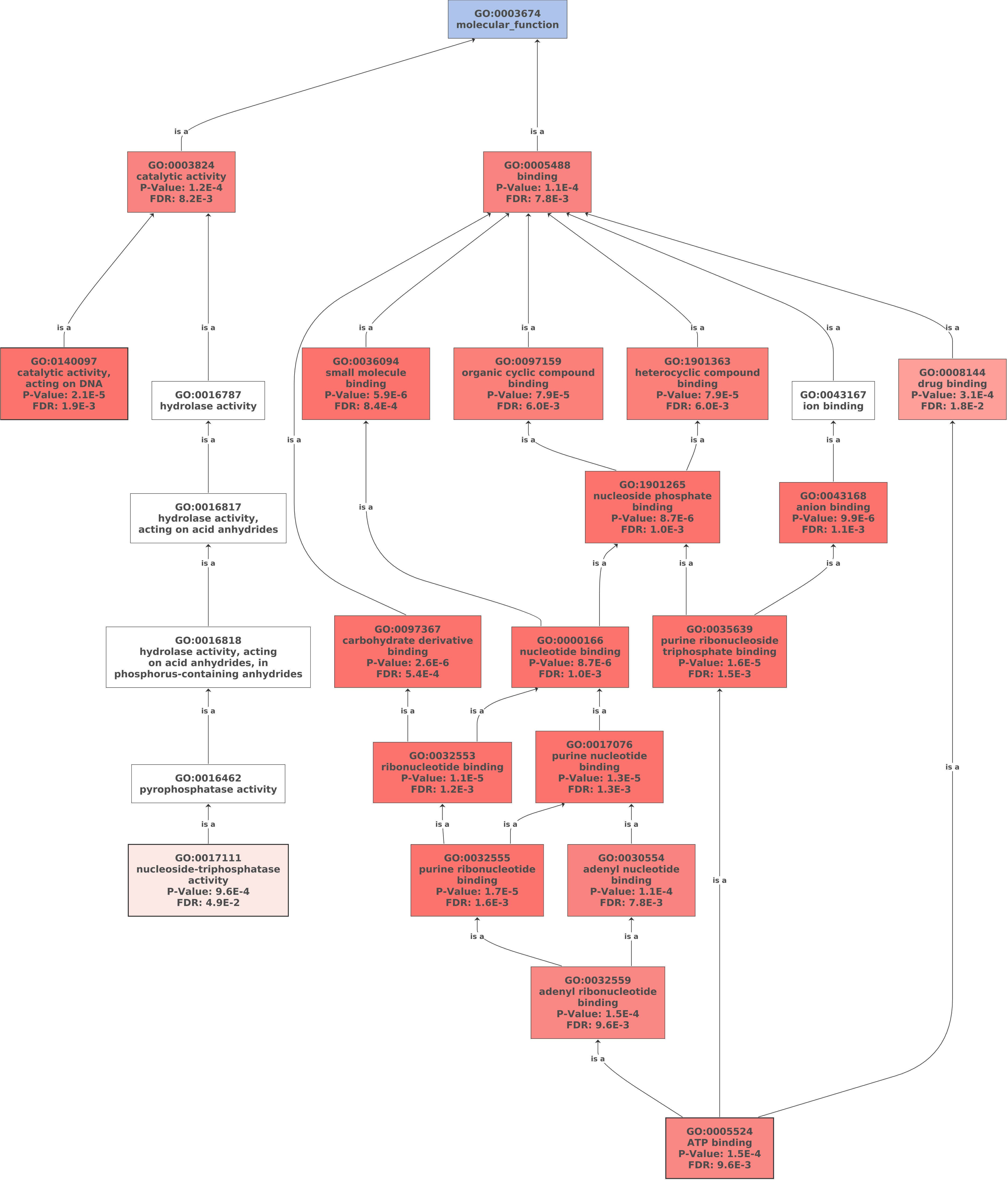

Supplement: Supplementary file 7 — Raw data of the gene ontologies enrichments tests with Blast2GO. (ZIP 22422 kb) [file 12864_2019_5565_MOESM7_ESM.zip › Additional-File-7/Group_MUL/blast2go_MUL_enriched_mf.png]

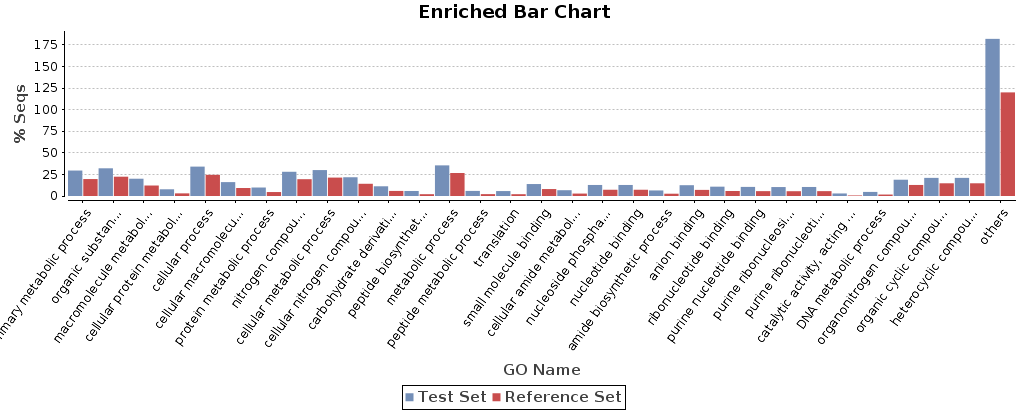

Supplement: Supplementary file 7 — Raw data of the gene ontologies enrichments tests with Blast2GO. (ZIP 22422 kb) [file 12864_2019_5565_MOESM7_ESM.zip › Additional-File-7/Group_MUL/blast2go_statistics_MUL.png]

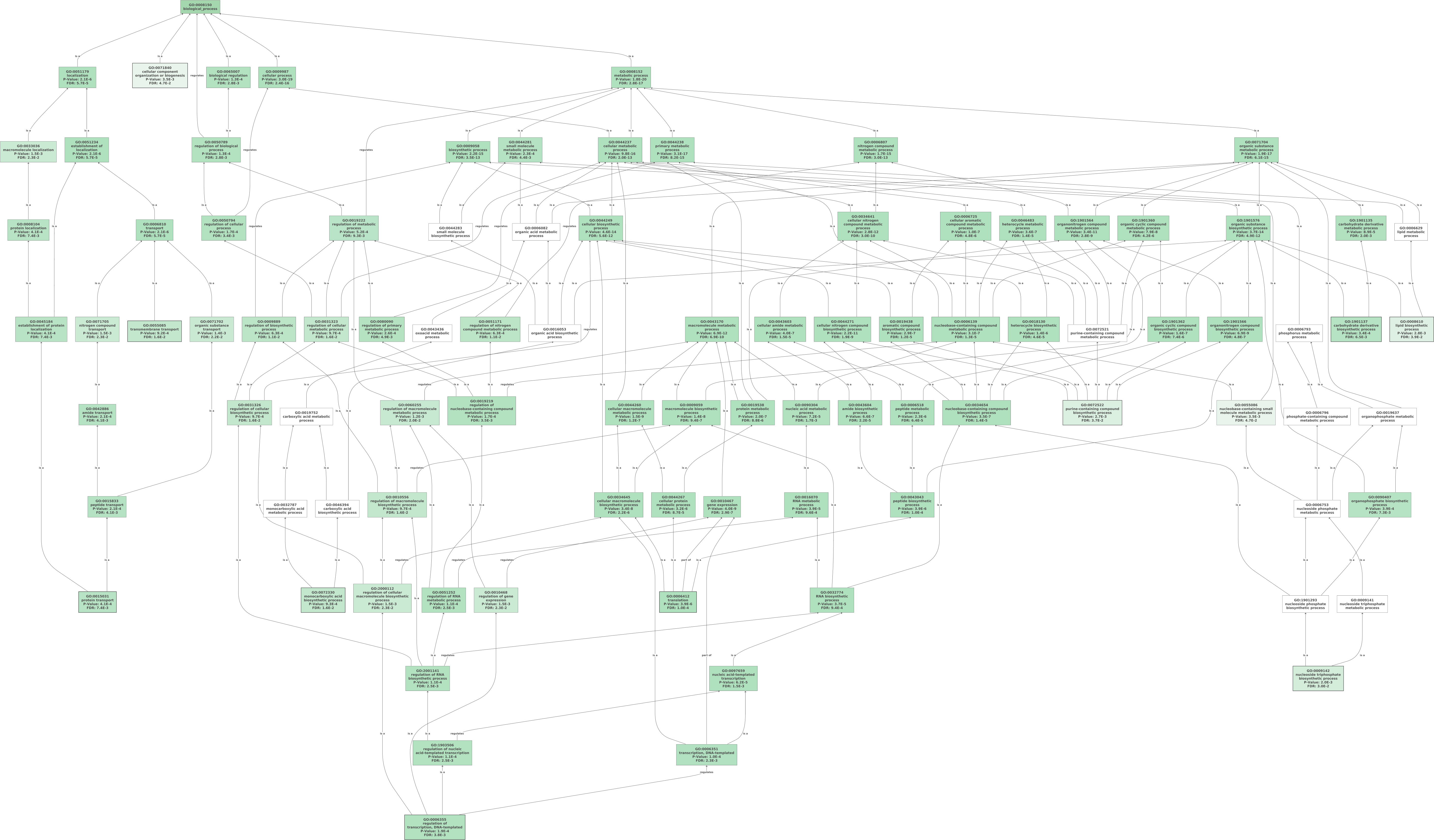

Supplement: Supplementary file 7 — Raw data of the gene ontologies enrichments tests with Blast2GO. (ZIP 22422 kb) [file 12864_2019_5565_MOESM7_ESM.zip › Additional-File-7/Group_PAU-CFBP8072/blast2go_8072_enriched_bp.png]

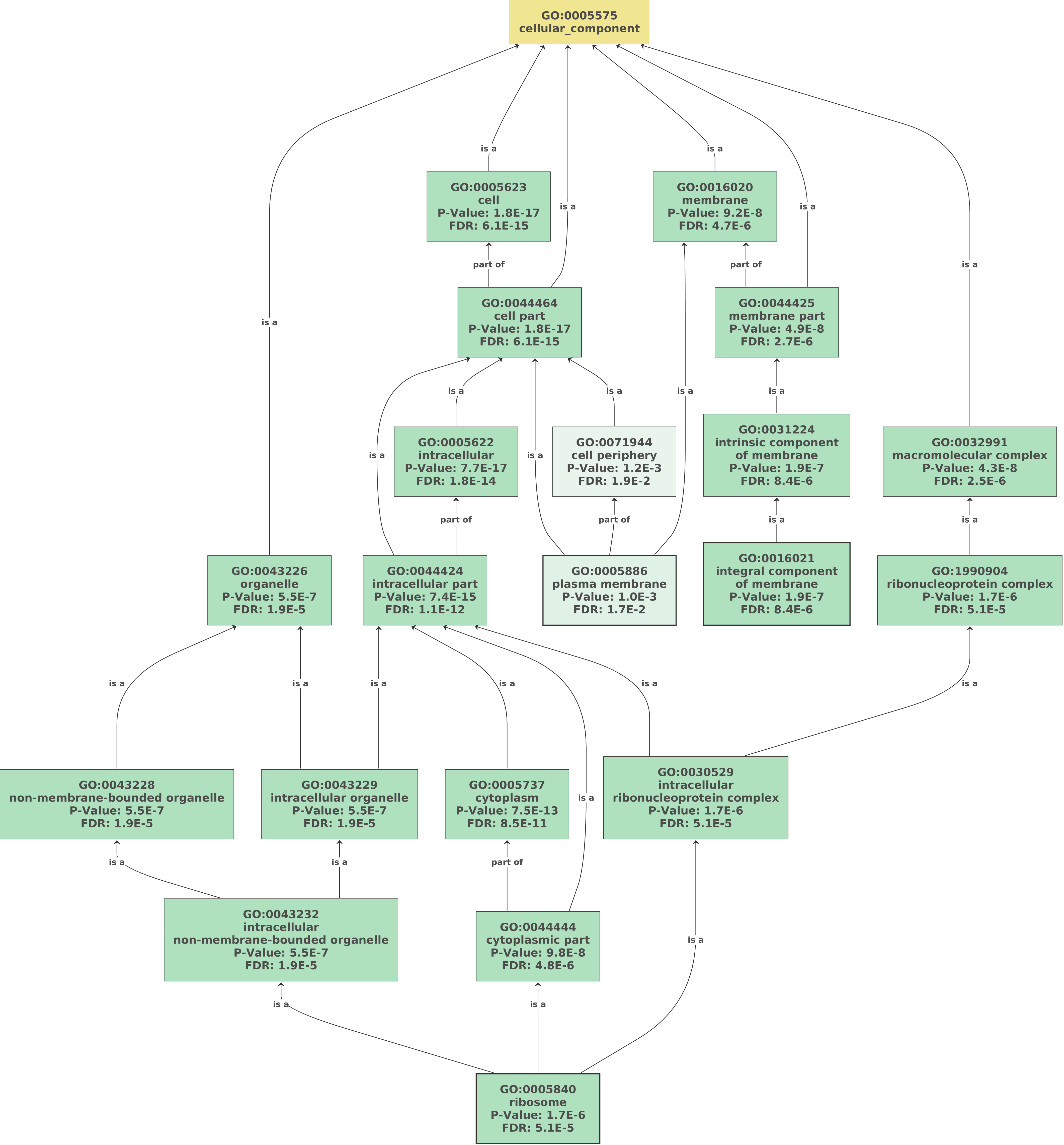

Supplement: Supplementary file 7 — Raw data of the gene ontologies enrichments tests with Blast2GO. (ZIP 22422 kb) [file 12864_2019_5565_MOESM7_ESM.zip › Additional-File-7/Group_PAU-CFBP8072/blast2go_8072_enriched_cc.png]

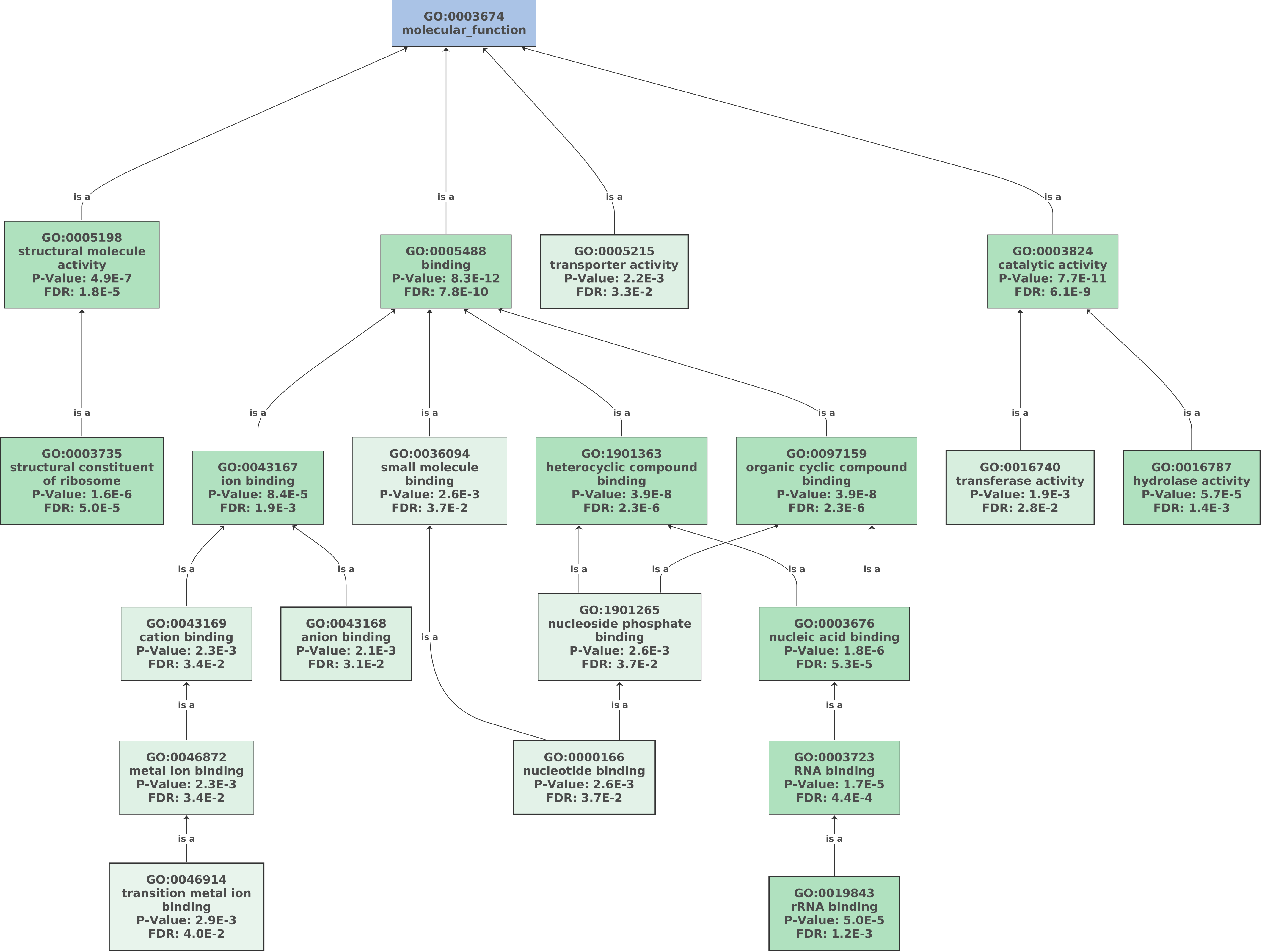

Supplement: Supplementary file 7 — Raw data of the gene ontologies enrichments tests with Blast2GO. (ZIP 22422 kb) [file 12864_2019_5565_MOESM7_ESM.zip › Additional-File-7/Group_PAU-CFBP8072/blast2go_8072_enriched_mf.png]

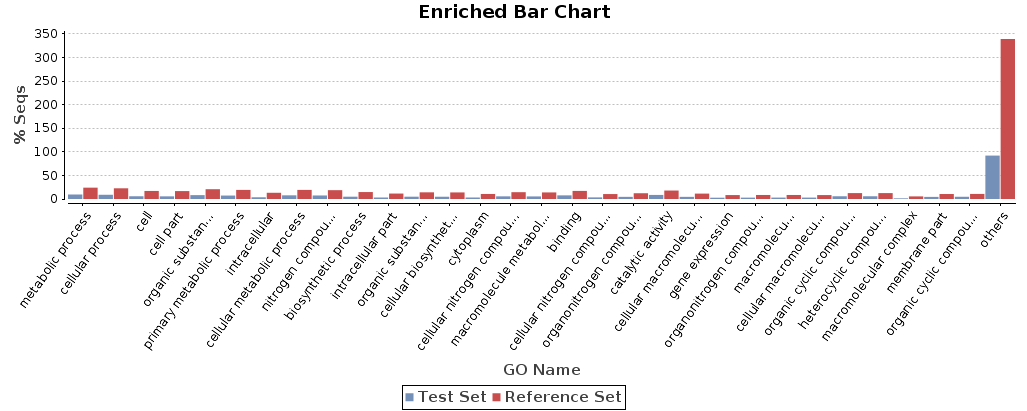

Supplement: Supplementary file 7 — Raw data of the gene ontologies enrichments tests with Blast2GO. (ZIP 22422 kb) [file 12864_2019_5565_MOESM7_ESM.zip › Additional-File-7/Group_PAU-CFBP8072/blast2go_statistics_8072.png]

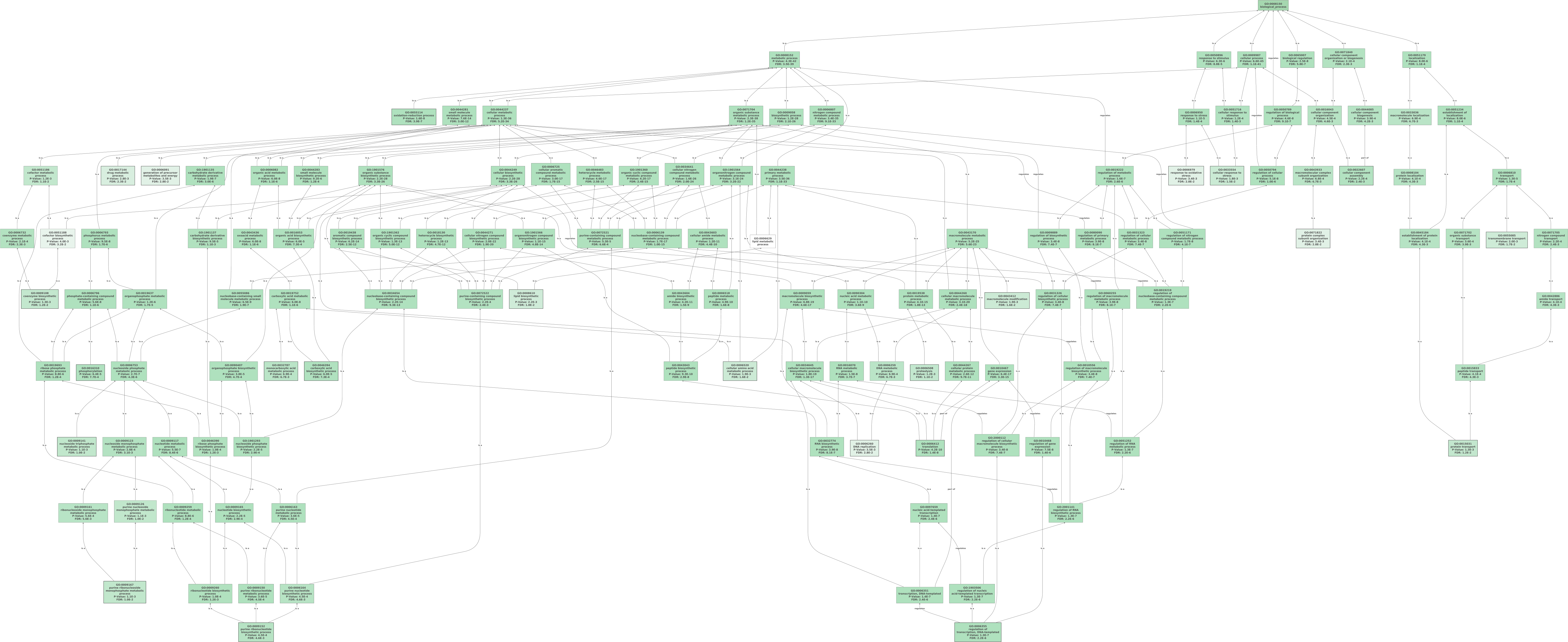

Supplement: Supplementary file 7 — Raw data of the gene ontologies enrichments tests with Blast2GO. (ZIP 22422 kb) [file 12864_2019_5565_MOESM7_ESM.zip › Additional-File-7/Group_PAU-Hib4/blast2go_hib4_enriched_bp.png]

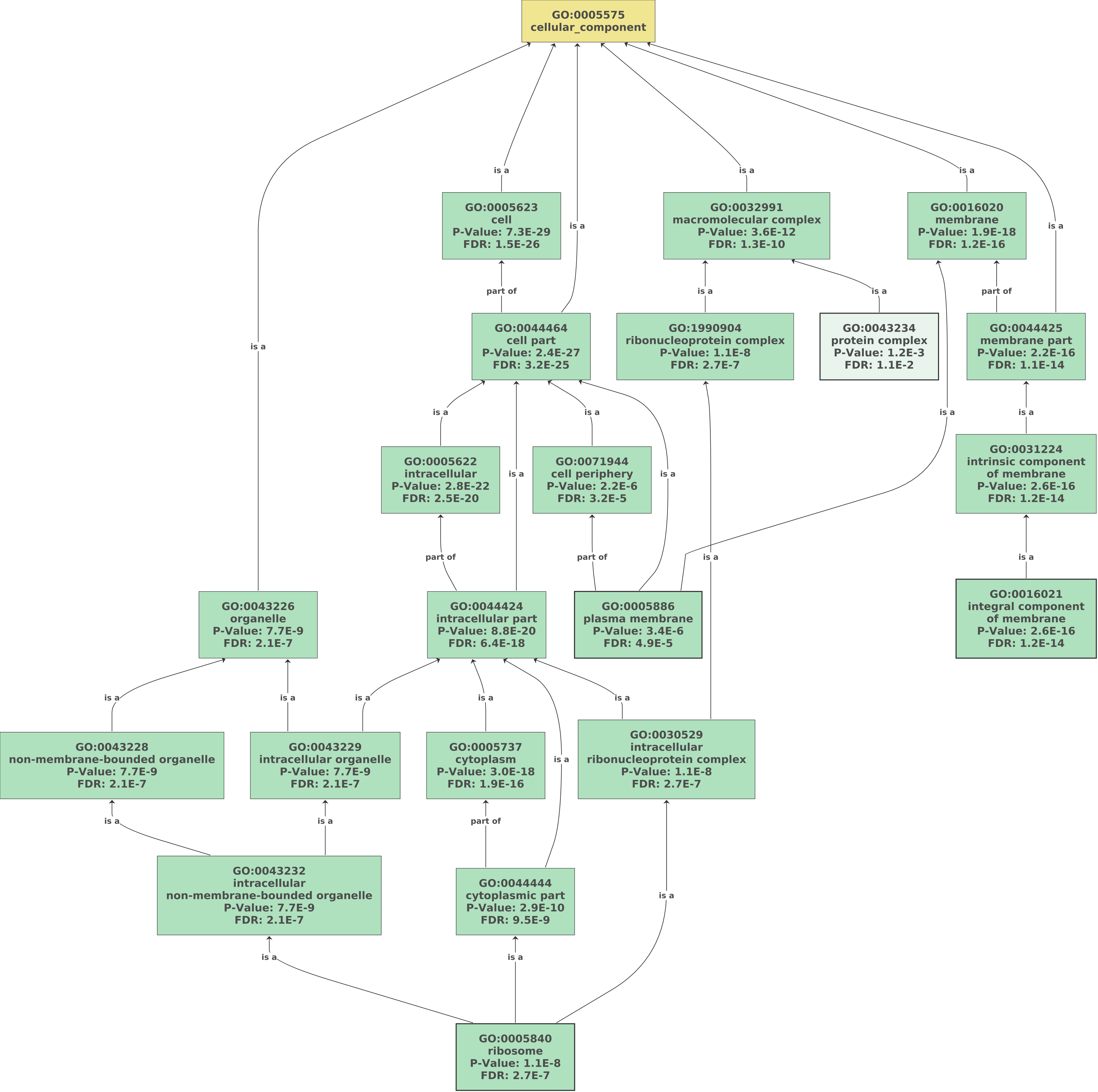

Supplement: Supplementary file 7 — Raw data of the gene ontologies enrichments tests with Blast2GO. (ZIP 22422 kb) [file 12864_2019_5565_MOESM7_ESM.zip › Additional-File-7/Group_PAU-Hib4/blast2go_hib4_enriched_cc.png]

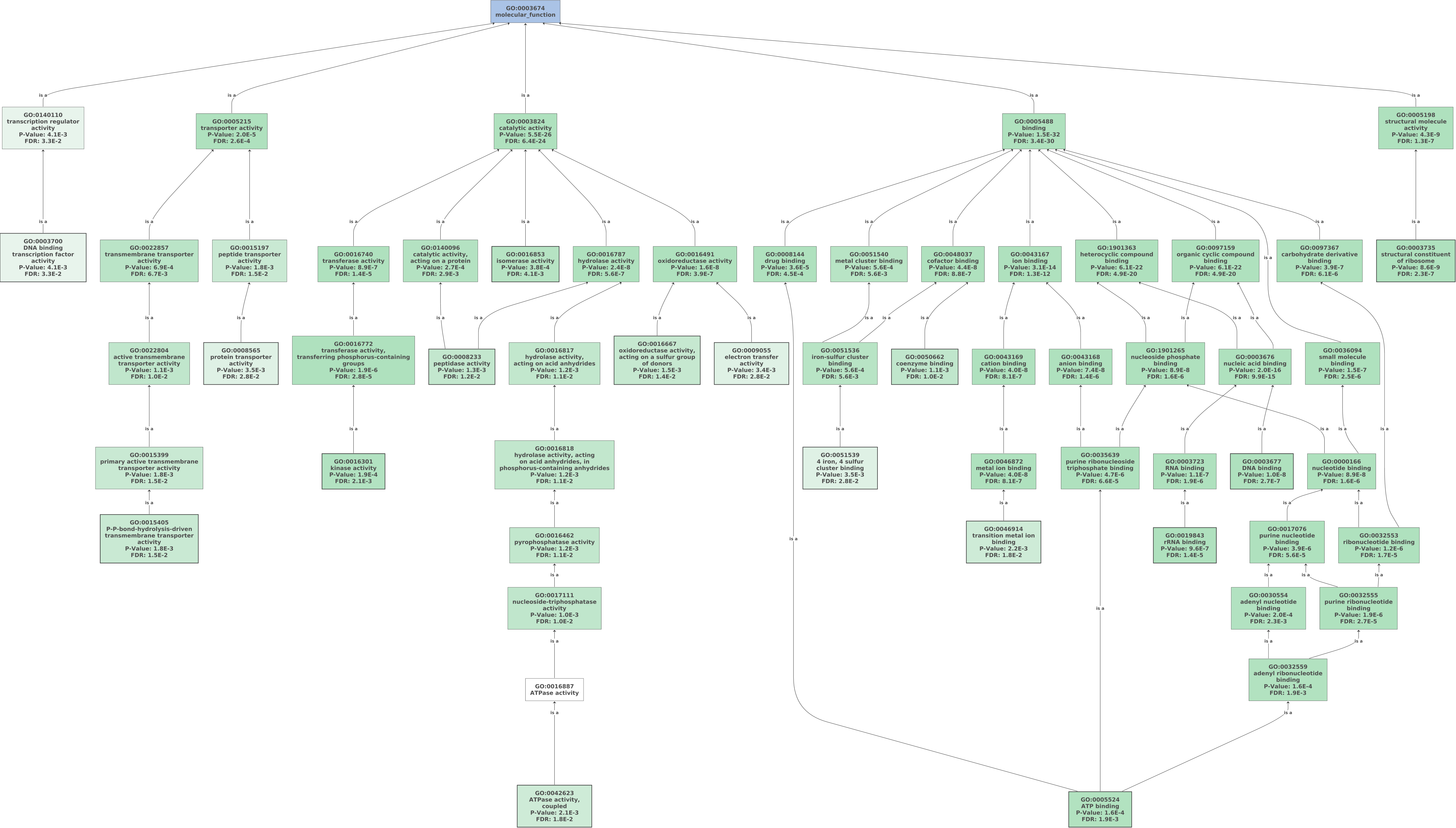

Supplement: Supplementary file 7 — Raw data of the gene ontologies enrichments tests with Blast2GO. (ZIP 22422 kb) [file 12864_2019_5565_MOESM7_ESM.zip › Additional-File-7/Group_PAU-Hib4/blast2go_hib4_enriched_mf.png]

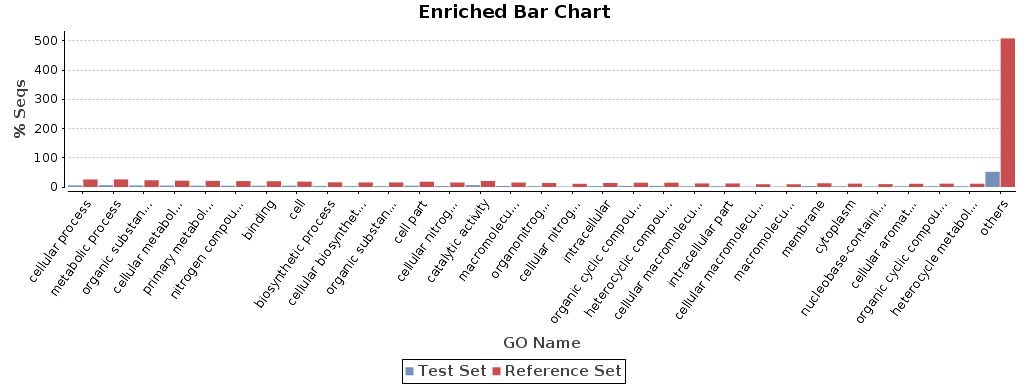

Supplement: Supplementary file 7 — Raw data of the gene ontologies enrichments tests with Blast2GO. (ZIP 22422 kb) [file 12864_2019_5565_MOESM7_ESM.zip › Additional-File-7/Group_PAU-Hib4/blast2go_statistics_hib4.png]

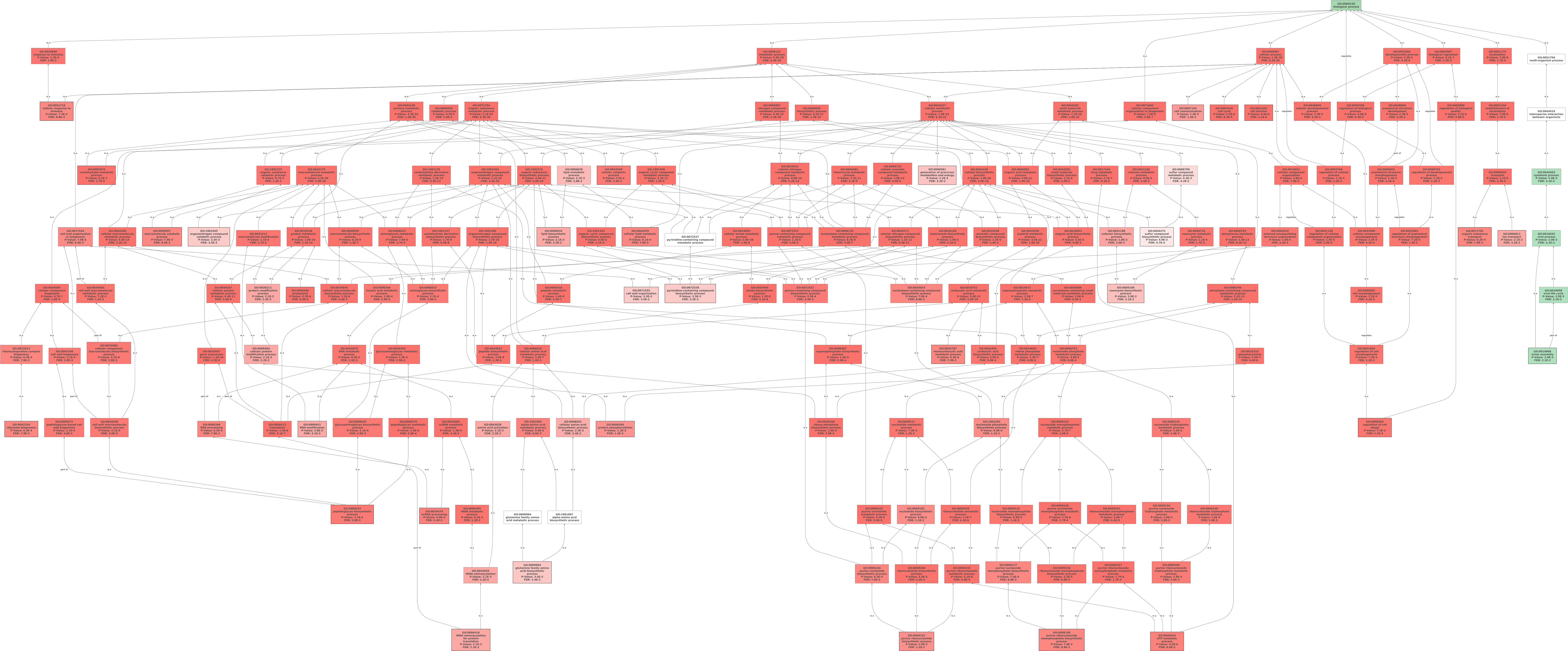

Supplement: Supplementary file 7 — Raw data of the gene ontologies enrichments tests with Blast2GO. (ZIP 22422 kb) [file 12864_2019_5565_MOESM7_ESM.zip › Additional-File-7/Group_PAU/blast2go_PAU_enriched_bp.png]

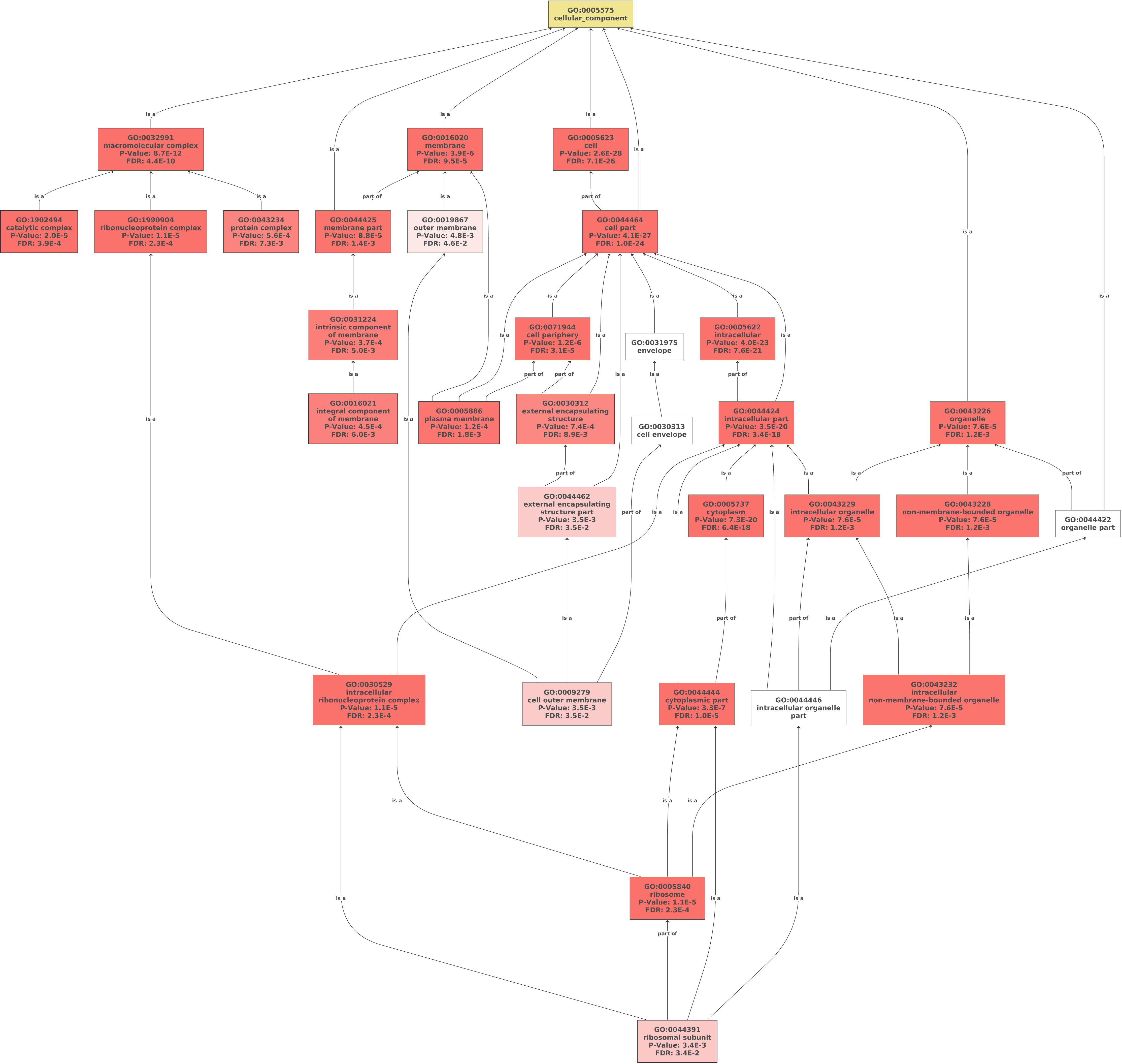

Supplement: Supplementary file 7 — Raw data of the gene ontologies enrichments tests with Blast2GO. (ZIP 22422 kb) [file 12864_2019_5565_MOESM7_ESM.zip › Additional-File-7/Group_PAU/blast2go_PAU_enriched_cc.png]

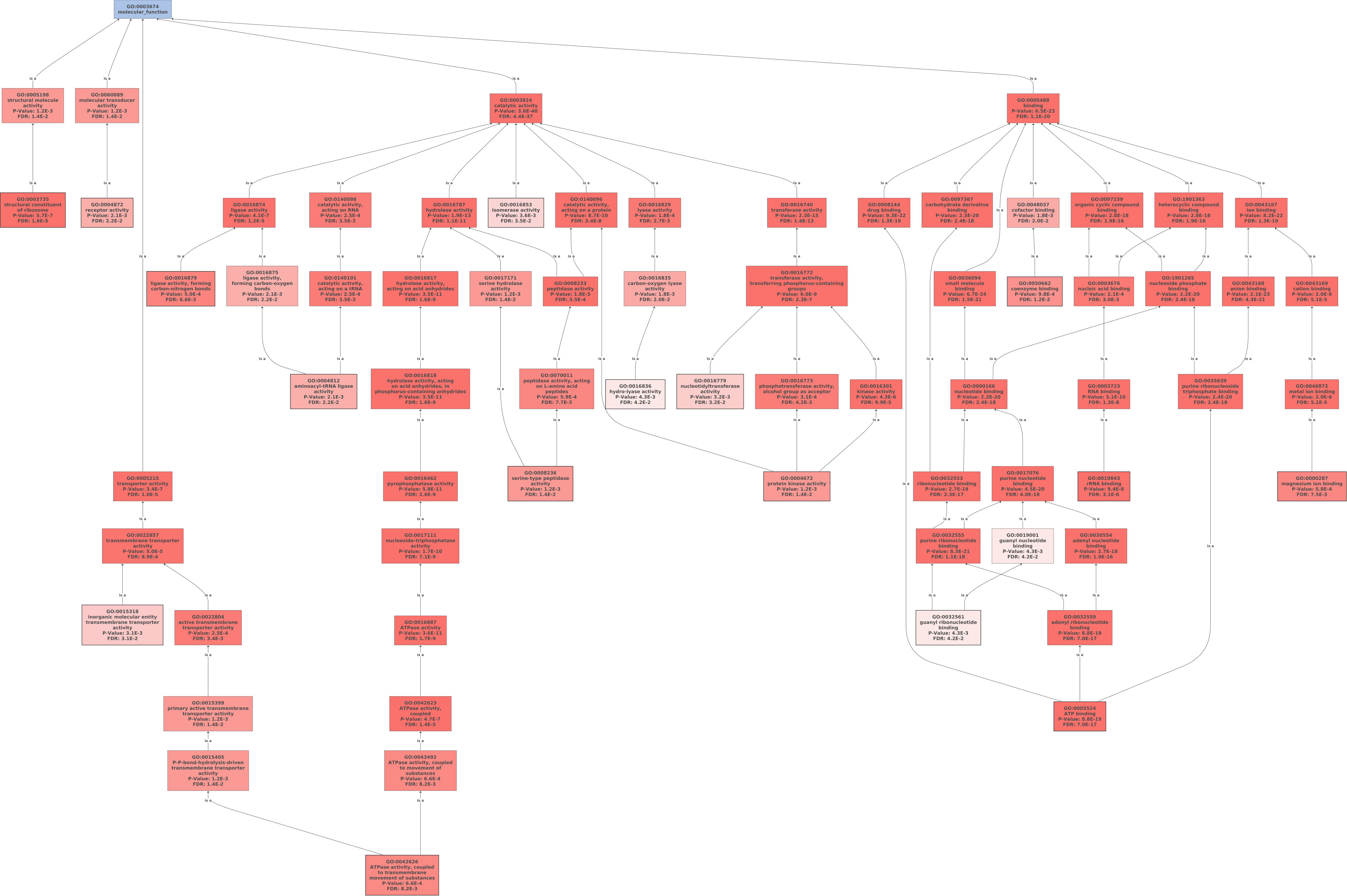

Supplement: Supplementary file 7 — Raw data of the gene ontologies enrichments tests with Blast2GO. (ZIP 22422 kb) [file 12864_2019_5565_MOESM7_ESM.zip › Additional-File-7/Group_PAU/blast2go_PAU_enriched_mf.png]

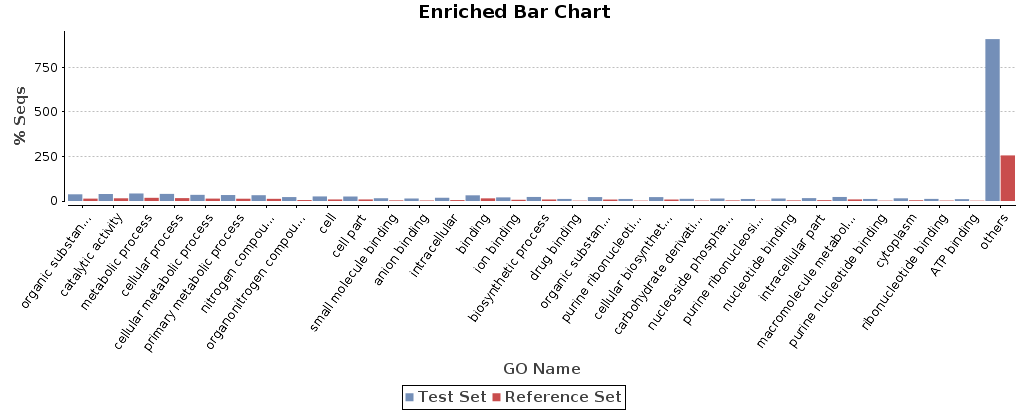

Supplement: Supplementary file 7 — Raw data of the gene ontologies enrichments tests with Blast2GO. (ZIP 22422 kb) [file 12864_2019_5565_MOESM7_ESM.zip › Additional-File-7/Group_PAU/blast2go_statistics_PAU.png]

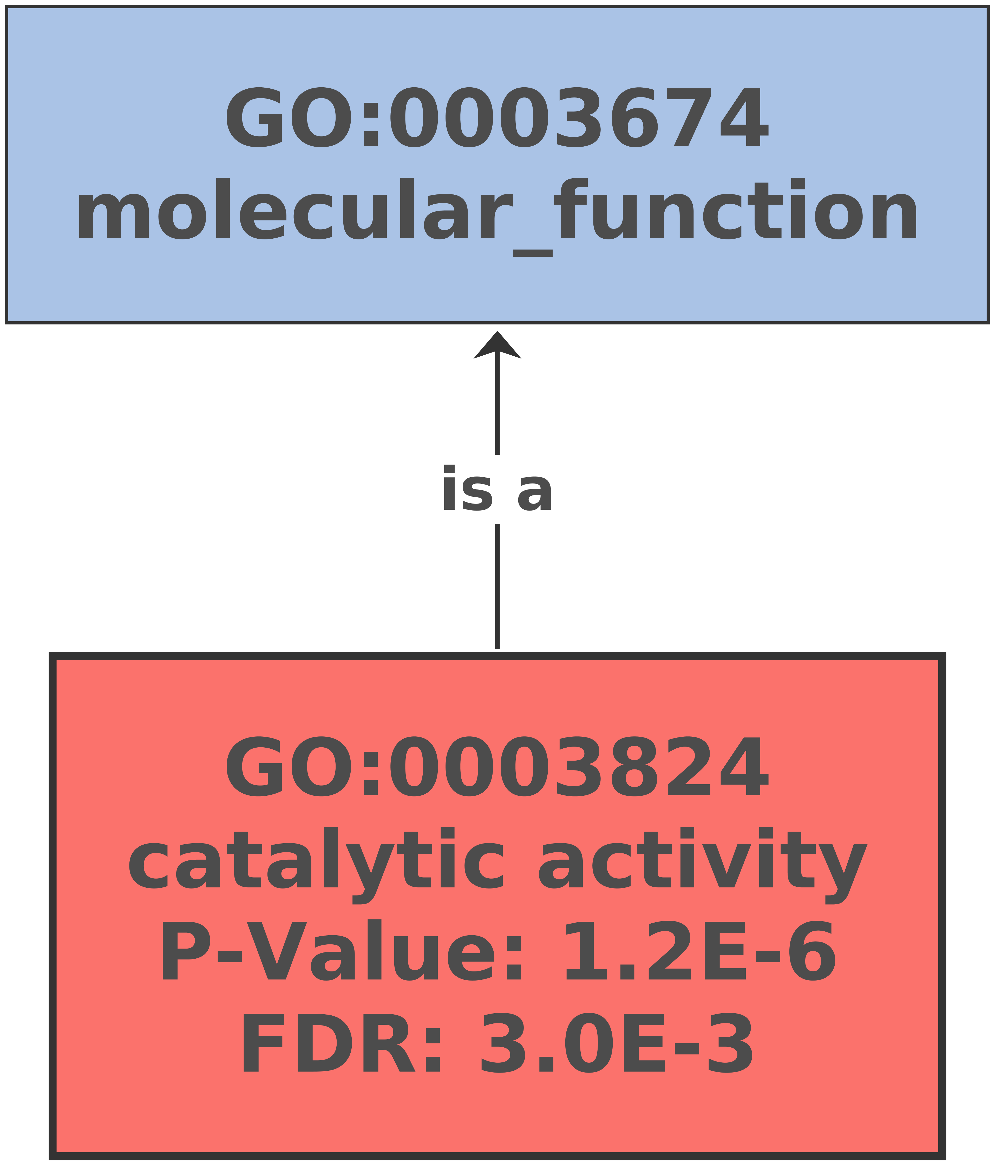

Supplement: Supplementary file 7 — Raw data of the gene ontologies enrichments tests with Blast2GO. (ZIP 22422 kb) [file 12864_2019_5565_MOESM7_ESM.zip › Additional-File-7/Group_PAU_subclade-1/blast2go_PAU_Subclade-1_enriched_mf.png]

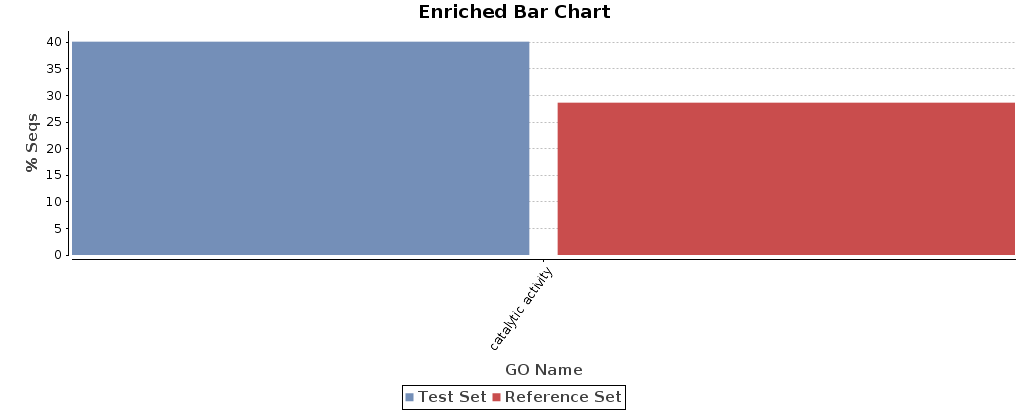

Supplement: Supplementary file 7 — Raw data of the gene ontologies enrichments tests with Blast2GO. (ZIP 22422 kb) [file 12864_2019_5565_MOESM7_ESM.zip › Additional-File-7/Group_PAU_subclade-1/blast2go_statistics_PAU_Subclade-1.png]

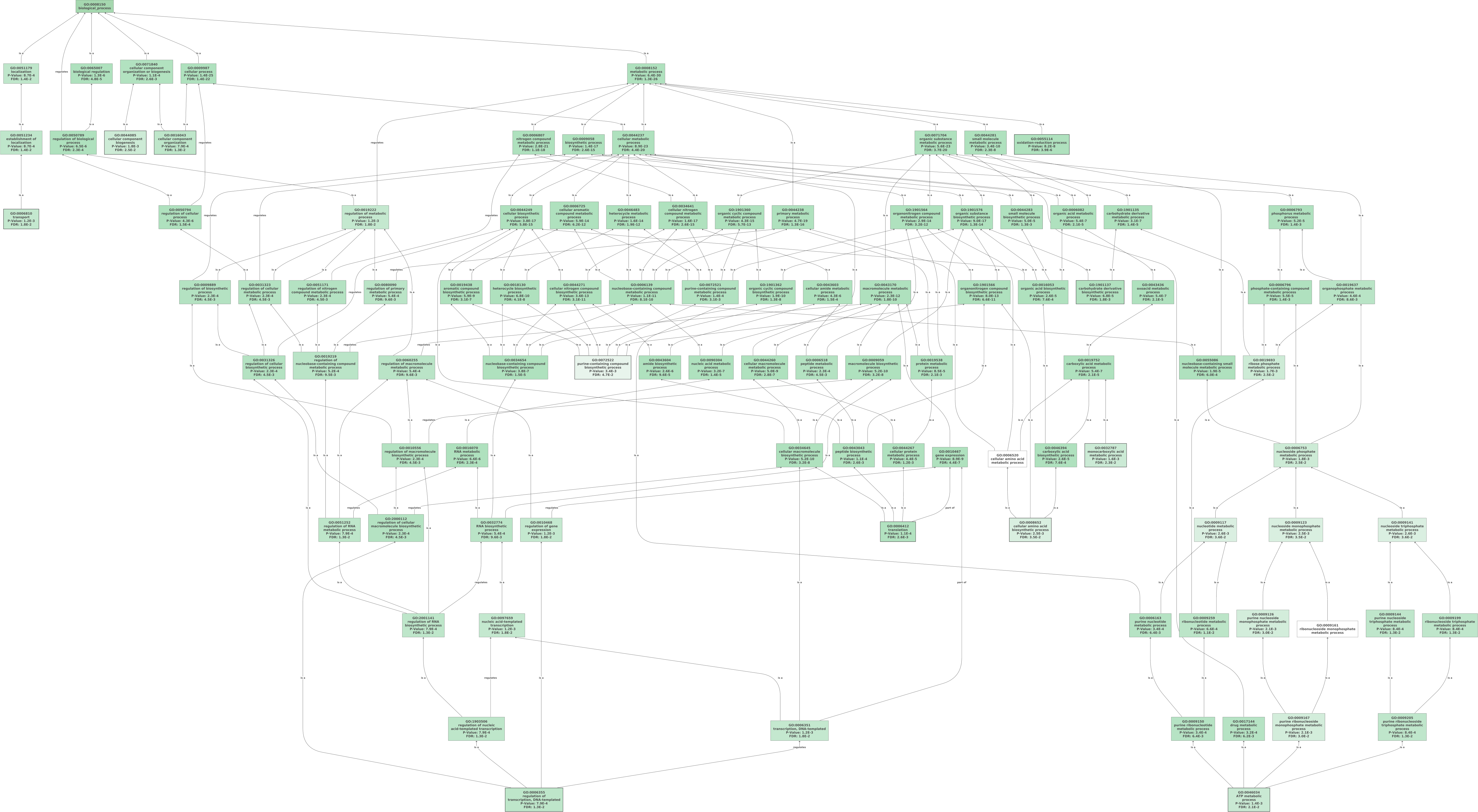

Supplement: Supplementary file 7 — Raw data of the gene ontologies enrichments tests with Blast2GO. (ZIP 22422 kb) [file 12864_2019_5565_MOESM7_ESM.zip › Additional-File-7/Group_PAU_subclade-2/blast2go_PAU_Subclade-2_enriched_bp.png]

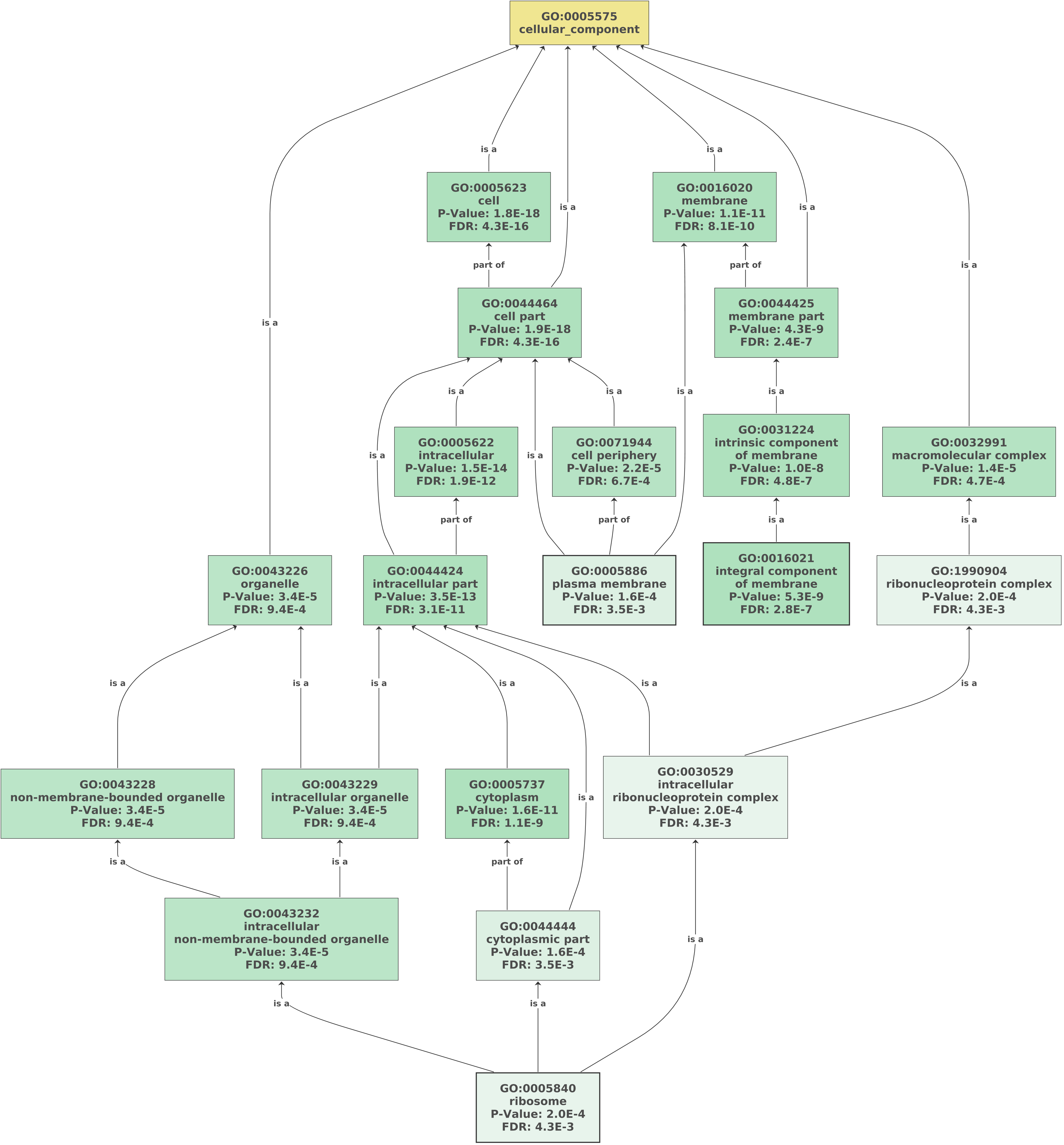

Supplement: Supplementary file 7 — Raw data of the gene ontologies enrichments tests with Blast2GO. (ZIP 22422 kb) [file 12864_2019_5565_MOESM7_ESM.zip › Additional-File-7/Group_PAU_subclade-2/blast2go_PAU_Subclade-2_enriched_cc.png]

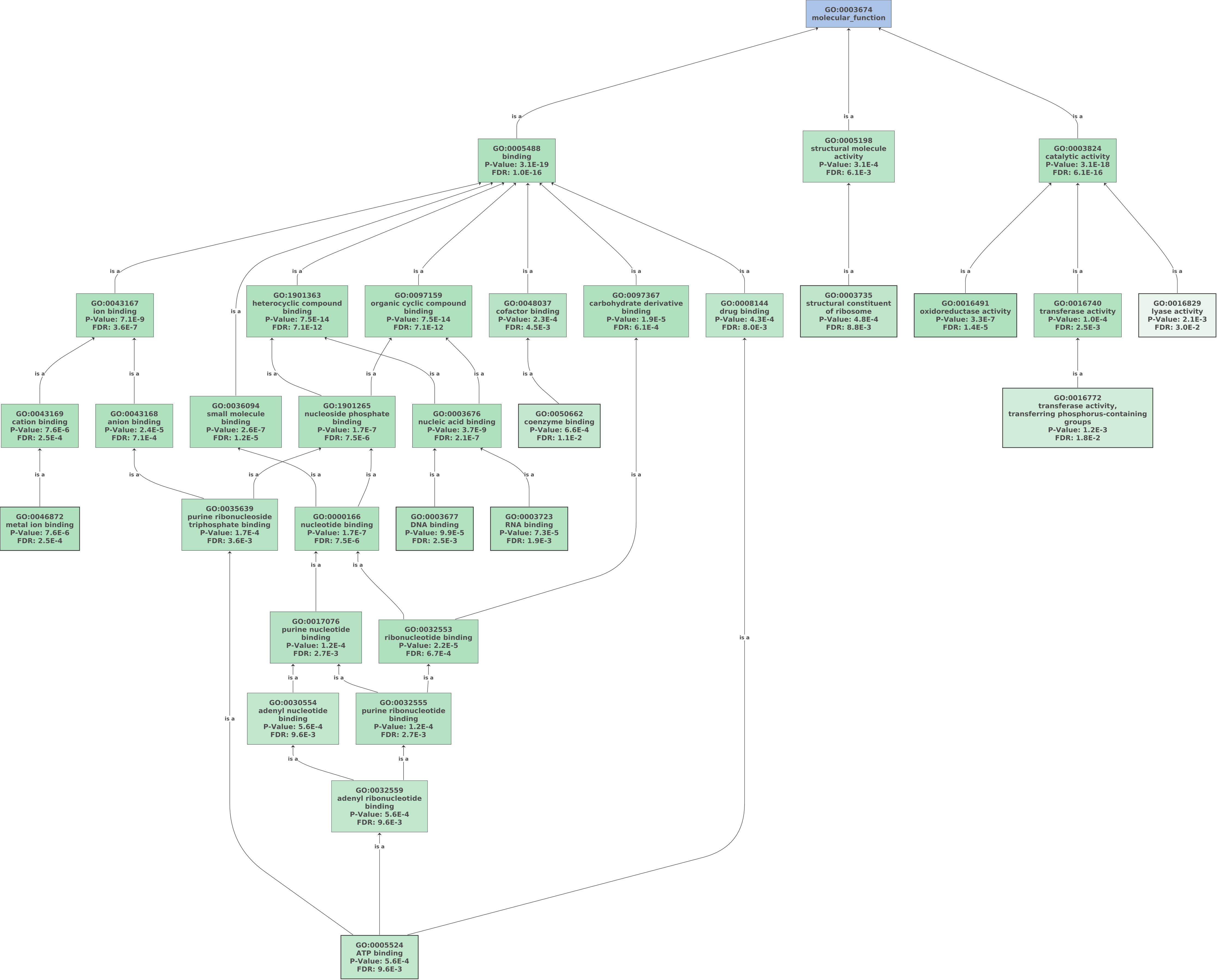

Supplement: Supplementary file 7 — Raw data of the gene ontologies enrichments tests with Blast2GO. (ZIP 22422 kb) [file 12864_2019_5565_MOESM7_ESM.zip › Additional-File-7/Group_PAU_subclade-2/blast2go_PAU_Subclade-2_enriched_mf.png]

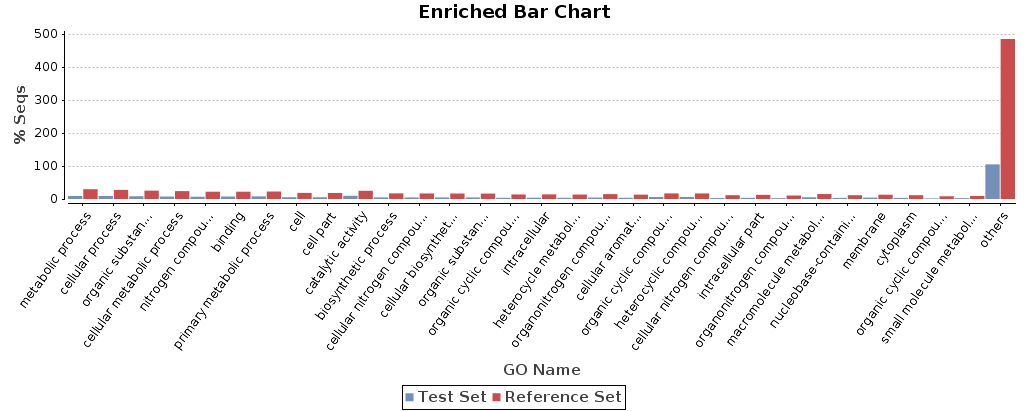

Supplement: Supplementary file 7 — Raw data of the gene ontologies enrichments tests with Blast2GO. (ZIP 22422 kb) [file 12864_2019_5565_MOESM7_ESM.zip › Additional-File-7/Group_PAU_subclade-2/blast2go_statistics_PAU_Subclade-2.png]

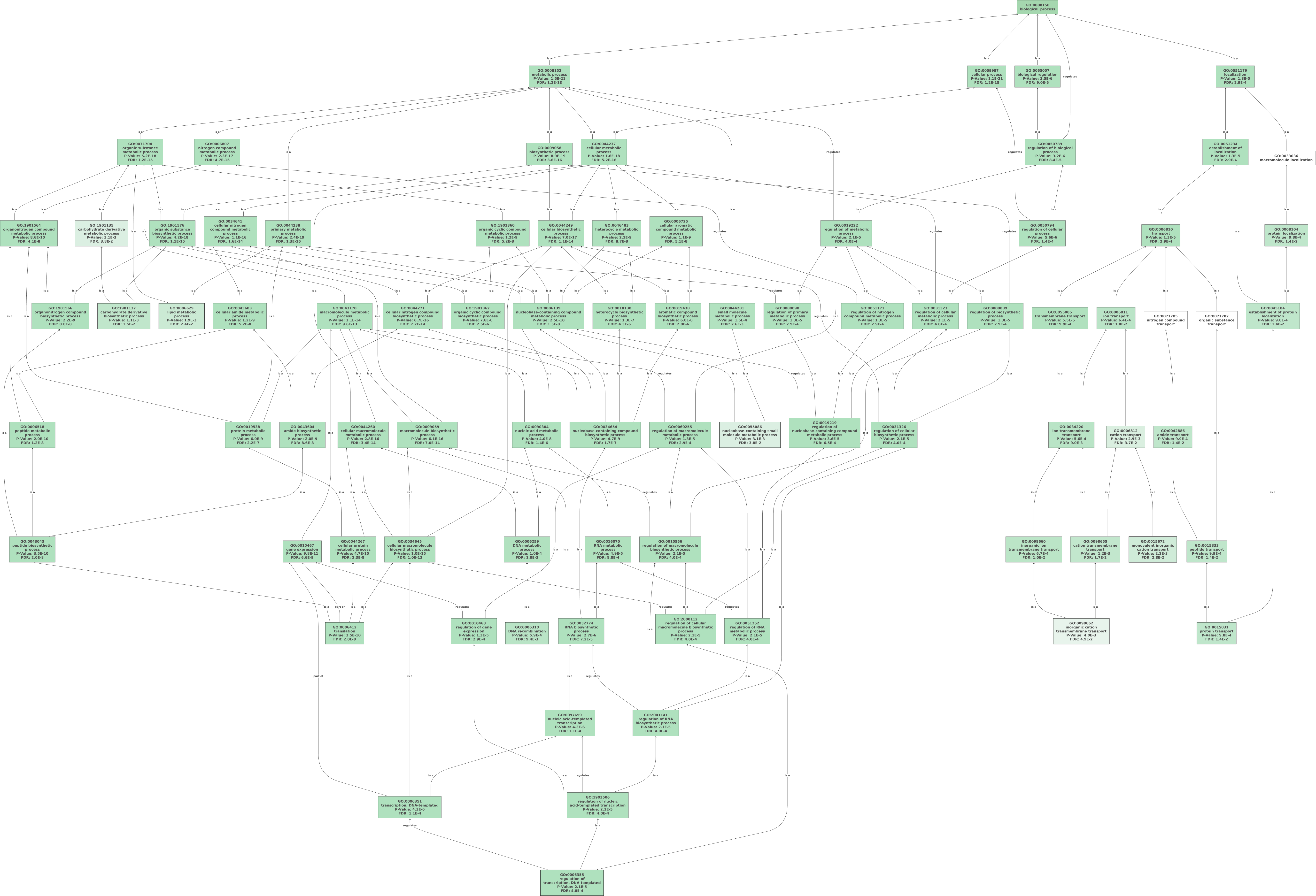

Supplement: Supplementary file 7 — Raw data of the gene ontologies enrichments tests with Blast2GO. (ZIP 22422 kb) [file 12864_2019_5565_MOESM7_ESM.zip › Additional-File-7/Group_PAU_subclade-3/blast2go_PAU_Subclade-3_enriched_bp.png]

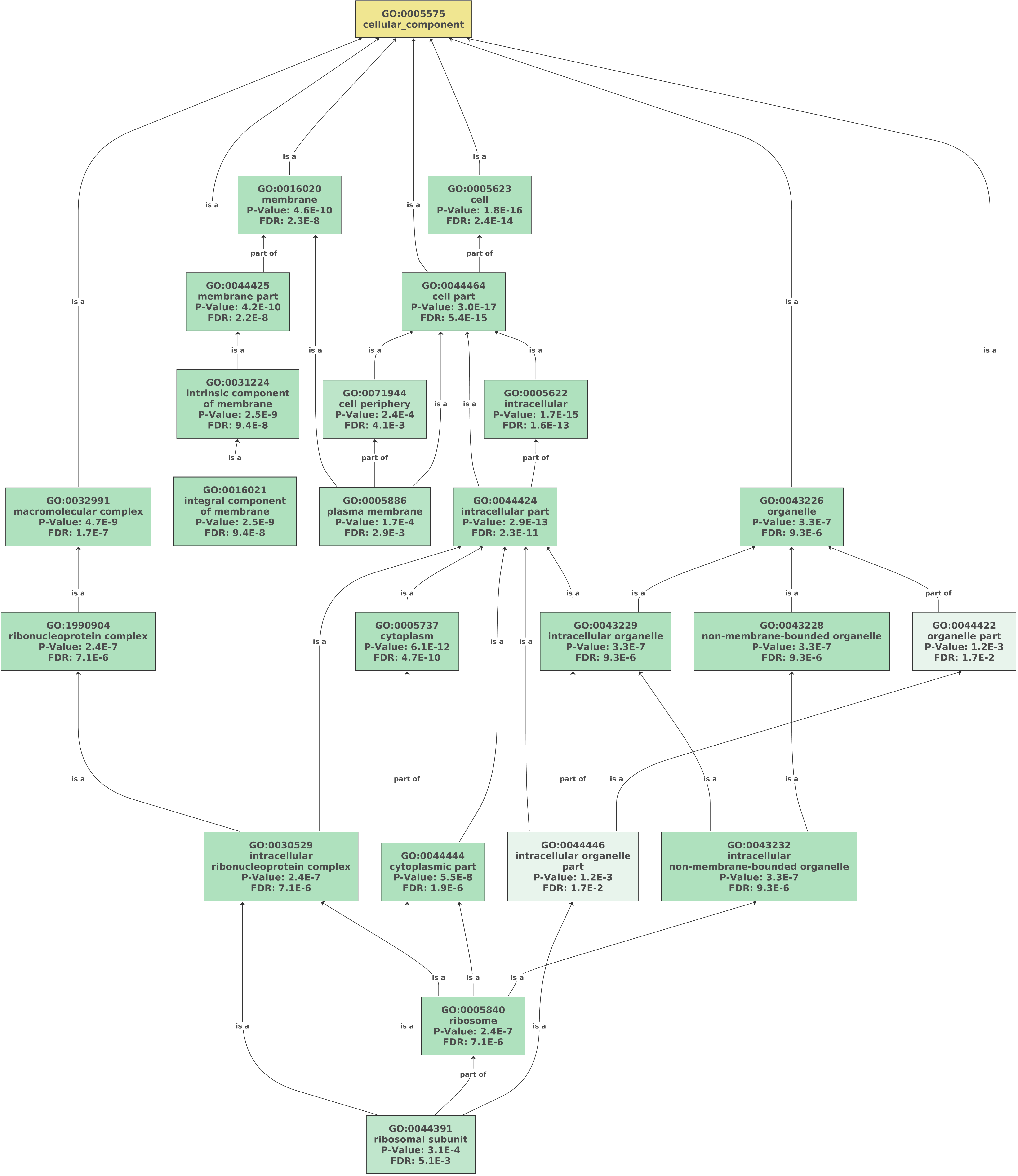

Supplement: Supplementary file 7 — Raw data of the gene ontologies enrichments tests with Blast2GO. (ZIP 22422 kb) [file 12864_2019_5565_MOESM7_ESM.zip › Additional-File-7/Group_PAU_subclade-3/blast2go_PAU_Subclade-3_enriched_cc.png]

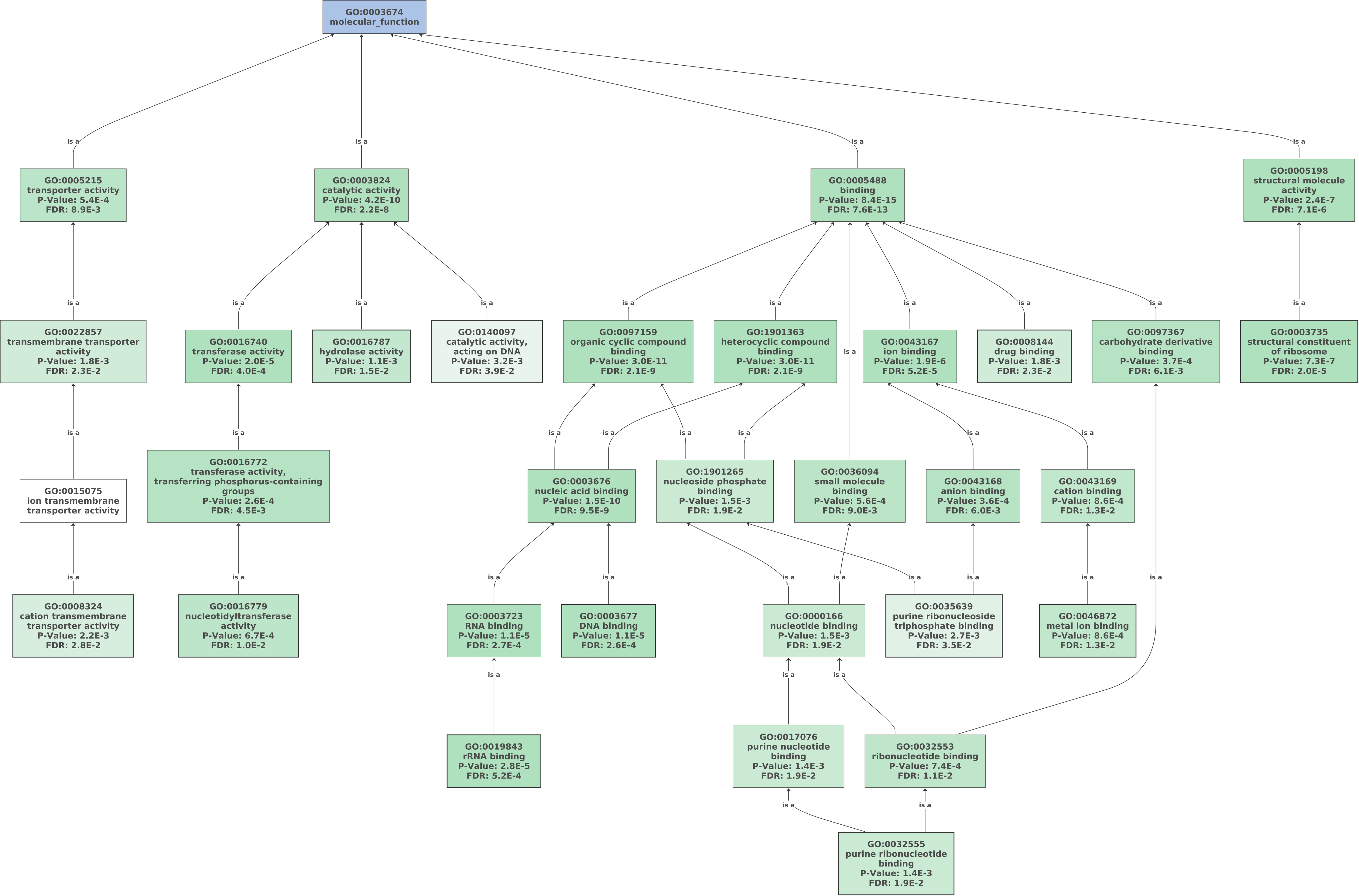

Supplement: Supplementary file 7 — Raw data of the gene ontologies enrichments tests with Blast2GO. (ZIP 22422 kb) [file 12864_2019_5565_MOESM7_ESM.zip › Additional-File-7/Group_PAU_subclade-3/blast2go_PAU_Subclade-3_enriched_mf.png]

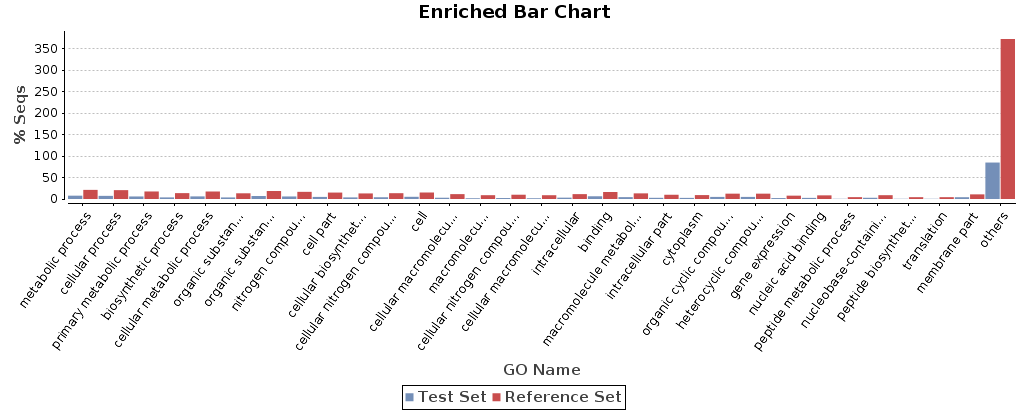

Supplement: Supplementary file 7 — Raw data of the gene ontologies enrichments tests with Blast2GO. (ZIP 22422 kb) [file 12864_2019_5565_MOESM7_ESM.zip › Additional-File-7/Group_PAU_subclade-3/blast2go_statistics_PAU_Subclade-3.png]

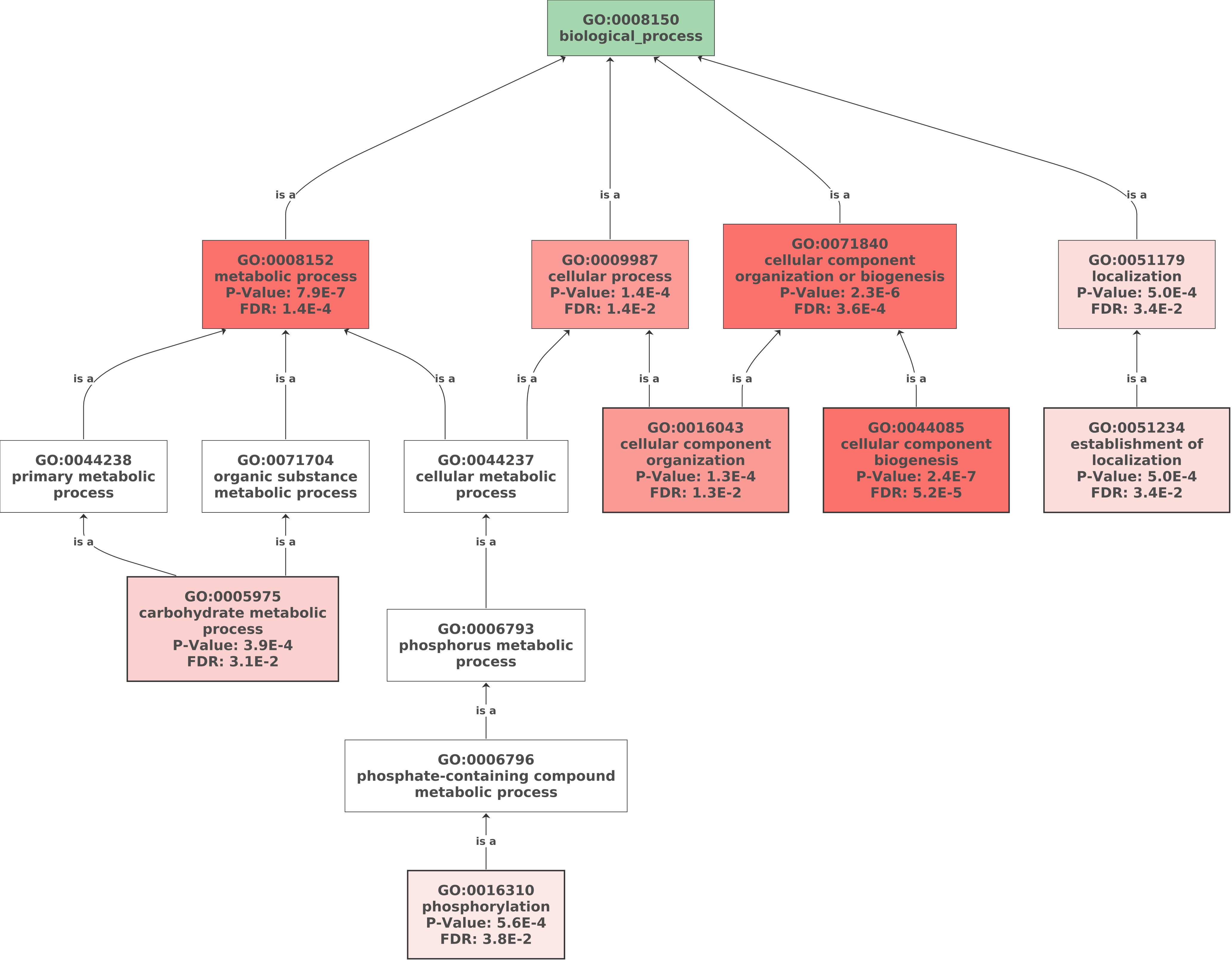

Supplement: Supplementary file 7 — Raw data of the gene ontologies enrichments tests with Blast2GO. (ZIP 22422 kb) [file 12864_2019_5565_MOESM7_ESM.zip › Additional-File-7/Group_SAN2/blast2go_SAN2_enriched_bp.png]

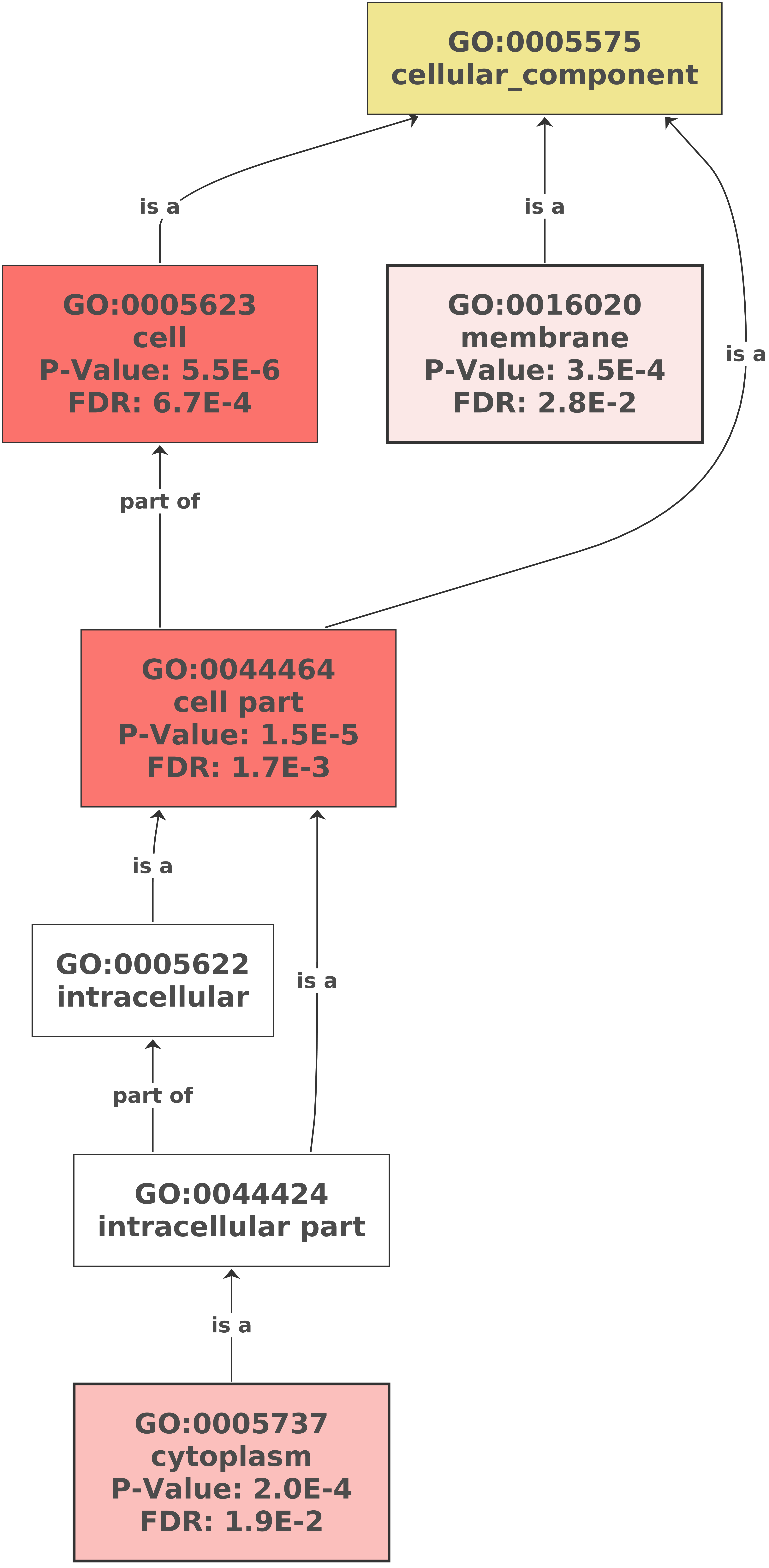

Supplement: Supplementary file 7 — Raw data of the gene ontologies enrichments tests with Blast2GO. (ZIP 22422 kb) [file 12864_2019_5565_MOESM7_ESM.zip › Additional-File-7/Group_SAN2/blast2go_SAN2_enriched_cc.png]

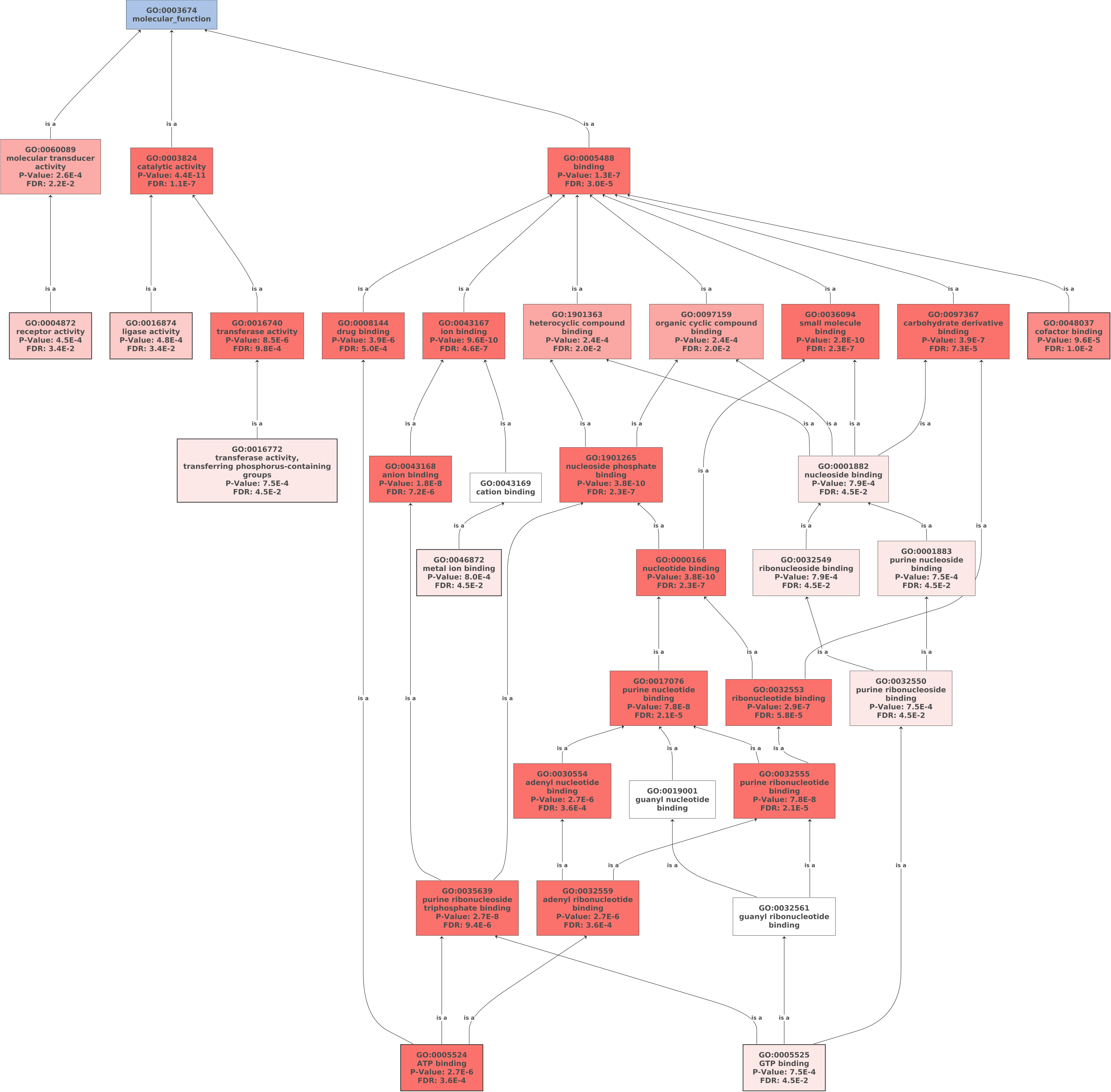

Supplement: Supplementary file 7 — Raw data of the gene ontologies enrichments tests with Blast2GO. (ZIP 22422 kb) [file 12864_2019_5565_MOESM7_ESM.zip › Additional-File-7/Group_SAN2/blast2go_SAN2_enriched_mf.png]

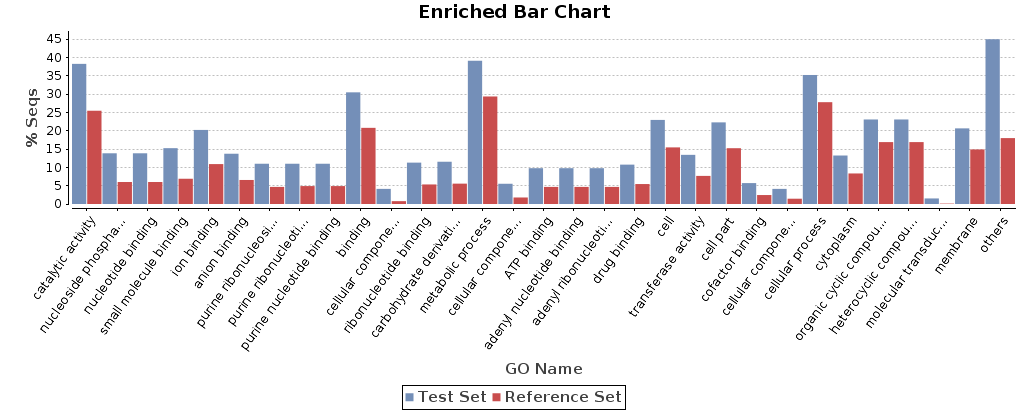

Supplement: Supplementary file 7 — Raw data of the gene ontologies enrichments tests with Blast2GO. (ZIP 22422 kb) [file 12864_2019_5565_MOESM7_ESM.zip › Additional-File-7/Group_SAN2/blast2go_statistics_SAN2.png]

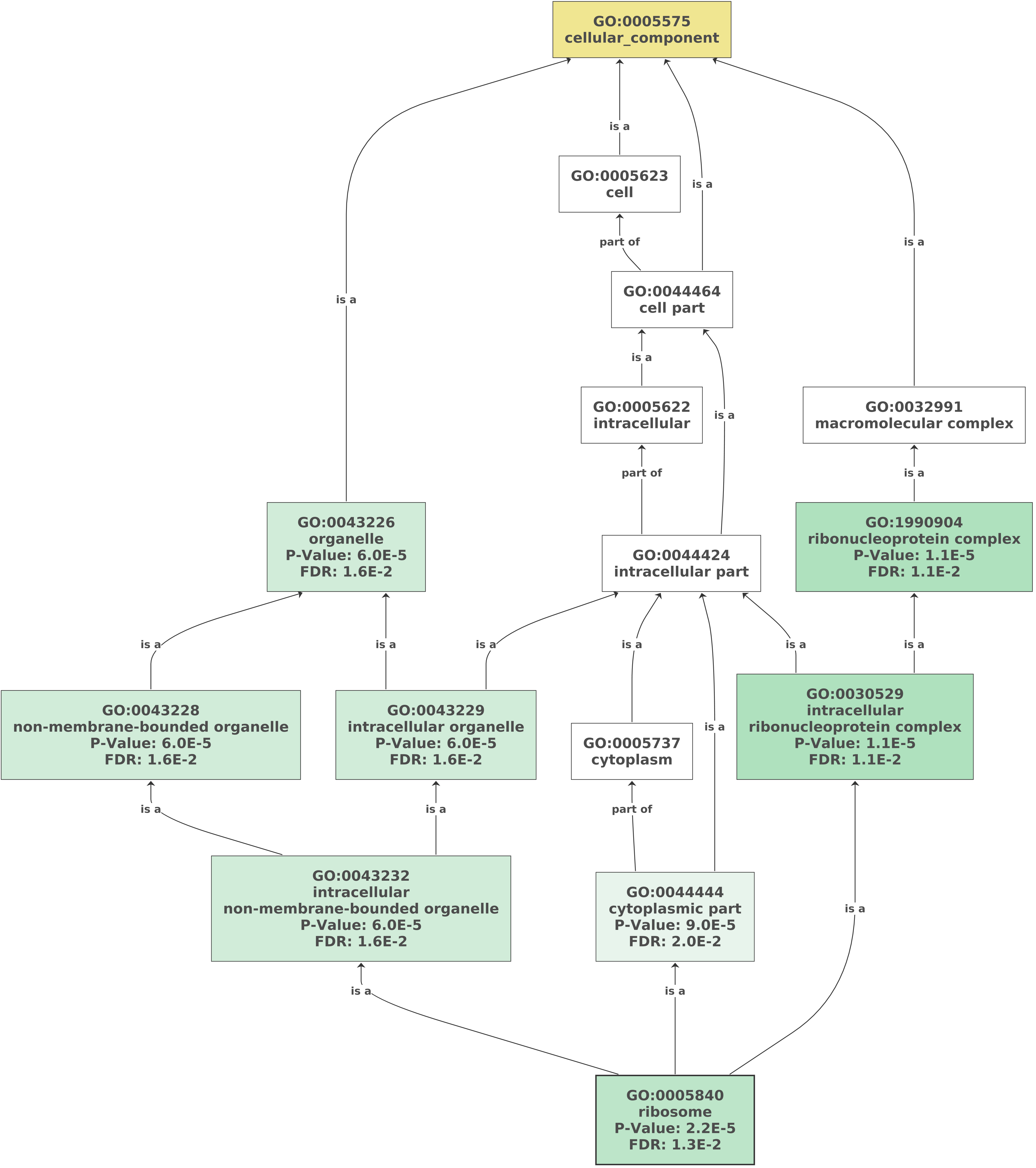

Supplement: Supplementary file 7 — Raw data of the gene ontologies enrichments tests with Blast2GO. (ZIP 22422 kb) [file 12864_2019_5565_MOESM7_ESM.zip › Additional-File-7/Group_SAN/blast2go_SAN_enriched_cc.png]

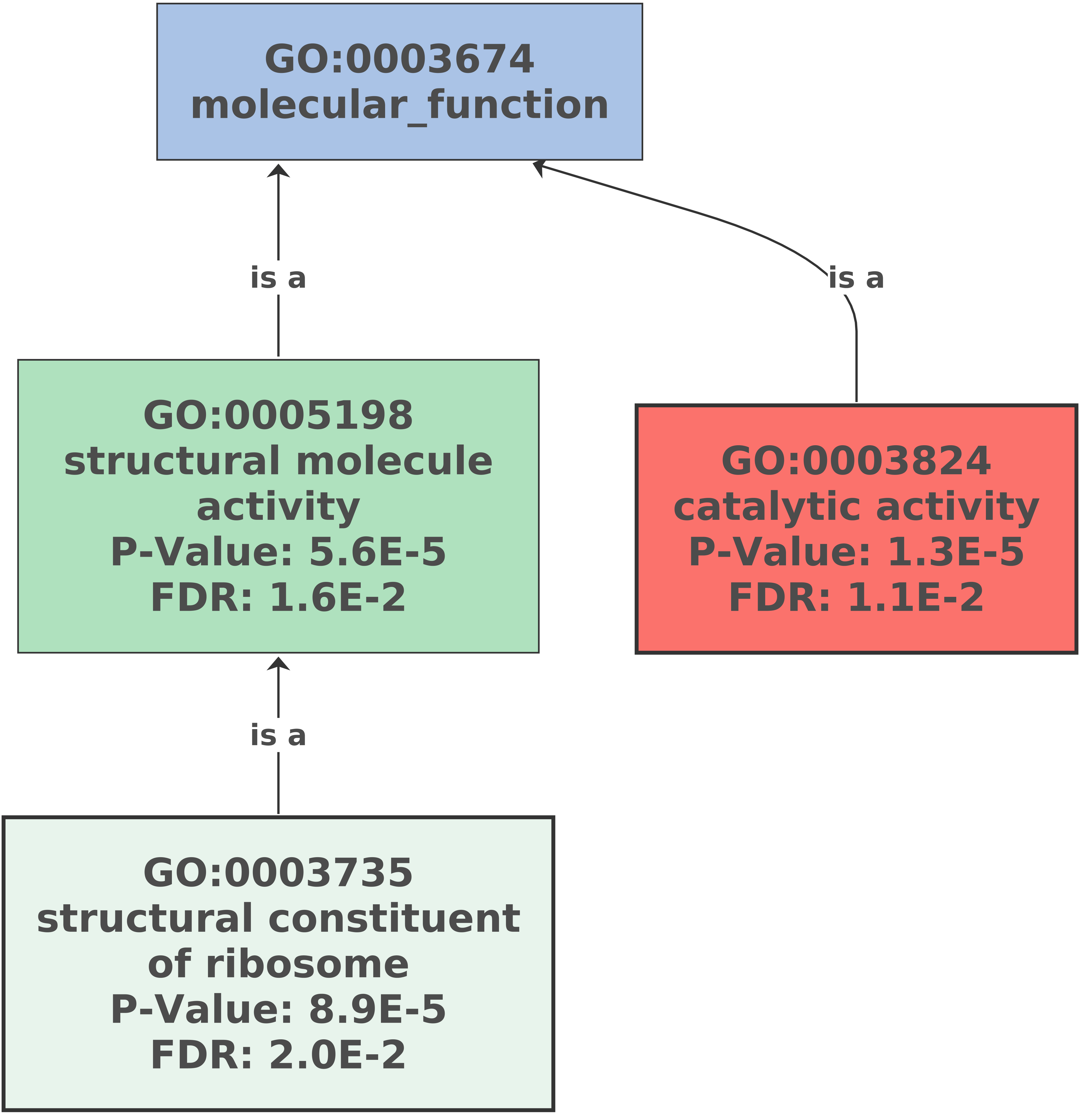

Supplement: Supplementary file 7 — Raw data of the gene ontologies enrichments tests with Blast2GO. (ZIP 22422 kb) [file 12864_2019_5565_MOESM7_ESM.zip › Additional-File-7/Group_SAN/blast2go_SAN_enriched_mf.png]

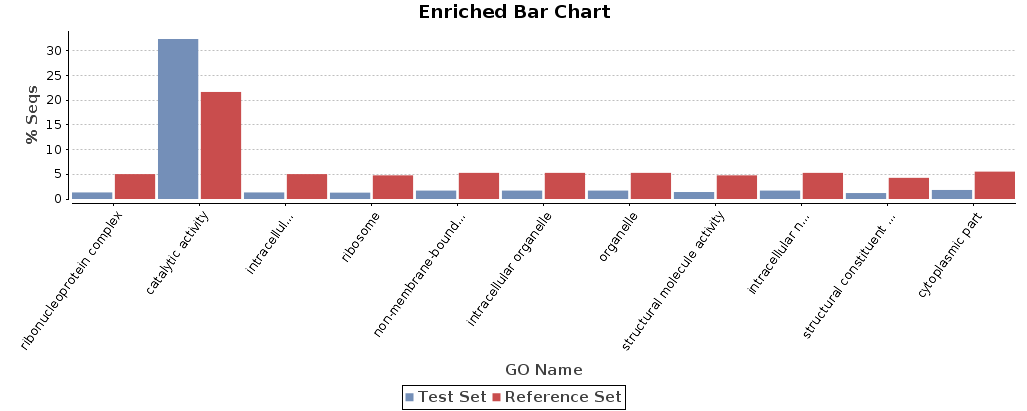

Supplement: Supplementary file 7 — Raw data of the gene ontologies enrichments tests with Blast2GO. (ZIP 22422 kb) [file 12864_2019_5565_MOESM7_ESM.zip › Additional-File-7/Group_SAN/blast2go_statistics_SAN.png]
