# Supplementary material for: Identification of genetic relationships and subspecies signatures in Xylella fastidiosa
Source: BMC Genomics. 2019 Mar 25;20:239. doi: 10.1186/s12864-019-5565-9 (PMC6434890; doi:10.1186/s12864-019-5565-9)
Supplement: Supplementary file 9 — Blast analysis of the three large fragments specific to X. fastidiosa subsp. pauca Hib4 strain. (DOCX 34 kb) [file 12864_2019_5565_MOESM9_ESM.docx]

**Additional File 9.** Blast analysis of the three large fragments specific to *X. fastidiosa* subsp. *pauca* Hib4 strain.

**Query= long-mer4538** (Plasmid CP009886.1_2279_35082; 32,804bp)

**Accession ID Description Identities Gaps Score (bits) e-value**

CP009886.1 **Xylella fastidiosa strain Hib4 plasmid pXF64-HB**, c... 32804/32804 (100%) 0/32804 (0%) 60578 0.0

CP026110.1 Paraburkholderia hospita strain DSM 17164 plasmid ... 5556/6474 (86%) 46/6474 (1%) 6828 0.0

4750/5561 (85%) 15/5561 (0%) 5762 0.0

3680/4183 (88%) 26/4183 (1%) 4915 0.0

3807/4488 (85%) 39/4488 (1%) 4479 0.0

3007/3520 (85%) 33/3520 (1%) 3627 0.0

1180/1398 (84%) 28/1398 (2%) 1349 0.0

988/1211 (82%) 16/1211 (1%) 987 0.0

525/597 (88%) 4/597 (1%) 701 0.0

376/438 (86%) 0/438 (0%) 466 2e-125

37/40 (93%) 0/40 (0%) 58.4 0.015

CP022994.1 Paraburkholderia aromaticivorans strain BN5 plasmi... 5543/6466 (86%) 42/6466 (1%) 6789 0.0

4762/5564 (86%) 15/5564 (0%) 5818 0.0

3712/4186 (89%) 29/4186 (1%) 5077 0.0

3032/3556 (85%) 33/3556 (1%) 3633 0.0

2207/2553 (86%) 9/2553 (0%) 2789 0.0

1183/1407 (84%) 28/1407 (2%) 1332 0.0

989/1214 (81%) 16/1214 (1%) 981 0.0

524/597 (88%) 4/597 (1%) 695 0.0

532/630 (84%) 2/630 (0%) 619 2e-171

379/441 (86% )0/441 (0%) 472 5e-127

36/39 (92%) 0/39 (0%) 56.5 0.055

CP000620.1 Burkholderia vietnamiensis G4 plasmid pBVIE04, com... 5192/6493 (80%) 94/6493 (1%) 4697 0.0

4540/5568 (82%) 45/5568 (1%) 4545 0.0

3537/4505 (79%) 66/4505 (1%) 2896 0.0

2402/2976 (81%) 33/2976 (1%) 2285 0.0

1891/2279 (83%) 11/2279 (0%) 2049 0.0

1545/1846 (84%) 27/1846 (1%) 1716 0.00

919/1181 (78%) 24/1181 (2%) 708 0.0

512/594 (86%) 3/594 (1%) 640 1e-177

CP018464.1 Xanthomonas euvesicatoria strain LMG930 plasmid pL... 4503/5570 (81%) 49/5570 (1%) 4329 0.0

3065/3600 (85%) 9/3600 (0%) 3675 0.0

3566/4497 (79%) 51/4497 (1%) 3099 0.0

2899/3643 (80%) 29/3643 (1%) 2579 0.0

2359/2971 (79%) 27/2971 (1%) 2071 0.0

893/1145 (78%) 26/1145 (2%) 695 0.0

500/591 (85%) 5/591 (1%) 582 2e-160

CP000542.1 Verminephrobacter eiseniae EF01-2, complete genome 943/1062 (89%) 3/1062 (0%) 1299 0.0

CP018408.1 Burkholderia pseudomallei strain 2013833057 chromo... 1167/1549 (75%) 27/1549 (2%) 719 0.0

CP018406.1 Burkholderia pseudomallei strain 2013833055 chromo... 1167/1549 (75%) 27/1549 (2%) 719 0.0

CP018393.1 Burkholderia pseudomallei strain 2011756296 chromo... 1167/1549 (75%) 27/1549 (2%) 719 0.0

CP018368.1 Burkholderia pseudomallei strain 2002721100 chromo... 1167/1549 (75%) 27/1549 (2%) 719 0.0

CP018383.1 Burkholderia pseudomallei strain 2008724758 chromo... 1164/1550 (75%) 29/1550 (2%) 697 0.0

CP009585.1 Burkholderia pseudomallei strain PHLS 112 chromoso... 1161/1557 (75% ) 35/1557 (2%) 649 2e-180

CP003781.1 Burkholderia pseudomallei BPC006 chromosome I, com... 1161/1557 (75%) 35/1557 (2%) 649 2e-180

CP017564.1 Paraburkholderia sprentiae WSM5005 plasmid pl3WSM5 934/1303 (72%) 40/1303 (3%) 326 4e-83

CP000941.1 **Xylella fastidiosa M12, complete genome** 100/106 (94%) 0/106 (0%) 163 3e-34

CP009885.1 **Xylella fastidiosa strain Hib4, complete genome** 97/106 (92%) 6/106 (6%) 141 1e-27

97/106 (92%) 6/106 (6%) 141 1e-27

89/99 (90%) 0/99 (0%) 128 1e-23

CP009823.1 **Xylella fastidiosa strain J1a12, complete genome** 90/99 (91%) 0/99 (0%) 134 2e-25

CP009829.1 **Xylella fastidiosa strain 3124, complete genome** 90/99 (91%) 0/99 (0%) 134 2e-25

CP009790.1 **Xylella fastidiosa strain U24D, complete genome** 90/99 (91%) 0/99 (0%) 134 2e-25

AE003849.1 **Xylella fastidiosa 9a5c, complete genome** 90/99 (91%) 0/99 (0%) 134 2e-25

CP023285.1 Xanthomonas citri subsp. citri strain 03-1638-1-1 ... 102/119 (86%) 0/119 (0%) 126 4e-23

CP020889.1 Xanthomonas citri subsp. citri strain TX160197, co... 102/119 (86%) 0/119 (0%) 126 4e-23

CP020882.1 Xanthomonas citri subsp. citri strain TX160042, co... 102/119 (86%) 0/119 (0%) 126 4e-23

CP020885.1 Xanthomonas citri subsp. citri strain TX160149, co... 102/119 (86%) 0/119 (0%) 126 4e-23

CP011532.1 Xanthomonas oryzae pv. oryzae strain XF89b, comple... 102/119 (86%) 0/119 (0%) 126 4e-23

CP018858.1 Xanthomonas citri subsp. citri strain LH201, compl... 102/119 (86%) 0/119 (0%) 126 4e-23

CP018854.1 Xanthomonas citri subsp. citri strain LH276, compl... 102/119 (86%) 0/119 (0%) 126 4e-23

CP018850.1 Xanthomonas citri subsp. citri strain LJ207-7, com... 102/119 (86%) 0/119 (0%) 126 4e-23

CP018847.1 Xanthomonas citri subsp. citri strain LL074-4, com... 102/119 (86%) 0/119 (0%) 126 4e-23

CP013679.1 Xanthomonas oryzae pv. oryzae strain PXO602, compl... 102/119 (86%) 0/119 (0%) 126 4e-23

CP013678.1 Xanthomonas oryzae pv. oryzae strain PXO563, compl... 102/119 (86%) 0/119 (0%) 126 4e-23

CP013677.1 Xanthomonas oryzae pv. oryzae strain PXO524, compl... 102/119 (86%) 0/119 (0%) 126 4e-23

CP013676.1 Xanthomonas oryzae pv. oryzae strain PXO282, compl... 102/119 (86%) 0/119 (0%) 126 4e-23

CP013675.1 Xanthomonas oryzae pv. oryzae strain PXO236, compl... 102/119 (86%) 0/119 (0%) 126 4e-23

CP013674.1 Xanthomonas oryzae pv. oryzae strain PXO211, compl... 102/119 (86%) 0/119 (0%) 126 4e-23

CP013961.1 Xanthomonas oryzae pv. oryzae strain PXO145, compl... 102/119 (86%) 0/119 (0%) 126 4e-23

CP013670.1 Xanthomonas oryzae pv. oryzae strain PXO71, comple... 102/119 (86%) 0/119 (0%) 126 4e-23

CP011827.2 Xanthomonas citri pv. citri strain jx-6, complete ... 102/119 (86%) 0/119 (0%) 126 4e-23

CP012947.1 Xanthomonas oryzae pv. oryzae strain PXO83, comple... 102/119 (86%) 0/119 (0%) 126 4e-23

CP000967.2 Xanthomonas oryzae pv. oryzae PXO99A, complete genome 102/119 (86%) 0/119 (0%) 126 4e-23

CP008989.1 Xanthomonas citri subsp. citri strain UI7, complet... 102/119 (86%) 0/119 (0%) 126 4e-23

CP008995.1 Xanthomonas citri subsp. citri strain NT17, comple... 102/119 (86%) 0/119 (0%) 126 4e-23

CP009010.1 Xanthomonas citri subsp. citri strain jx5, complet... 102/119 (86%) 0/119 (0%) 126 4e-23

CP009013.1 Xanthomonas citri subsp. citri strain jx4, complet... 102/119 (86%) 0/119 (0%) 126 4e-23

CP009016.1 Xanthomonas citri subsp. citri strain gd3, complet... 102/119 (86%) 0/119 (0%) 126 4e-23

CP009022.1 Xanthomonas citri subsp. citri strain FB19, comple... 102/119 (86%) 0/119 (0%) 126 4e-23

CP009031.1 Xanthomonas citri subsp. citri strain AW13, comple... 102/119 (86%) 0/119 (0%) 126 4e-23

CP008992.1 Xanthomonas citri subsp. citri UI6, complete genome 102/119 (86%) 0/119 (0%) 126 4e-23

CP008998.1 Xanthomonas citri subsp. citri strain MN12, comple... 102/119 (86%) 0/119 (0%) 126 4e-23

CP009001.1 Xanthomonas citri subsp. citri strain MN11, comple... 102/119 (86%) 0/119 (0%) 126 4e-23

CP009004.1 Xanthomonas citri subsp. citri strain MN10, comple... 102/119 (86%) 0/119 (0%) 126 4e-23

CP009007.1 Xanthomonas citri subsp. citri strain mf20, comple... 102/119 (86%) 0/119 (0%) 126 4e-23

CP009019.1 Xanthomonas citri subsp. citri strain gd2, complet... 102/119 (86%) 0/119 (0%) 126 4e-23

CP009025.1 Xanthomonas citri subsp. citri strain BL18, comple... 102/119 (86%) 0/119 (0%) 126 4e-23

CP009028.1 Xanthomonas citri subsp. citri strain 5208, comple... 102/119 (86%) 0/119 (0%) 126 4e-23

CP009034.1 Xanthomonas citri subsp. citri strain AW14, comple... 102/119 (86%) 0/119 (0%) 126 4e-23

CP009037.1 Xanthomonas citri subsp. citri strain AW15, comple... 102/119 (86%) 0/119 (0%) 126 4e-23

CP009040.1 Xanthomonas citri subsp. citri strain AW16, comple... 102/119 (86%) 0/119 (0%) 126 4e-23

CP007166.1 Xanthomonas oryzae pv. oryzae PXO86, complete genome 102/119 (86%) 0/119 (0%) 126 4e-23

CP006857.1 Xanthomonas citri subsp. citri A306, complete genome 102/119 (86%) 0/119 (0%) 126 4e-23

CP003778.1 Xanthomonas citri subsp. citri Aw12879, complete g... 102/119 (86%) 0/119 (0%) 126 4e-23

CP004399.1 Xanthomonas axonopodis Xac29-1, complete genome 102/119 (86%) 0/119 (0%) 126 4e-23

AE008923.1 Xanthomonas axonopodis pv. citri str. 306, complet... 102/119 (86%) 0/119 (0%) 126 4e-23

AP008229.1 Xanthomonas oryzae pv. oryzae MAFF 311018 DNA, com... 102/119 (86%) 0/119 (0%) 126 4e-23

AE013598.1 Xanthomonas oryzae pv. oryzae KACC 10331, complete... 102/119 (86%) 0/119 (0%) 126 4e-23

CP018395.1 Burkholderia pseudomallei strain 2013746776 chromo... 102/119 (86%) 1/119 (1%) 124 1e-22

CP006739.1 **Xylella fastidiosa MUL0034 plasmid unnamed2**, compl... 74/82 (90%) 2/82 (2%) 106 5e-17

CP002166.1 **Xylella fastidiosa subsp. fastidiosa GB514 plasmid**... 74/82 (90%) 2/82 (2%) 106 5e-17

GU938459.1 **Xylella fastidiosa strain Riv19 plasmid pXF-RIV19**,... 74/82 (90%) 2/82 (2%) 106 5e-17

GU938457.1 **Xylella fastidiosa strain Riv11 plasmid pXF-RIV11**,... 74/82 (90%) 2/82 (2%) 106 5e-17

CP018391.1 Burkholderia pseudomallei strain 2011756295 chromo... 59/64 (92%) 0/64 (0%) 91.6 2e-12

AJ416427.1 Plasmid pEMT8 DNA, rep gene for replication initia... 32/33 (97%) 0/33 (0%) 56.5 0.055

**Query= long-mer4596** (Plasmid CP009886.1_48237_64251; 16,015bp)

**Accession ID Description Identities Gaps Score (bits) e-value**

CP009886.1 **Xylella fastidiosa strain Hib4 plasmid pXF64-HB**, c... 16015/16015 (100%) 0/16015 (0%) 29575 0.0

CP026110.1 Paraburkholderia hospita strain DSM 17164 plasmid ... 11984/13852 (87%) 173/13852 (1%) 15071 0.0

CP022994.1 Paraburkholderia aromaticivorans strain BN5 plasmi... 11976/13848 (86%) 171/13848 (1%) 15043 0.0

CP018464.1 Xanthomonas euvesicatoria strain LMG930 plasmid pL... 7890/9799 (81%) 111/9799 (1%) 7417 0.0

1950/2507 (78%) 117/2507 (5%) 1435 0.0

743/931 (80%) 5/931 (1%) 673 0.0

CP000620.1 Burkholderia vietnamiensis G4 plasmid pBVIE04, com... 5813/7095 (82%) 49/7095 (1%) 5954 0.0

1756/2165 (81%) 38/2165 (2%) 1698 0.0

2008/2567 (78%) 98/2567 (4%) 1554 0.0

CP000541.1 Acidovorax sp. JS42 plasmid pAOVO02, complete sequ... 629/706 (89%) 9/706 (1%) 869 0.0

KR071788.1 Xanthomonas oryzae pv. oryzicola strain GX01 plasm... 578/741 (78%) 34/741 (5%) 435 3e-116

CP011963.1 Xanthomonas oryzae pv. oryzicola strain CFBP2286 p... 434/547 (79%) 24/547 (4%) 363 1e-94

CP023285.1 Xanthomonas citri subsp. citri strain 03-1638-1-1 ... 308/392 (79%) 1/392 (0%) 257 7e-63

CP023155.1 Xanthomonas citri subsp. malvacearum strain AR8100... 349/454 (77%) 0/454 (0%) 257 7e-63

CP013004.1 Xanthomonas citri subsp. malvacearum strain XcmH10... 349/454 (77%) 0/454 (0%) 257 7e-63

CP020885.1 Xanthomonas citri subsp. citri strain TX160149, co... 349/454 (77%) 0/454 (0%) 257 7e-63

CP018418.1 Burkholderia pseudomallei strain 2002734728 chromo... 349/454 (77%) 0/454 (0%) 257 7e-63

CP018858.1 Xanthomonas citri subsp. citri strain LH201, compl... 349/454 (77%) 0/454 (0%) 257 7e-63

CP018847.1 Xanthomonas citri subsp. citri strain LL074-4, com... 349/454 (77%) 0/454 (0%) 257 7e-63

CP018399.1 Burkholderia pseudomallei strain 2013746777 chromo... 307/391 (79%) 0/391 (0%) 257 7e-63

CP013006.1 Xanthomonas citri subsp. malvacearum strain XcmN10... 307/391 (79%) 0/391 (0%) 257 7e-63

CP018854.1 Xanthomonas citri subsp. citri strain LH276, compl... 307/391 (79%) 0/391 (0%) 257 7e-63

CP018850.1 Xanthomonas citri subsp. citri strain LJ207-7, com... 307/391 (79%) 0/391 (0%) 257 7e-63

CP011827.2 Xanthomonas citri pv. citri strain jx-6, complete ... 307/391 (79%) 0/391 (0%) 257 7e-63

CP008989.1 Xanthomonas citri subsp. citri strain UI7, complet... 307/391 (79%) 0/391 (0%) 257 7e-63

CP008995.1 Xanthomonas citri subsp. citri strain NT17, comple... 307/391 (79%) 0/391 (0%) 257 7e-63

CP009010.1 Xanthomonas citri subsp. citri strain jx5, complet... 307/391 (79%) 0/391 (0%) 257 7e-63

CP009013.1 Xanthomonas citri subsp. citri strain jx4, complet... 307/391 (79%) 0/391 (0%) 257 7e-63

CP009016.1 Xanthomonas citri subsp. citri strain gd3, complet... 307/391 (79%) 0/391 (0%) 257 7e-63

CP009022.1 Xanthomonas citri subsp. citri strain FB19, comple... 307/391 (79%) 0/391 (0%) 257 7e-63

CP008992.1 Xanthomonas citri subsp. citri UI6, complete genome 307/391 (79%) 0/391 (0%) 257 7e-63

CP008998.1 Xanthomonas citri subsp. citri strain MN12, comple... 307/391 (79%) 0/391 (0%) 257 7e-63

CP009001.1 Xanthomonas citri subsp. citri strain MN11, comple... 307/391 (79%) 0/391 (0%) 257 7e-63

CP009004.1 Xanthomonas citri subsp. citri strain MN10, comple... 307/391 (79%) 0/391 (0%) 257 7e-63

CP009007.1 Xanthomonas citri subsp. citri strain mf20, comple... 307/391 (79%) 0/391 (0%) 257 7e-63

CP009019.1 Xanthomonas citri subsp. citri strain gd2, complet... 307/391 (79%) 0/391 (0%) 257 7e-63

CP009025.1 Xanthomonas citri subsp. citri strain BL18, comple... 307/391 (79%) 0/391 (0%) 257 7e-63

CP009028.1 Xanthomonas citri subsp. citri strain 5208, comple... 307/391 (79%) 0/391 (0%) 257 7e-63

CP006857.1 Xanthomonas citri subsp. citri A306, complete genome 307/391 (79%) 0/391 (0%) 257 7e-63

CP004399.1 Xanthomonas axonopodis Xac29-1, complete genome 307/391 (79%) 0/391 (0%) 257 7e-63

AE008923.1 Xanthomonas axonopodis pv. citri str. 306, complet... 307/391 (79%) 0/391 (0%) 257 7e-63

CP018395.1 Burkholderia pseudomallei strain 2013746776 chromo... 306/391 (78%) 0/391 (0%) 252 3e-61

CP020889.1 Xanthomonas citri subsp. citri strain TX160197, co... 292/370 (79%) 0/370 (0%) 252 3e-61

CP020882.1 Xanthomonas citri subsp. citri strain TX160042, co... 292/370 (79%) 0/370 (0%) 252 3e-61

CP009031.1 Xanthomonas citri subsp. citri strain AW13, comple... 290/367 (79%) 0/367 (0%) 252 3e-61

CP009034.1 Xanthomonas citri subsp. citri strain AW14, comple... 290/367 (79%) 0/367 (0%) 252 3e-61

CP009037.1 Xanthomonas citri subsp. citri strain AW15, comple... 290/367 (79%) 0/367 (0%) 252 3e-61

CP009040.1 Xanthomonas citri subsp. citri strain AW16, comple... 290/367 (79%) 0/367 (0%) 252 3e-61

CP003778.1 Xanthomonas citri subsp. citri Aw12879, complete g... 290/367 (79%) 0/367 (0%) 252 3e-61

CP022008.1 Burkholderia gladioli pv. gladioli strain KACC 118... 164/198 (83%) 6/198 (3%) 172 3e-37

CP009319.1 Burkholderia gladioli strain ATCC 10248 plasmid 3,... 164/198 (83%) 6/198 (3%) 172 3e-37

GQ401131.1 Burkholderia pseudomallei plasmid pPHB194, complet... 164/198 (83%) 6/198 (3%) 172 3e-37

CP022009.1 Burkholderia gladioli pv. gladioli strain KACC 118... 163/198 (82%) 7/198 (4%) 165 4e-35

CP013424.1 Burkholderia sp. MSMB0852 chromosome 1, complete s... 168/208 (81%) 8/208 (4%) 156 3e-32

**Query= long-mer2422** (Chromosome CP009885.1_1436207_1470354; 34,148bp)

**Accession ID Description Identities Gaps Score (bits) e-value**

CP009885.1 **Xylella fastidiosa strain Hib4, complete genome** 34148/34148 (100%) 0/34148 63060 0.0

CP017749.1 Cupriavidus sp. USMAA2-4 chromosome 2, complete se... 17228/18075 (95%) 42/18075 (0%) 28648 0.0

LN879547.1 Comamonas testosteroni P19 genome assembly Comamon... 18497/20407 (91%) 91/20407 (0%) 27019 0.0

CP027174.1 Pseudomonas aeruginosa strain AR_0230 chromosome, ... 16796/18135 (93%) 118/18135 (1%) 25963 0.0

CP017293.1 Pseudomonas aeruginosa strain PA83, complete genome 16796/18135 (93%) 118/18135 (1%) 25963 0.0

CP012901.1 Pseudomonas aeruginosa strain N15-01092, complete ... 16796/18135 (93%) 118/18135 (1%) 25963 0.0

CP027172.1 Pseudomonas aeruginosa strain AR_0353 chromosome, ... 18280/20389 (90%) 96/20389 (0%) 25880 0.0

CP021775.1 Pseudomonas aeruginosa strain Pa58, complete genome 18280/20389 (90%) 96/20389 (0%) 25880 0.0

CP018454.1 Klebsiella pneumoniae strain SWU01, complete genome 3721/4300 (87%) 41/4300 (1%) 25874 0.0

CP013993.1 Pseudomonas aeruginosa DHS01, complete genome 18266/20388 (90%) 94/20388 (0%) 25808 0.0

AM902716.1 Bordetella petrii strain DSM 12804, complete genome 17838/19879 (90%) 140/19879 (1%) 25274 0.0

HG916765.1 Castellaniella defragrans 65Phen complete genome 14077/14695 (96%) 35/14695 (0%) 23680 0.0

AP017302.1 Pseudomonas aeruginosa DNA, complete genome, strai... 13716/14496 (95%) 52/14496 (0%) 22400 0.0

CP017190.1 Xanthomonas campestris pv. vesicatoria str. 85-10,... 13742/14652 (94%) 56/14652 (0%) 21965 0.0

AM039952.1 Xanthomonas campestris pv. vesicatoria complete ge... 13742/14652 (94%) 56/14652 (0%) 21965 0.0

CP027166.1 Pseudomonas aeruginosa strain AR_0357 chromosome, ... 15148/16982 (89%) 85/16982 (1%) 21121 0.0

CP027171.1 Pseudomonas aeruginosa strain AR_0354 chromosome, ... 15147/16982 (89%) 85/16982 (1%) 21115 0.0

CP000539.1 Acidovorax sp. JS42, complete genome 13413/14661 (91%) 70/14661 (0%) 20096 0.0

CP011077.1 Klebsiella michiganensis strain RC10, complete genome 13573/15224 (89%) 89/15224 (1%) 18884 0.0

CP021648.1 Acidovorax sp. T1, complete genome 13172/14728 (89%) 79/14728 (1%) 18504 0.0

CP025298.1 Stenotrophomonas maltophilia strain CSM2 chromosom... 13154/14710 (89%) 73/14710 (0%) 18476 0.0

KY852375.1 Pseudomonas aeruginosa tRNA-Glu and tRNA-Gly genes... 13154/14710 (89%) 73/14710 (0%) 18476 0.0

LT969520.1 Pseudomonas aeruginosa isolate RW109 genome assemb... 13153/14710 (89%) 73/14710 (0%) 18471 0.0

CP021380.1 Pseudomonas aeruginosa strain CCBH4851 genome 13153/14710 (89%) 73/14710 (0%) 18471 0.0

CP014999.1 Pseudomonas aeruginosa strain PA7790, complete genome 13153/14710 (89%) 73/14710 (0%) 18471 0.0

CP015003.1 Pseudomonas aeruginosa strain PA11803, complete ge... 13153/14710 (89%) 73/14710 (0%) 18471 0.0

CP015002.1 Pseudomonas aeruginosa strain PA8281, complete genome 13153/14710 (89%) 73/14710 (0%) 18471 0.0

CP015001.1 Pseudomonas aeruginosa strain PA1088, complete genome 13153/14710 (89%) 73/14710 (0%) 18471 0.0

KT454971.1 Pseudomonas aeruginosa strain CCBH4851 genomic seq... 13153/14710 (89%) 73/14710 (0%) 18471 0.0

CP012900.1 Stenotrophomonas acidaminiphila strain ZAC14D2_NAI... 13135/14741 (89%) 128/14741 (1%) 18207 0.0

CP002449.1 Alicycliphilus denitrificans BC, complete genome 13134/14741 (89%) 128/14741 (1%) 18201 0.0

CP008739.2 Pseudomonas aeruginosa VRFPA04, complete genome 10934/11523 (95%) 61/11523 (1%) 17959 0.0

CP016022.1 Ralstonia insidiosa strain ATCC 49129 chromosome 1... 12760/14532 (88%) 117/14532 (1%) 16910 0.0

AP012280.1 Pseudomonas aeruginosa NCGM2.S1 DNA, complete genome 12785/14585 (88%) 158/14585 (1%) 16816 0.0

CP002622.1 Pseudomonas stutzeri DSM 4166, complete genome 12788/14617 (87%) 198/14617 (1%) 16678 0.0

CP000884.1 Delftia acidovorans SPH-1, complete genome 12782/14613 (87%) 196/14613 (1%) 16661 0.0

CP002287.1 Achromobacter xylosoxidans A8, complete genome 12706/14522 (87%) 106/14522 (1%) 16659 0.0

CP017420.1 Delftia tsuruhatensis strain CM13, complete genome 12705/14522 (87%) 106/14522 (1%) 16654 0.0

CP008857.1 Pseudomonas aeruginosa strain F30658, complete genome 12701/14516 (87%) 106/14516 (1%) 16654 0.0

CP017748.1 Cupriavidus sp. USMAA2-4 chromosome 1, complete se... 12688/14581 (87%) 142/14581 (1%) 16308 0.0

CP000774.1 Parvibaculum lavamentivorans DS-1, complete genome 12751/14712 (87%) 108/14712 (1%) 16205 0.0

CP012251.1 Xanthomonas arboricola pv. juglandis strain Xaj 41... 12603/14736 (86%) 169/14736 (1%) 15239 0.0

CP022002.1 Pseudomonas aeruginosa strain Pa1242, complete genome 9106/9529 (96%) 24/9529 (0%) 15232 0.0

CP025750.1 Xanthomonas campestris pv. campestris strain 3811 ... 12593/14733 (85%) 165/14733 (1%) 15199 0.0

CP017308.1 Xanthomonas campestris pv. campestris str. CN03 ch... 12593/14733 (85%) 166/14733 (1%) 15199 0.0

AM920689.1 Xanthomonas campestris pv. campestris complete gen... 12593/14733 (85%) 165/14733 (1%) 15199 0.0

CP020603.1 Pseudomonas aeruginosa strain E6130952, complete g... 10589/11858 (89%) 66/11858 (1%) 14807 0.0

AP014646.1 Pseudomonas aeruginosa DNA, complete genome, strai... 10589/11858 (89%) 66/11858 (1%) 14807 0.0

AP014622.1 Pseudomonas aeruginosa DNA, complete genome, strai... 10589/11858 (89%) 66/11858 (1%) 14807 0.0

CP022263.1 Xanthomonas citri pv. vignicola strain CFBP7111, c... 12536/14769 (85%) 194/14769 (1%) 14724 0.0

CP020889.1 Xanthomonas citri subsp. citri strain TX160197, co... 12534/14769 (85%) 197/14769 (1%) 14709 0.0

CP020882.1 Xanthomonas citri subsp. citri strain TX160042, co... 12534/14769 (85%) 197/14769 (1%) 14709 0.0

CP020885.1 Xanthomonas citri subsp. citri strain TX160149, co... 12534/14769 (85%) 197/14769 (1%) 14709 0.0

CP009031.1 Xanthomonas citri subsp. citri strain AW13, comple... 12534/14769 (85%) 197/14769 (1%) 14709 0.0

CP009037.1 Xanthomonas citri subsp. citri strain AW15, comple... 12534/14769 (85%) 197/14769 (1%) 14709 0.0

CP003778.1 Xanthomonas citri subsp. citri Aw12879, complete g... 12534/14769 (85%) 197/14769 (1%) 14709 0.0

CP009040.1 Xanthomonas citri subsp. citri strain AW16, comple... 12534/14770 (85%) 198/14770 (1%) 14705 0.0

CP009034.1 Xanthomonas citri subsp. citri strain AW14, comple... 12533/14771 (85%) 199/14771 (1%) 14694 0.0

CP020985.1 Xanthomonas citri pv. phaseoli var. fuscans strain... 12540/14789 (85%) 222/14789 (2%) 14646 0.0

CP015972.1 Xanthomonas citri pv. glycines str. 12-2, complete... 12507/14743 (85%) 210/14743 (1%) 14645 0.0

CP021012.1 Xanthomonas citri pv. phaseoli var. fuscans strain... 12538/14789 (85%) 224/14789 (2%) 14633 0.0

CP017188.1 Xanthomonas citri pv. glycines str. 8ra, complete ... 12536/14792 (85%) 213/14792 (1%) 14621 0.0

CP000050.1 Xanthomonas campestris pv. campestris str. 8004, c... 4106/4666 (88%) 37/4666 (1%) 14617 0.0

CP023155.1 Xanthomonas citri subsp. malvacearum strain AR8100... 12524/14781 (85%) 199/14781 (1%) 14608 0.0

CP013006.1 Xanthomonas citri subsp. malvacearum strain XcmN10... 12524/14781 (85%) 199/14781 (1%) 14608 0.0

CP013004.1 Xanthomonas citri subsp. malvacearum strain XcmH10... 12524/14781 (85%) 199/14781 (1%) 14608 0.0

CP026124.1 Achromobacter sp. AONIH1 chromosome, complete genome 8233/8542 (96%) 16/8542 (0%) 14048 0.0

CP019666.1 Burkholderia cenocepacia strain VC2307 chromosome ... 8067/8547 (94%) 37/8547 (0%) 13090 0.0

HG322950.1 Pseudomonas knackmussii B13 complete genome 8649/9552 (91%) 65/9552 (1%) 12576 0.0

AJ617740.2 Pseudomonas putida clc transposon 8649/9552 (91%) 65/9552 (1%) 12576 0.0

CP008760.1 Paraburkholderia xenovorans LB400 chromosome 1, co... 8648/9552 (91%) 65/9552 (1%) 12571 0.0

CP000270.1 Paraburkholderia xenovorans LB400 chromosome 1, co... 8648/9552 (91%) 65/9552 (1%) 12571 0.0

LK391695.1 Pseudomonas pseudoalcaligenes genome assembly Ppse... 8629/9551 (90%) 64/9551 (1%) 12471 0.0

HG916826.1 Pseudomonas pseudoalcaligenes CECT 5344 complete g... 8629/9551 (90%) 64/9551 (1%) 12471 0.0

CP006985.1 Pseudomonas aeruginosa LESlike4 sequence 7903/8557 (92%) 48/8557 (1%) 12135 0.0

CP006984.1 Pseudomonas aeruginosa LESlike1 sequence 7903/8557 (92%) 48/8557 (1%) 12135 0.0

CP006983.1 Pseudomonas aeruginosa LESB65 sequence 7903/8557 (92%) 48/8557 (1%) 12135 0.0

CP006982.1 Pseudomonas aeruginosa LES400 sequence 7903/8557 (92%) 48/8557 (1%) 12135 0.0

CP006981.1 Pseudomonas aeruginosa LESlike7 sequence 7903/8557 (92%) 48/8557 (1%) 12135 0.0

CP006980.1 Pseudomonas aeruginosa LESlike5 sequence 7903/8557 (92%) 48/8557 (1%) 12135 0.0

CP006937.1 Pseudomonas aeruginosa LES431, complete genome 7903/8557 (92%) 48/8557 (1%) 12135 0.0

FM209186.1 Pseudomonas aeruginosa LESB58 complete genome sequ... 7903/8557 (92%) 48/8557 (1%) 12135 0.0

CP027538.1 Pseudomonas aeruginosa strain AR_0095 chromosome, ... 7664/8538 (90%) 51/8538 (1%) 10877 0.0

CP007787.1 Burkholderia cepacia strain DDS 7H-2 chromosome 1,... 7664/8538 (90%) 51/8538 (1%) 10877 0.0

CP009365.1 Pseudomonas mosselii SJ10, complete genome 7664/8538 (90%) 51/8538 (1%) 10877 0.0

HE798385.1 Achromobacter xylosoxidans NH44784-1996 complete g... 7664/8538 (90%) 51/8538 (1%) 10877 0.0

CU207211.1 Herminiimonas arsenicoxydans chromosome, complete ... 7664/8538 (90%) 51/8538 (1%) 10877 0.0

CP019678.1 Burkholderia cenocepacia strain VC1254 chromosome ... 7663/8538 (90%) 52/8538 (1%) 10872 0.0

CP011917.1 Burkholderia cenocepacia strain ST32 chromosome 1,... 7662/8538 (90%) 51/8538 (1%) 10866 0.0

CP008871.2 Pseudomonas aeruginosa strain W45909, complete genome 7677/8562 (90%) 70/8562 (1%) 10844 0.0

CP017353.1 Pseudomonas aeruginosa strain FA-HZ1, complete genome 7677/8562 (90%) 70/8562 (1%) 10844 0.0

CP011857.1 Pseudomonas aeruginosa strain ATCC 27853, complete... 7677/8562 (90%) 70/8562 (1%) 10844 0.0

CP015117.1 Pseudomonas aeruginosa strain ATCC 27853, complete... 7677/8562 (90%) 70/8562 (1%) 10844 0.0

CP011369.1 Pseudomonas aeruginosa strain S04 90 genome 7676/8562 (90%) 70/8562 (1%) 10839 0.0

CP025055.1 Pseudomonas aeruginosa strain PB350 chromosome, co... 7675/8562 (90%) 70/8562 (1%) 10833 0.0

CP025056.1 Pseudomonas aeruginosa strain PB367 chromosome, co... 7675/8562 (90%) 70/8562 (1%) 10833 0.0

CP010555.1 Pseudomonas aeruginosa strain FRD1, complete genome 7674/8562 (90%) 71/8562 (1%) 10826 0.0

CP008864.2 Pseudomonas aeruginosa strain W60856, complete genome 7673/8562 (90%) 70/8562 (1%) 10822 0.0

CP016955.1 Pseudomonas aeruginosa strain RIVM-EMC2982, comple... 7673/8562 (90%) 70/8562 (1%) 10822 0.0

CP011317.1 Pseudomonas aeruginosa strain Carb01 63, complete ... 7673/8562 (90%) 70/8562 (1%) 10822 0.0

Results from Blastn (v2.8.0+) analyses against the nucleotide collection (nt; 46,977,437 sequences) using the three large fragments specific to *X. fastidiosa* subsp. *pauca* Hib4 strain. Descriptions in bold are for matches with *X. fastidiosa* sequences. Green, yellow and orange backgrounds illustrate matches on 100%, more than 80% or than 70% of the query length, respectively.
