# Supplementary material for: Identification of genetic relationships and subspecies signatures in Xylella fastidiosa
Source: BMC Genomics. 2019 Mar 25;20:239. doi: 10.1186/s12864-019-5565-9 (PMC6434890; doi:10.1186/s12864-019-5565-9)
Supplement: Supplementary file 10 — Pairwise comparison of 47 Xylella sp. genomes using the occurrence of shared k-mers of length 22 bp. (DOCX 68 kb) [file 12864_2019_5565_MOESM10_ESM.docx]

**Additional File 10.** Pairwise comparison of 47 *Xylella* sp. genomes using the occurrence of shared k-mers of length 22bp.

Values in colors are as follow: red for *X. fastidiosa* subsp. *multiplex*; orange for *X. fastidiosa* subsp. *morus*; pink/brown for *X. fastidiosa* subsp. *sandyi*; light/dark green for *X. fastidiosa* subsp. *fastidiosa*; purple for *X. fastidiosa* subsp. *pauca*.
